# Endoplasmic reticulum stress signalling – from basic mechanisms to clinical applications

**DOI:** 10.1111/febs.14608

**Published:** 2018-08-04

**Authors:** Aitor Almanza, Antonio Carlesso, Chetan Chintha, Stuart Creedican, Dimitrios Doultsinos, Brian Leuzzi, Andreia Luís, Nicole McCarthy, Luigi Montibeller, Sanket More, Alexandra Papaioannou, Franziska Püschel, Maria Livia Sassano, Josip Skoko, Patrizia Agostinis, Jackie de Belleroche, Leif A. Eriksson, Simone Fulda, Adrienne M. Gorman, Sandra Healy, Andrey Kozlov, Cristina Muñoz‐Pinedo, Markus Rehm, Eric Chevet, Afshin Samali

**Affiliations:** ^1^ Apoptosis Research Centre National University of Ireland Galway Ireland; ^2^ Department of Chemistry and Molecular Biology University of Gothenburg Göteborg Sweden; ^3^ Randox Teoranta Dungloe County Donegal Ireland; ^4^ INSERM U1242 University of Rennes France; ^5^ Centre de Lutte Contre le Cancer Eugène Marquis Rennes France; ^6^ Ludwig Boltzmann Institute for Experimental and Clinical Traumatology AUVA Research Centre Vienna Austria; ^7^ Institute for Experimental Cancer Research in Paediatrics Goethe‐University Frankfurt Germany; ^8^ Neurogenetics Group Division of Brain Sciences Faculty of Medicine Imperial College London UK; ^9^ Department Cellular and Molecular Medicine Laboratory of Cell Death and Therapy KU Leuven Belgium; ^10^ Cell Death Regulation Group Oncobell Program Bellvitge Biomedical Research Institute (IDIBELL) Barcelona Spain; ^11^ Institute of Cell Biology and Immunology University of Stuttgart Germany

**Keywords:** endoplasmic reticulum, proteostasis, signalling pathway, stress

## Abstract

The endoplasmic reticulum (ER) is a membranous intracellular organelle and the first compartment of the secretory pathway. As such, the ER contributes to the production and folding of approximately one‐third of cellular proteins, and is thus inextricably linked to the maintenance of cellular homeostasis and the fine balance between health and disease. Specific ER stress signalling pathways, collectively known as the unfolded protein response (UPR), are required for maintaining ER homeostasis. The UPR is triggered when ER protein folding capacity is overwhelmed by cellular demand and the UPR initially aims to restore ER homeostasis and normal cellular functions. However, if this fails, then the UPR triggers cell death. In this review, we provide a UPR signalling‐centric view of ER functions, from the ER's discovery to the latest advancements in the understanding of ER and UPR biology. Our review provides a synthesis of intracellular ER signalling revolving around proteostasis and the UPR, its impact on other organelles and cellular behaviour, its multifaceted and dynamic response to stress and its role in physiology, before finally exploring the potential exploitation of this knowledge to tackle unresolved biological questions and address unmet biomedical needs. Thus, we provide an integrated and global view of existing literature on ER signalling pathways and their use for therapeutic purposes.

## Graphical abstract

The current article reviews the most up‐to‐date literature on Endoplasmic Reticulum (ER) biology and articulates this information from a signalling perspective. Not only do we cover the basic cell biology aspects of adaptive ER signalling but also provide information about the latest discoveries on ER stress targeting drugs and their potential use in the clinic.

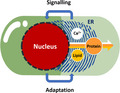

**Abbreviations**
4‐PBA: 4‐phenylbutyric acid
ALS: amyotrophic lateral sclerosis
ATF4: activating transcription factor 4
ATF6f: cytosolic domain of ATF6
ATF6α: activating transcription factor 6 α
ATF6β: activating transcription factor 6 β
BBF2H7: cAMP responsive element‐binding protein 3 like 2
BiP: binding immunoglobulin protein (gene *GRP78*)
bZIP: basic‐leucine zipper
CHOP: CAAT/enhancer‐binding protein (C/EBP) homologous protein
CRCL: chaperone‐rich cell lysate
CREB3L3: cAMP responsive element‐binding protein 3 like 3
CREB: cAMP response element‐binding protein
eIF2B: eukaryotic translation initiation factor 2B
eIF2α: eukaryotic translation initiation factor 2α
ERAD: ER‐associated protein degradation
ER: endoplasmic reticulum
ERN1: endoplasmic reticulum to nucleus signalling 1
ERN2: endoplasmic reticulum to nucleus signalling 2
ERO‐1: ER oxidoreductin 1
ERα: oestrogen receptor α
GADD34: growth arrest and DNA‐damage‐inducible 34
GRP78: glucose‐regulated protein 78
GSH: glutathione
IBD: inflammatory bowel disease
IRE1α: inositol‐requiring enzyme 1 α
IRE1β: inositol‐requiring enzyme 1 β
LUMAN: cAMP responsive element‐binding protein 3 or CREB3
MAM: mitochondria‐associated membrane
MBTPS1: membrane bound transcription factor peptidase, site 1
MBTPS2: membrane bound transcription factor peptidase, site 2
MDM1/SNX13: mitochondrial distribution and morphology 1/sorting nexin 13
mTOR: mammalian target of rapamycin
N‐ATF6: N‐terminal portion of ATF6 or ATF6f
NF‐Y: nuclear transcription factor Y
NGLY1: *N*‐glycanase
NPR: NADPH‐P450 reductase
OASIS: cAMP responsive element‐binding protein 3 like 1
ORAI1: calcium release‐activated calcium channel protein 1
PDI: protein disulfide isomerase
p‐eIF2α: phospho‐eIF2α
PERK: protein kinase RNA‐like (PKR‐like) endoplasmic reticulum kinase
PKR: protein kinase RNA‐activated
PM: plasma membrane
PP1: protein phosphatase type 1
qPCR: quantitative polymerase chain reaction
RER: rough endoplasmic reticulum
RIDD: regulated IRE1‐dependent decay
ROS: reactive oxygen species
SEC22b: vesicle‐trafficking protein SEC22b
SERCA: sarco/endoplasmic reticulum ATPase Ca^2+^‐ATPase
SER: smooth endoplasmic reticulum
TAD: transcriptional activation domain
TRAF2: tumour necrosis factor receptor‐associated factor 2
TUDCA: tauroursodeoxycholic acid
UDCA: ursodeoxycholic acid
UPR: unfolded protein response
WT: wild‐type
XBP1s: spliced isoform of XBP1
XBP1u: unspliced isoform of XBP1
XBP1: X‐box binding protein 1

## Introduction

The endoplasmic reticulum (ER) is a cellular organelle that was first visualized in chicken fibroblast‐like cells using electron microscopy and was described as a ‘delicate lace‐work extending throughout the cytoplasm’ 1. Its current name was coined almost 10 years later by Porter in 1954 2. The ER appears as a membranous network of elongated tubules and flattened discs that span a great area of the cytoplasm 3. This membrane encloses the ER lumen and allows for the transfer of molecules to and from the cytoplasm.

## ER structure

**Figure 1:**
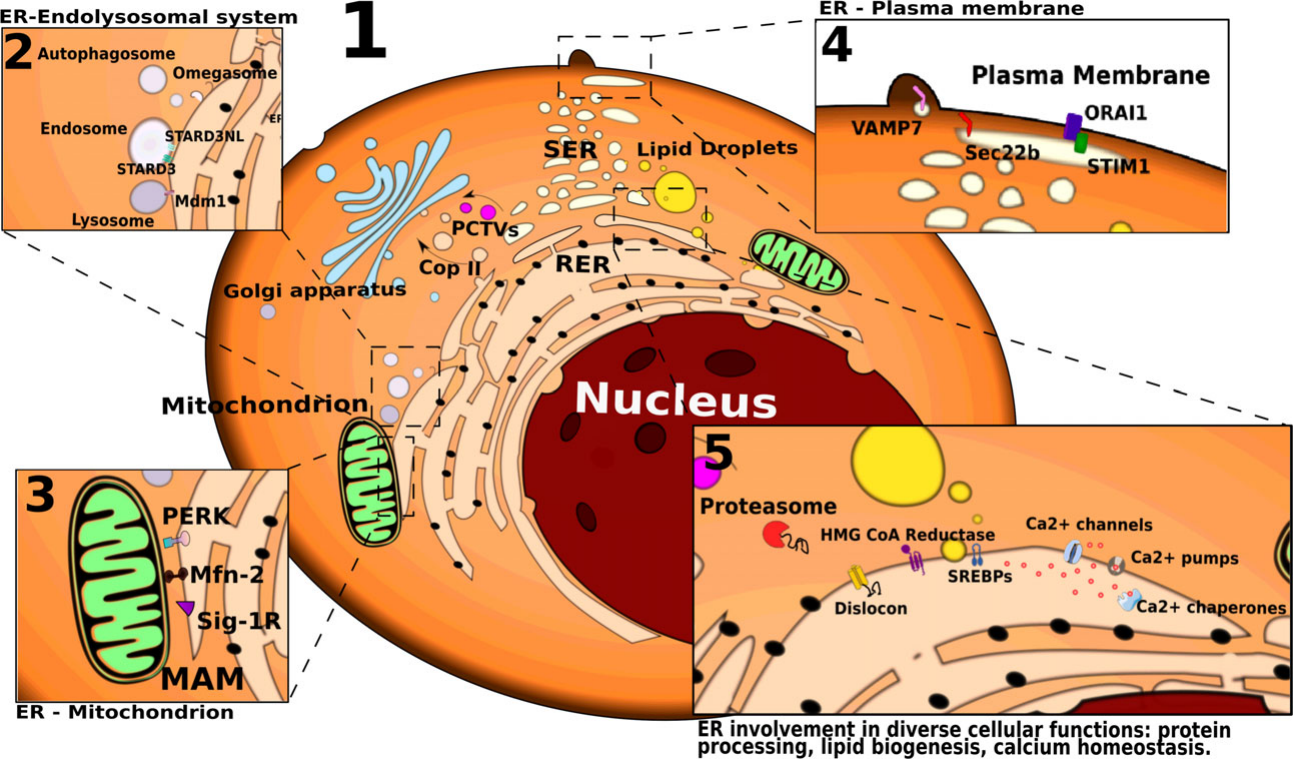
ER molecular machines and contact sites with other organelles. The ER is primarily subdivided into the SER and RER, with the latter characterized by the presence of ribosomes at its cytosolic surface. Alternatively, the ER has been recently classified into the nuclear envelope, ER sheet‐like cisternae and tubular ER (panel 1). The ER forms multiple membrane contact sites with other organelles, including the endosomes and lysosomes (through STARD3, STARD3NL, Mdm1; panel 2), the mitochondria (through Mfn‐2, Sig‐1R, PERK; panel 3), and the PM (through ORAI1, STIM1, Sec22b, VAMP7; panel 4) with various functional implications. The ER plays instrumental roles in secretory and transmembrane protein folding and quality control, protein and lipid trafficking, lipid metabolism, and Ca^2+^ homeostasis, all of these processes being mediated by a diverse series of ER resident proteins (schematically depicted in panels 1 and 5).

The ER is classically divided into the rough ER (RER) and smooth ER (SER), depending on the presence or absence of ribosomes on the cytosolic face of the membrane respectively. The SER and RER can exist either as interconnected or spatially separated compartments 4. More recently, a novel classification was proposed based on membrane structure rather than appearance. According to this classification, the ER comprises the nuclear envelope, sheet‐like cisternae and a polygonal array of tubules connected by three‐way junctions 5. A striking difference between these ER structures is the curvature of the membrane, whereby ER tubules possess a high membrane curvature compared to the sheets of the nuclear envelope and cisternae. The ER occupies an extensive cell‐type‐specific footprint within the cell and is in contact with many other intracellular organelles. It forms physical contact sites with mitochondria named mitochondria‐associated membranes (MAMs), which play a crucial role in Ca^2+^ homeostasis 6. It also comes in contact with the plasma membrane (PM), an interaction regulated by proteins like stromal interaction molecule 1 in the ER and calcium release‐activated calcium channel protein 1 in the PM which are controlled by Ca^2+^ levels 7. Vesicle‐trafficking protein SEC22b (SEC22b) and vesicle‐associated membrane protein 7 are also involved in the stabilization of ER‐PM contacts and PM expansion 8. The ER also interacts with endosomes 9 and is tethered by StAR‐related lipid transfer protein 3 and StAR‐related lipid transfer protein 3 10, which also contribute to cholesterol maintenance in endosomes 11. Interestingly, an ER interaction with the endolysosomal system, mediated by the mitochondrial distribution and morphology 1/sorting nexin 13 (MDM1/SNX13) complex 12, suggests ER involvement in autophagy. Indeed, a specialized ER structure called the omegasome forms contact sites with the phagophore, which elongates and becomes a mature autophagosome 13, 14 (Fig. 1). In this way, the ER on its own or in coordination with other cell organelles exerts its multifaceted roles in the functionality of the cell as it is discussed in the next sections.

## ER functions

The ER is involved in many different cellular functions. It acts as a protein synthesis factory, contributes to the storage and regulation of calcium, to the synthesis and storage of lipids, and to glucose metabolism 3. These diverse functions indicate a pivotal role for the ER as a dynamic ‘nutrient sensing’ organelle that coordinates energetic fluctuations with metabolic reprogramming responses, regulating metabolism and cell fate decisions (Fig. 1).

### Protein folding and quality control

The ER is involved in secretory and transmembrane protein synthesis, folding, maturation, quality control and degradation, and ensures that only properly folded proteins are delivered to their site of action 15. About 30% of all proteins are cotranslationally targeted to the ER 16 where they are exposed to an environment abundant in chaperones and foldases that facilitate their folding, assembly and post‐translational modification before they are exported from the ER 16. Protein processing within the ER includes signal sequence cleavage, N‐linked glycosylation, formation, isomerization or reduction of disulfide bonds [catalysed by protein disulfide isomerases (PDIs), oxidoreductases], isomerization of proline or lipid conjugation, all of which ultimately result in a properly folded conformation 16, 17, 18, 19. Misfolded proteins are potentially detrimental to cell function and are therefore tightly controlled. Although protein misfolding takes place continually, it can be exacerbated during adverse intrinsic and environmental conditions. The ER has developed quality control systems to ensure that there are additional opportunities to correct misfolded proteins or, if terminally misfolded, to be disposed of by the cell. Terminally misfolded secretory proteins are eliminated by a process called ER‐associated degradation (ERAD) 20. Proteins are first recognized by an ER resident luminal and transmembrane protein machinery, then retrotranslocated into the cytosol by a channel named dislocon 21 and the cytosolic AAA+ ATPase p97 22, deglycosylated by *N*‐glycanase (NGLY1; 23) and targeted for degradation via the ubiquitin–proteasome pathway 20, 24, 25 (Fig. 1).

### Lipid synthesis

The ER also plays essential roles in membrane production, lipid droplet/vesicle formation and fat accumulation for energy storage. Lipid synthesis is localized at membrane interfaces and organelle contact sites, and the lipid droplets/vesicles are exported in a regulated fashion. The ER dynamically changes its membrane structure to adapt to the changing cellular lipid concentrations. The ER contains the sterol regulatory element‐binding protein family of cholesterol sensors ensuring cholesterol homeostasis 26. This compartment also hosts enzymes catalysing the synthesis of cell membrane lipid components, namely sterols, sphingolipids and phospholipids 27. The synthesis of those lipids from fatty acyl‐CoA and diacylglycerols takes place at the ER membrane 28, which also hosts 3‐hydroxy‐3‐methyl‐glutaryl‐coenzyme A reductase, the rate‐limiting enzyme of the mevalonate/isoprenoid pathway that produces sterol and isoprenoid precursors 29. Precursors made by ER membrane‐localized enzymes are subsequently converted into structural lipids, sterols, steroid hormones, bile acids, dolichols, prenyl donors and a myriad of isoprenoid species with key functions for cell metabolism. Interestingly, MAMs have been identified as a privileged site of sphingolipid synthesis 30 (Fig. 1).

### ER export

Most of the proteins and lipids synthesized in the ER must be transported to other cellular structures, which occurs mostly through the secretory pathway. To maintain the constant anabolic flux, export needs to be tightly regulated, and defects in secretion can lead to serious structural and functional consequences for the ER. Central to this export process is the generation of ER COPII transport vesicles, named after the family of proteins that shapes and coats them 31. In addition to COPII vesicle transport, several other mechanisms of lipid export have been described. A variety of lipids can be transported by nonvesicular mechanisms; for example, large lipoprotein cargo has been shown to be exported out of the ER in another type of vesicle termed prechylomicron transport vesicles 32 or to accumulate in lipid droplets (Fig. 1).

### Ca^2+^ homeostasis

Ca^2+^ is involved as a secondary messenger in many intracellular and extracellular signalling networks, playing an essential role in gene expression, protein synthesis and trafficking, cell proliferation, differentiation, metabolism or apoptosis 33. ER, as the main cellular compartment for Ca^2+^ storage, plays a pivotal role in the regulation of Ca^2+^ levels and reciprocally many ER functions are controlled in a Ca^2+^‐dependent way, thereby regulating the calcium homeostasis of the whole cell 34. Consequently, both ER and cytosolic Ca^2+^ concentrations need to be highly spatiotemporally regulated in order for the ER to maintain a much increased physiological intraluminal Ca^2+^ concentration and oxidizing redox potential than the cytoplasm. To modulate these levels, the ER employs a number of mechanisms that control Ca^2+^ concentration on both sides of the membrane: (a) ER membrane ATP‐dependent Ca^2+^ pumps for cytosol‐to‐lumen transport; (b) ER luminal Ca^2+^‐binding chaperones for sequestering free Ca^2+^; and (c) ER membrane channels for the regulated release of Ca^2+^ into the cytosol. These mechanisms are facilitated by a tight communication between the ER and other organelles, such as the PM and the mitochondria, thereby supporting the cell needs.

Traditionally thought as a site of protein synthesis, recent evidence has established the involvement of the ER in many different cellular functions: from novel roles in lipid metabolism to connections with cytoskeletal structures or roles in cytoplasmic streaming, our view of the ER keeps rapidly expanding, placing it increasingly as a key organelle governing the whole cellular metabolism.

## Perturbing ER functions

Conditions that disrupt ER homeostasis create a cellular state commonly referred to as ‘ER stress’. The cellular response to ER stress involves the activation of adaptive mechanisms to overcome stress and restore ER homeostasis. This response is dependent on the perturbing agent/condition and the intensity/duration of the stress 35.

### Intrinsic ER perturbations

Cell autonomous mechanisms can lead to ER perturbation and examples of this can be seen in several diseases, including cancer, neurodegenerative diseases and diabetes. The hallmarks of cancer such as genetic instability and mutations 36 can result in constitutive activation of ER stress response pathways leading to cell growth, proliferation, differentiation and migration. In addition, the uncontrolled, rapid growth of cancer cells requires high protein production rates with a consequent impact on ER systems 37. Many cancers have a high mutation load which results in an intrinsically higher level of ER stress. For example, melanoma has the highest mutation burden of any cancer and the sheer numbers of mutated proteins are a source of intrinsically higher ER stress levels. In chronic myeloid leukaemia, the fusion protein produced the Philadelphia chromosome, BCR‐ABL1, is a constitutively active oncoprotein that enhances cell proliferation and interferes with Ca^2+^‐dependent apoptotic response 38. In addition, mutation‐driven ER stress can also induce senescence that contributes to chemoresistance 39. ER stress has also been linked to several neurodegenerative diseases. For example, mutations in the ER resident vesicle‐associated membrane protein‐associated protein B in familial amyotrophic lateral sclerosis (ALS) are linked to induction of motor neuron death mediated by the alteration of ER stress signalling 40, 41. On the other hand, secretory cells such as pancreatic β cells have a highly developed ER to manage insulin production and release in response to increases in blood glucose. The C96Y insulin variant leads to its impaired biogenesis and ER accumulation in the Akita mouse. As the ER cannot cope with the mutation induced stress, beta cells die and type 1 diabetes develops 42, 43. Insulin mutation‐related ER stress was also reported in neonatal diabetes 44, 45.

### Extrinsic perturbations

#### Microenvironmental stress

In tumours, the ER stress observed in rapidly proliferating cells is compounded by the fact that increased proliferation eventually depletes the microenvironment of nutrients and oxygen, causing local microenvironmental stress and resulting in hypoxia, starvation and acidosis, all of which cause ER stress and perturb protein, and possibly lipid synthesis 46. Nutrient deprivation, and particularly glucose starvation, at least in part, promotes ER stress by impairing glycosylation.

#### Exposure to ER stressors

Several small molecules that induce ER stress through a variety of mechanisms have been identified 47, 48. Stressors such as tunicamycin 49, 50, or 2‐deoxyglucose 51 target the N‐linked glycosylation of proteins, whereas dithiothreitol inhibits protein disulfide bond formation^52^. Alternatively, Brefeldin A impairs ER‐to‐Golgi trafficking, thus causing a rapid and reversible inhibition of protein secretion 53. Targeting the Sarco/ER Ca^2+^‐ATPase (SERCA) with compounds, such as thapsigargin and cyclopiazonic acid 54, 55, induces ER stress by reducing ER Ca^2+^ concentration and impairing protein folding capacity.

#### Exposure to enhancers of ER homeostasis

Conversely, other molecules have been found that can alleviate ER stress. These include small molecules, peptides and proteostasis regulators. The frequently used 4‐phenylbutyric acid (4‐PBA) reduces the accumulation of misfolded proteins in the ER 56. Tauroursodeoxycholic acid (TUDCA) is an endogenous bile acid able to resolve ER stress in islet cells 57. TUDCA is the taurine conjugate of ursodeoxycholic acid (UDCA), an FDA‐approved drug for primary biliary cirrhosis that is also able to alleviate ER stress 58. The precise mode of action of such proteostasis modulators still remains elusive.

#### Temperature

Body temperature is crucial for the viability of metazoans; normal mammalian physiological temperatures are 36–37 °C. Deviations from this range can disrupt cellular homeostasis causing protein denaturation and/or aggregation 59. Moreover, an acute increase in temperature, known as heat shock, causes the fragmentation of both ER and Golgi 59. Heat preconditioning at mildly elevated temperatures (up to 40 °C) in mammalian cellular and animal models has been shown to lead to the development of thermotolerance, which is associated with an increase in the expression of several heat shock proteins and ER stress markers 60, 61. In addition, moderate hypothermia (28 °C) induces mild ER stress in human pluripotent stem cells, the activation of which may be sufficient to protect against severe stress through an effect known as ER hormesis 62, 63.

#### Reactive oxygen species production and other perturbations

Several external agents can induce intracellular reactive oxygen species (ROS) production, and when ROS production exceeds the antioxidant capacity oxidative stress negatively affects protein synthesis and ER homeostasis 64. ROS, including free radicals, are generated by the UPR‐regulated oxidative folding machinery in the ER 65 and in the mitochondria 66. In this context, increased mitochondrial respiration and biogenesis promotes survival during ER stress through a reduction of ROS 67. The ER provides an oxidizing environment to facilitate disulfide bond formation and this process is believed to contribute to as much as 25% of the overall ROS generated 68, 69. The interconnection between the ER and ROS is mediated by signalling pathways which involve glutathione (GSH)/glutathione disulfide, NADPH oxidase 4, NADPH‐P450 reductase, Ca^2+^, ER oxidoreductin 1 (ERO1) and PDI 70. The latter, in particular, has been found upregulated in the central nervous system of Alzheimer's disease patients thus highlighting the relevance of these pathways in neurodegenerative disease 71. Overall, from the sections above it is apparent that directly or indirectly impaired ER function contributes to disease development and treatment resistance.

## ER stress consequences

In response to ER stress, cells trigger an adaptive signalling pathway called the unfolded protein response (UPR), which acts to help cells to cope with the stress by attenuating protein synthesis, clearing the unfolded/misfolded proteins and increasing the capacity of the ER to fold proteins.

### The UPR

**Figure 2:**
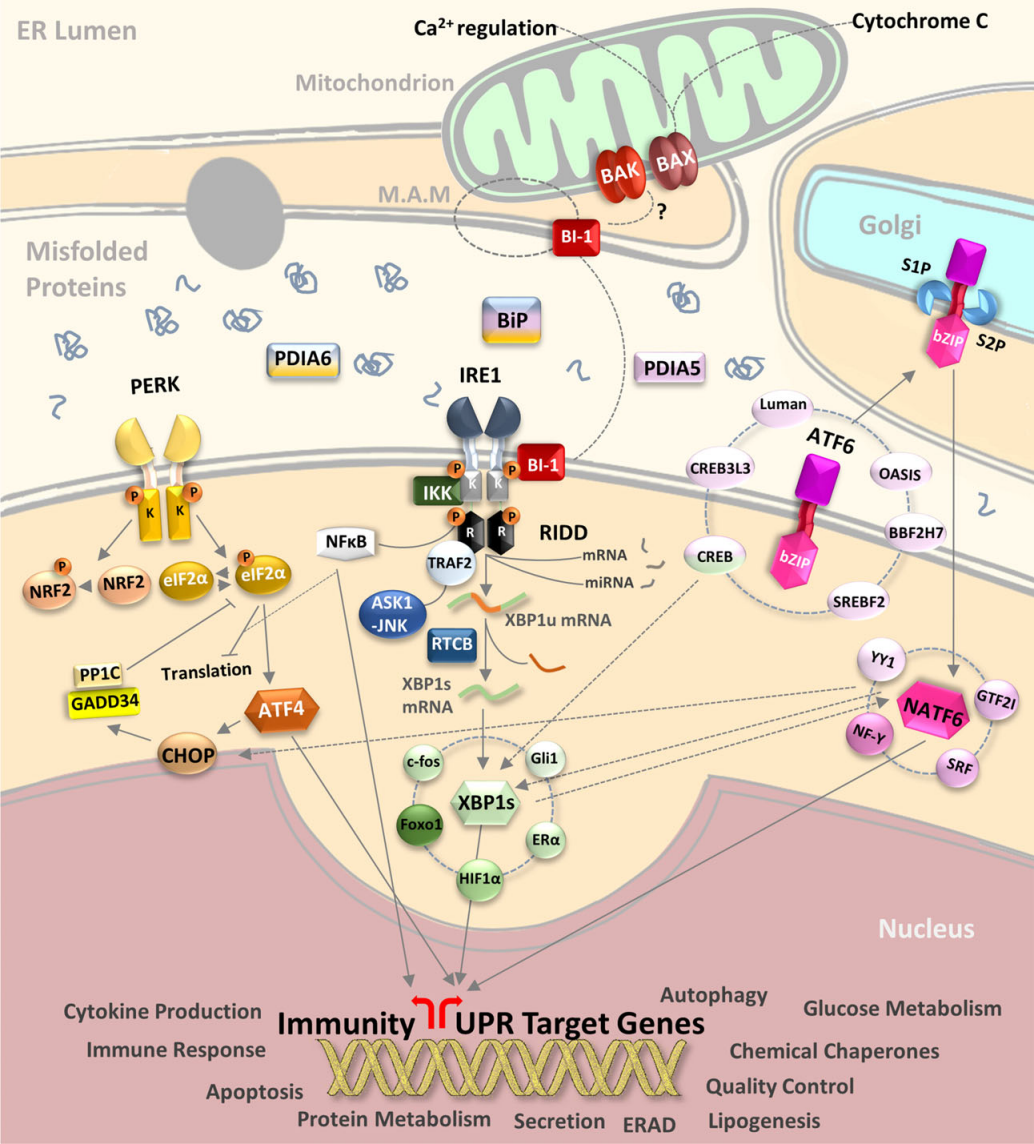
Signalling the UPR and downstream pathways. The 3ER stress sensors (PERK, IRE1, ATF6) upon release from BiP, PDIA5, 6 initiate signalling cascades through transcription factor production (ATF4, XBP1s, ATF6f) and associated processes such as RIDD, NFκB activation and ERAD to address the misfolded protein load on the ER. By modulating transcriptional output and translational demand the UPR attempts to re‐establish ER protein folding homeostasis and promote cell survival. If ER stress cannot be resolved then mechanisms are triggered to promote cell death.

The UPR is a cellular stress response originating in the ER and is predominantly controlled by three major sensors: inositol requiring enzyme 1 (IRE1), protein kinase RNA‐activated (PKR)‐like ER kinase (PERK) and activating transcription factor 6 (ATF6). The ER luminal domains of all three ER stress sensors are normally bound by the ER resident chaperone, heat shock protein A5 [heat shock protein family A (Hsp70) member 5, also known as glucose‐regulated protein 78 (GRP78) and binding immunoglobulin protein (gene *GRP78*) (BiP)], keeping them in an inactive state 72, 73. Accumulating misfolded proteins in the ER lumen engage BiP thus releasing the three sensors. A FRET UPR induction assay, developed to quantify the association and dissociation of the IRE1 luminal domain from BiP upon ER stress 74, demonstrated that the ER luminal co‐chaperone ERdj4/DNAJB9 represses IRE1 by promoting a complex between BiP and the luminal stress‐sensing domain of IRE1α 75. Moreover, it has recently been reported that another ER luminal chaperone, Hsp47, displaces BiP from the IRE1 UPRosome to promote its oligomerization 76. Once released from BiP, IRE1 and PERK homodimerize or oligomerize and trans‐autophosphorylate to activate their downstream pathways 72. In contrast, BiP dissociation from AFT6 reveals an ER export motif 73 which facilitates its translocation to the Golgi apparatus 77. This ‘competition model’ of UPR activation assumes that BiP acts as a negative regulator of UPR signalling. However, other BiP‐dependent or independent models have been proposed (reviewed in 78; Fig. 2).

#### IRE1 signalling

In humans, there are two paralogues of IRE1 (IRE1α and β), encoded by endoplasmic reticulum to nucleus signalling 1 and 2 (*ERN1* and *ERN2*), respectively 79, 80, 81. Both human IRE1 isoforms share significant sequence homology (39%) 20. IRE1α (referred to IRE1 hereafter) is ubiquitously expressed; however, inositol‐requiring enzyme 1 β (IRE1β) expression is restricted mainly to the gastrointestinal tract and the pulmonary mucosal epithelium 82, 83. *Ern1* knockout (KO) in mice is embryonic lethal due to growth retardation and defects in liver organogenesis and placental development 84 while *Ern2* KO mice develop colitis of increased severity and shorter latency 82 but are otherwise histologically indistinguishable from the *Ern2*WT mice. BiP dissociation, caused by accumulating unfolded proteins, triggers IRE1 oligomerization and activation of its cytosolic kinase domain. The oligomers position in close proximity, in a face‐to‐face orientation, enabling trans‐autophosphorylation. This face‐to‐face configuration is adopted by both human and murine IRE1 85, 86. Phosphorylation in the activation loop of the kinase domain, specifically at Ser724, Ser726 and Ser729, is not only necessary to activate its cytosolic RNase domain 87 but is also required to initiate recruitment of tumour necrosis factor receptor‐associated factor 2 (TRAF2) and JNK pathway signalling 88. The IRE1 cytosolic domain, which is highly homologous with RNase L 89, induces a selective cleavage of dual stem loops within the X‐box binding protein 1 (XBP1) mRNA 79, 90, 91. Therefore, IRE1, in a spliceosome independent‐manner, but together with the tRNA ligase RNA 2′,3′‐cyclic phosphate and 5′‐OH ligase 92, 93, 94, 95, 96, 97, catalyses the splicing of a 26 nucleotide intron from human XBP1 mRNA to produce spliced isoform of XBP1 (XBP1s) 90, 91. XBP1s is a basic leucine zipper (bZIP) transcription factor 98, 99, 100 and the unspliced isoform of XBP1 (XBP1u) is unable to activate gene expression due to lack of a transactivation domain 91. The N‐terminal region of XBP1u contains a basic region and a leucine zipper domain involved in dimerization and DNA binding 91, 98, 100, 101. The XBP1u C‐terminal region contains a P (proline), E (glutamic acid), S (serine) and T (threonine) motif which destabilizes proteins (ubiquitin‐dependent proteolysis) and contributes to its short half‐life 98, 101, 102, 103. The N‐terminal region also contains two other domains: a hydrophobic region that targets XBP1u to the ER membrane and a domain that promotes efficient XBP1 splicing 104, 105, 106 and cleavage 103 by pausing XBP1 translation. IRE1‐mediated splicing of XBP1 mRNA results in an open reading frame‐shift inducing the expression of a transcriptionally active and BP1s 90, 91, 101. XBP1u has been reported to negatively regulate XBP1s transcriptional activity as well as to promote the recruitment of its own mRNA to the ER membrane through the partial translation of its N‐terminal region 107, 108. XBP1s directs the transcription of a wide range of targets including the expression of chaperones, foldases and components of the ERAD pathway, in order to relieve ER stress and restore homeostasis 109, 110. However, XBP1s can also participate in the regulation of numerous metabolic pathways such as lipid biosynthesis 111, 112, 113, glucose metabolism 114, 115, 116, 117, 118, insulin signalling 117, 119, 120, redox metabolism 121, DNA repair 122 and it influences cell fate including cell survival 123, cell differentiation 124, 125, 126, 127, 128 and development 126, 129, 130, 131. Although there is strong evidence pointing to a key role for XBP1 in multiple cellular functions, the exact mechanisms by which XBP1 mediates gene transactivation are still elusive. Indeed, in addition to the known interaction of the XBP1s transactivation domain with RNA polymerase II, other mechanisms could exist. For example, XBP1 can physically interact with many other transcription factors such as AP‐1 transcription factor subunit 132, oestrogen receptor α (ERα) 133, GLI‐family zinc finger 1 134, SSX family member 4 134, forkhead box O1 114, ATF6 135, cAMP response element‐binding protein (CREB)/ATF 135 and hypoxia inducible factor 1 alpha subunit 136 (Fig. 2).

The RNase activity of IRE1 can also efficiently target other transcripts through a mechanism called regulated IRE1‐dependent decay (RIDD) 137. Analysis of the *in vitro* RNase activity of wild‐type (WT) vs mutant IRE1 led to the discovery of a broad range of other IRE1 substrates 138, 139 and, interestingly, it was noted that IRE1 can also degrade its own mRNA 140. RIDD is a conserved mechanism in eukaryotes 137, 141, 142, 143, 144, 145 by which IRE1 cleaves transcripts containing the consensus sequence (CUGCAG) accompanied by a stem‐loop structure 142, 146. The cleaved RNA fragments are subsequently rapidly degraded by cellular exoribonucleases 141, 147. RIDD is required for the maintenance of ER homeostasis by reducing ER client protein load through mRNA degradation 137, 141, 142. Recently, it has been proposed that there is basal activity of RIDD 138 which increases progressively with the severity of ER stress. However, this hypothesis needs further experimental validation. Interestingly, IRE1β was found to selectively induce translational repression through the 28S ribosomal RNA cleavage 81 demonstrating that IRE1α and IRE1β display differential activities 148. Characterizing RIDD activity, particularly *in vivo*, has proven difficult due to the complex challenge of separating the RIDD activity from the XBP1 splicing activity of IRE1. In addition, basal RIDD can only target specific mRNA substrates, as full activation and subsequent targeting of further transcripts requires strong ER stress stimuli (Fig. 2).

#### PERK signalling

PERK was identified in rat pancreatic islets as a serine/threonine kinase and, similar to PKR, heme regulated initiation factor 2 alpha kinase and general control nonderepressible 2, can phosphorylate eIF2α 149, 150. PERK is ubiquitously expressed in the body 149 and has an ER luminal domain as well as a cytoplasmic kinase domain 150. BiP detachment from the ER luminal domain leads to oligomerization 72, trans‐autophosphorylation and activation of PERK 151. Active PERK phosphorylates eIF2α on serine 51 150. eIF2α is a subunit of the eIF2 heterotrimer 152, 153 which regulates the first step of protein synthesis initiation by promoting the binding of the initiator tRNA to 40S ribosomal subunits 154. However, eIF2α phosphorylation by PERK inhibits eukaryotic translation initiation factor 2B (eIF2B) activity and thereby downregulates protein synthesis 155. Blocking translation during ER stress consequently reduces the protein load on the ER folding machinery 156.

Remarkably, some transcripts are translated more efficiently during PERK‐dependent global repression of translation initiation. The ubiquitously expressed activating transcription factor 4 (ATF4) 157, whose transcript contains short upstream open reading frames (uORFs) 158, is normally inefficiently translated from the protein‐coding AUG 159. However, attenuation of translation from uORFs shifts translation initiation towards the protein coding AUG, resulting in more efficient synthesis of ATF4 158. ATF4 can then bind to the C/EBP‐ATF site in the promoter of CAAT/enhancer‐binding protein (C/EBP) homologous protein (CHOP)/GADD153 160 and induce its expression 158. ATF4 and CHOP directly induce genes involved in protein synthesis and the UPR 161, but conditions under which ATF4 and CHOP increase protein synthesis can result in ATP depletion, oxidative stress and cell death 162. eIF2α phosphorylation (p‐eIF2α) can also directly enhance the translation of CHOP 163, 164 and other proteins involved in the ER stress response, as reviewed in 165. For example, growth arrest and DNA‐damage‐inducible 34 (GADD34) 166, 167 is positively regulated by eIF2α phosphorylation 168 and likewise transcriptionally induced by ATF4 169 and CHOP 170. Interestingly, GADD34 interacts with the catalytic subunit of type 1 protein serine/threonine phosphatase (PP1) 171, which dephosphorylates eIF2α thereby creating a negative feedback loop that antagonizes p‐eIF2α‐dependent translation inhibition and restores protein synthesis 169, 170, 172. The translational arrest induced by p‐eIF2α reduces protein load in ER lumen and conserves nutrients, while ATF4 driven expression of adaptive genes involved in amino acid transport and metabolism, protection from oxidative stress, protein homeostasis and autophagy together help the cell to cope with ER stress 173, 174. However, sustained stress changes the adaptive response to a prodeath response and ultimately, the phosphorylation status of eIF2α appears to codetermine the balance between prosurvival or prodeath signalling 175, 176. This is accomplished by the above mentioned delayed feedback through which the interplay of GADD34, ATF4 and CHOP results in the activation of genes involved in cell death, cell‐cycle arrest and senescence 177, 178, 179, 180 (Fig. 2).

#### ATF6 signalling

The transcription factor ATF6, which belongs to an extensive family of leucine zipper proteins 8, is encoded in humans by two different genes: *ATF6A* for ATF6α 181 and *ATF6B* for ATF6β 153. After its activation in the ER and export to the Golgi, it is cleaved by the two Golgi‐resident proteases membrane bound transcription factor peptidase, site 1 (MBTPS1) and MBTPS1, releasing a fragment of ~ 400 amino acids corresponding to ATF6 cytosolic N‐terminal portion (ATF6f). ATF6f comprises a transcriptional activation domain (TAD), a bZIP domain, a DNA‐binding domain and nuclear localization signals. In the nucleus, ATF6f induces UPR gene expression 73, 182. Although the two ATF6 paralogs share high homology 153, ATF6β is a very poor activator of UPR genes due to the absence of eight important amino acids in the TAD domain 157. Indeed, it rather seems to function as an inhibitor by forming heterodimers with ATF6α 10, 158. Interestingly, ATF6 can modulate gene expression by interacting with other bZIPs, such as CREB 159, cAMP responsive element‐binding protein 3 like 3 (CREB3L3) 160, sterol regulatory element‐binding transcription factor 2 161 and XBP1 71, and various other transcription factors such as serum response factor 181, components of the nuclear transcription factor Y (NF‐Y) complex 159, 162, 163, yin yang 1 163, 164 and general transcription factor I 165. Converging with IRE1 and PERK signalling cascades, ATF6 can also induce the expression of XBP1 and CHOP to enhance UPR signalling 30, 166, 167. However, ATF6 is not the only ER‐resident bZIP transcription factor. At least five other tissue‐specific bZIPs, named Luman, cAMP responsive element‐binding protein 3 like 1 (OASIS), cAMP responsive element‐binding protein 3 like 2 (BBF2H7), CREB3L3 and CREB, reviewed in 183, are involved in ER stress signalling (Fig. 2), highlighting the regulatory complexity this branch of the ER stress response is subjected to at the organismal level.

#### Noncoding RNAs

Noncoding RNAs are connected to the three UPR sensors with effects on both physiological and pathological conditions 184. These RNA species mostly include microRNAs (miRNAs) and also long noncoding RNAs (lncRNAs). This additional level of regulation works in fact in a bidirectional manner. This means that either the UPR sensors themselves or their downstream components can also modulate their expression levels. A certain number of miRNAs have been so far recognized to regulate IRE1, which in turn regulates miRNAs through XBP1s at a transcriptional level and through RIDD activity via degradation. One miRNA regulates PERK expression, while this in turn regulates miRNAs through its downstream targets. ATF6 is also modulated by miRNAs, but only one miRNA has been found under its direct effect. Upstream of IRE1, PERK and ATF6, the BiP chaperone is also regulated by miRNAs but does not control any. In addition to miRNAs, lncRNAs exhibit a similar role regarding the regulation of UPR factors and vice versa. Their levels change in accordance to the cell stress status and depending on the pathophysiological context lead to distinct cell fates. This interconnection between noncoding RNAs and the UPR may contribute to a more complex network but at the same time reveals the existence of fine‐tuning mechanisms governing ER stress responses and their effects in cell homeostasis (described in 184).

### Proximal impact of UPR activation

#### Transcriptional programmes

Each branch of the UPR pathway culminates in transcriptional regulation and, together the UPR's major transcription factors, ATF6f, XBP1s and ATF4, stimulate many adaptive responses to restore ER function and maintain cell survival 35. They regulate genes encoding ER chaperones, ERAD factors, amino acid transport and metabolism proteins, phospholipid biosynthesis enzymes, and numerous others 185. In particular, the IRE1–XBP1 pathway is involved in the induction of ER chaperones and capacity control of ERAD 186 as well as promoting cytoprotection 187 and cleaving miRNAs that regulate the cell death‐inducing caspases 188. ATF6f translocates to the nucleus where it activate genes involved in protein folding, processing, and degradation 185. ATF4, activated downstream of PERK and p‐eIF2α, increases the transcription of many genes that promote survival under ER stress. Some of these prosurvival genes include genes that are involved in redox balance, amino acid metabolism, protein folding and autophagy 189.

#### Translational programmes

Translation is directly impacted by UPR activation under ER stress conditions, particularly by PERK as described above. It also affects the expression of several miRNAs, which may further contribute to translation attenuation or protein synthesis 35. It has been shown that ER stress can regulate the execution phase of apoptosis by causing the transient induction of inhibitor of apoptosis proteins (IAPs). Several papers have reported that cIAP1, cIAP2 and XIAP are induced by ER stress, and that this induction is important for cell survival, as it delays the onset of caspase activation and apoptosis. PERK induction of cIAPs and the transient activity of PI3K–AKT signalling suggest that PERK not only allows adaptation to ER stress, but it also actively inhibits the ER stress‐induced apoptotic programme 190.

#### Protein degradation

There are two main protein degradation pathways activated by components of the UPR following ER stress: ubiquitin–proteasome‐mediated degradation via ERAD and lysosome‐mediated protein degradation via autophagy. ERAD is responsible for removing misfolded proteins from the ER and several genes involved in ERAD are upregulated by ATF6f and XBP1s 185. ERAD involves the retrotranslocation of misfolded proteins from the ER into the cytosol where they are degraded by the proteasome (see above) 187. When accumulation of misfolded proteins overwhelms ERAD, autophagy is induced as a secondary response to limit protein build‐up 187, 191. Autophagy is a pathway involved in the degradation of bulk components such as cellular macromolecules and organelles. It involves target recognition and selectivity, sequestering targets within autophagosomes, followed by the fusion of the autophagosome with the lysosome, where targets are then degraded by lysosomal hydrolases 187, 192. The direct link between ER stress and autophagy has been established in both *Saccharomyces cerevisiae* and mammalian cells, where autophagy plays a solely cytoprotective role. The PERK (eIF2α) and IRE1 (TRAF2/JNK) branches of the UPR have been implicated in ER stress‐induced autophagy in mammalian systems to avoid accumulation of lethal disease‐associated protein variants 192. IRE1–JNK signalling activates Beclin 1, a key player and regulator of autophagy, via the phosphorylation of Bcl‐2 and the subsequent dissociation from Beclin 1. This then leads to the activation of ATG proteins required for the formation of the autophagolysosome 193. Overall, these mechanisms decrease the build‐up of improperly folded proteins in the ER thus allowing adaptive and repair mechanisms to re‐establish homeostasis. As the amounts of improperly folded proteins decrease, the UPR switches off. However, the molecular details of UPR attenuation still remain to be further elucidated.

Overall, the three mechanisms describe above decrease the build‐up of proteins in the ER which allows adaptive and repair mechanisms to re‐establish homeostasis. As the amounts of improperly folded proteins decrease, the UPR switches off. However, the molecular details of UPR attenuation remain to be further elucidated.

#### Regulation of MAMs

Mitochondria‐associated membranes (MAMs), which are mainly responsible for Ca^2+^ homeostasis maintenance as well as lipid transport, mediate the interaction between the ER and mitochondria thereby controlling mitochondrial metabolism and apoptosis 194. MAMs contain many proteins and transporters which mediate mitochondrial clustering and fusion, such as the dynamin‐like GTPase mitofusin‐2 (MFN2) 195. MFN2 interacts with PERK, serving as an upstream modulator and thereby regulating mitochondrial morphology and function as well as the induction of apoptosis 196. Furthermore, the cytosolic domain of PERK serves as an ER‐mitochondria tether, thus facilitating ROS‐induced cell death 197.The sigma 1 receptor (Sig‐1R) is located in the MAMs and forms a complex with BiP. Recent studies show that S1R stabilizes IRE1 at the MAMs upon ER stress, promoting its dimerization and conformational change, and prolonging the activation of the IRE1–XBP1 signalling pathway through its long‐lasting endoribonuclease activity. Furthermore, mitochondria‐derived ROS stimulates IRE1 activation at MAMs 198. Another MAM component is Bax‐inhibitor‐1 (BI‐1), regulating mitochondrial Ca^2+^ uptake and apoptosis. BI‐1 is a negative regulator of IRE1‐XBP1 signalling and in BI‐1 deficient cells there is IRE1 hyperactivation and increased levels of its downstream targets 199. Apoptosis activation by the UPR results in mitochondrial membrane permeabilization, with the resulting Ca^2+^ transfer potentially triggering mitochondrial cytochrome c release 200. Less well understood are the interactions of the mitochondria with the ER during sublethal ER stress. The latter results in more ER‐mitochondria contacts than lethal levels of ER stress, allowing for transfer of Ca^2+^ and enhancement of ATP production through increased mitochondrial metabolism 201 (Fig. 1). These evidences demonstrate the importance of the ER‐mitochondria communication in regulating the ER homeostasis and in coordinating the cellular response to ER stress, thereby restoring cellular homeostatic condition or leading towards cell death.

#### Redox homeostasis

Oxidative stress can be induced through several mechanisms and is critically controlled by the UPR. PERK activity helps to maintain redox homeostasis through phosphorylation of NRF2 which functions as a transcription factor for the antioxidant response 202. ATF4 also regulates redox control and has been shown to protect fibroblasts and hepatocytes from oxidative stress 173, as well as ensuring that there is an adequate supply of amino acids for protein and GSH biosynthesis 203. However, in neurons and HEK293 cells ATF4 was shown to induce cell death in response to oxidative stress while CHOP was reported to induce ERO1‐α, resulting in ER Ca^2+^ release and apoptosis in macrophages 204. Direct interactions of PDIs with ER stress sensors, protein S‐nitrosylation and ER Ca^2+^ efflux that is promoted by ROS contribute to redox homeostasis and by extension to the balance between prosurvival and prodeath UPR signalling 205. As such, these signalling loops are paramount to normal cellular function.

#### Global metabolic impact of the UPR

It was recently shown that the UPR and mitochondrial proteotoxic stress signalling pathways converge on ATF4 to induce the expression of cytoprotective genes 174. Another pathway regulating energy metabolism is the nutrient‐sensing mammalian target of rapamycin (mTOR) signalling hub. mTOR is associated with the UPR through crosstalk with regulatory pathways (reviewed in 206), and mTOR inhibitors such as rapamycin lead to the activation of PERK signalling, thus favouring cell viability 207. PERK can also regulate the PI3K–AKT–mTORC1 axis through the activation of AKT. Furthermore, it was observed that mTORC2 plays a role in the inhibition of PERK through AKT activation 208. Altogether these data suggest that crosstalk between mTOR and the UPR is complex and occurs through multiple pathways.

##### Lipid metabolism

The UPR can also be activated by deregulated lipid metabolism. In this regard, the UPR has been shown to be activated in cholesterol‐loaded macrophages resulting in increased CHOP signalling and apoptosis 209. Notably, chronic ER stress leads to insulin resistance and diabetes in obesity. This is caused by alterations in lipid composition which lead to inhibition of SERCA activity and hence ER stress 210. On the other hand, the UPR is involved in systemic metabolic regulation. Disturbance of ER homeostasis in the liver is involved in hepatic inflammation, steatosis and nonalcoholic fatty liver disease 211. The PERK–eIF2α pathway has been reported to regulate lipogenesis and hepatic steatosis. Compromising eIF2a phosphorylation in mice by overexpression of GADD34 results in reduced hepatosteatosis upon high‐fat diet 212. ATF4 the downstream effector of PERK–eIF2α pathway has also been suggested to regulate lipid metabolism in hepatocytes in response to nutritional stimuli by regulating expression of genes involved in fatty acid and lipid production 213, 214. Furthermore, it has been demonstrated that the IRE1–XBP1–PDI axis links ER homeostasis with VLDL production which plays an important role in dyslipidaemia 215. In addition, XBP1 is required for the normal hepatic fatty acid synthesis and it was shown that selective XBP1 deletion in mice resulted in marked hypocholesterolaemia and hypotriglyceridaemia 216. These studies suggest that ER stress and the UPR are involved in lipid metabolism. Relieving ER stress ameliorates the disease state associated with lipid metabolism alterations, suggesting that targeting ER stress might serve as a therapeutic strategy for treating diseases associated with lipid accumulation.

##### Glucose metabolism

It has been suggested that in the liver the PERK–eIF2α pathway is responsible for disruption of insulin signalling caused by intermittent hypoxia, though IRE1–JNK pathways may still play a role 217. Adiponectin is widely regarded as a marker of functional glucose metabolism and as a suppressor of metabolic dysfunctions. In hypoxic and ER‐stressed adipocytes, reduced adiponectin mRNA levels are observed due to negative regulation by CHOP 218, 219. In β‐cells, it was shown that IRE1 is involved in insulin biosynthesis after transient high glucose levels. However, chronic exposure to high glucose leads to full UPR induction and insulin downregulation^220^. IRE1 signalling was shown to be involved in insulin resistance and obesity through JNK activation. In hepatocytes, IRE1‐dependent JNK activation leads (a) to insulin receptor substrate 1 (IRS1) tyrosine phosphorylation (pY896) decrease and (b) to AKT activation leading to an increase of IRS1 phosphorylation (pS307), consequently blocking insulin signalling. A role for XBP1 in the pancreas was demonstrated by the fact that β‐cell‐specific XBP1 mutant mice show hyperglycaemia and glucose intolerance due to decreased insulin release of β‐cells 221. ER stress‐induced activation of ATF6 in rat pancreatic beta cells exposed to high glucose, impairs insulin gene expression and glucose‐stimulated insulin secretion. Interestingly, knocking down expression of orphan nuclear receptor short heterodimer partner (SHP) previously reported to be involved in beta cell dysfunction by downregulating expression of PDX‐1 and RIPE3b1/MafA partly mitigated this effect. However, it remains unclear how ATF6 induces expression of SHP and whether ATF6 alone can directly regulate the expression of insulin, PDX‐1 and RIPE3b1/MafA 222. It has been suggested that physiological impact of ER stress with respect to glucose metabolism depends upon the availability of glucose. Indeed acute glucose availability in beta cells leads to concerted efforts of each branch of UPR to supply insulin, while chronic glucose stimulation leads to depletion of insulin production and beta cell mass due to apoptosis. Moreover, chronic fasting conditions in mice have shown that XBP1s directly activates the promoter of the master regulator of starvation response, PPARα demonstrating a further link between the UPR and glucose and lipid metabolism 223. Acquiring further knowledge on link between UPR and metabolic sensor mechanisms will significantly expand the possibility of gaining beneficial metabolic output. Taken together this indicates that the UPR arms are critical for the cell to regulate metabolism through regulating mTOR signalling, lipid homeostasis as well as insulin signalling.

### Downstream impact of UPR activation

The activation of UPR leads to the modulation of many cellular pathways, thereby influencing prosurvival mechanisms as well as processes such as proliferation, differentiation, metabolism and cell death.

#### UPR‐associated cell death

Following prolonged activation of the UPR, the cellular response switches from prosurvival to prodeath. Several types of cell death, including apoptosis, necrosis/necroptosis and autophagic cell death, can be induced following ER stress.

##### Apoptosis

Unresolved ER stress can lead to the activation of either the intrinsic (mitochondrial) or extrinsic [death receptor (DR)] pathways of apoptosis. Both pathways trigger activation of caspase proteases that dismantle the cell, and all of the three branches of the UPR are involved in apoptosis. In the extrinsic pathway, the activation of DRs on the PM leads to the recruitment of caspases to the DRs and their proximity‐induced trans‐autoactivation. Intrinsic apoptosis involves the release of cytochrome *c* (along with other proapoptotic factors) from the mitochondria, which promotes the formation of a cytosolic protein complex to activate a caspase cascade. This release is controlled by pro‐ and antiapoptotic members of the BCL‐2 protein family. In particular, the BH3‐only members of the family including PUMA, NOXA and BIM are pivotal components of ER stress‐induced apoptosis 224, and cells deficient in BH3‐only proteins are protected against ER stress‐induced cell death 190. ER stress leads to transcriptional upregulation of these proapoptotic molecules resulting in cytochrome *c* release. Both the IRE1 and PERK arms of the UPR have been linked to induction of apoptosis during ER stress. In particular, CHOP, a transcription factor that is downstream of PERK, and a direct target of ATF4, has been implicated in the regulation of apoptosis during ER stress. As discussed in section PERK signalling CHOP‐induced expression of GADD34 promotes dephosphorylation of p‐eIF2α reversing translational inhibition and allowing transcription of genes including apoptosis‐related genes 172. CHOP activates transcription of BIM and PUMA, while it represses transcription of certain antiapoptotic BCL‐2 family members such as MCL‐1 225. In addition, the ATF4/CHOP pathway can increase the expression of other proapoptotic genes, such as TRAIL‐R1/DR4 and TRAIL‐R2/DR5 which promote extrinsic apoptosis 180. Apart from CHOP, p53 is also involved in the direct transcriptional upregulation of BH3‑ only proteins during ER stress. However, the link between p53 activation and ER stress is unclear 226.

Although IRE1–XBP1s signalling is mainly prosurvival, IRE1 can promote apoptosis. Activated IRE1 can interact directly with TRAF2, leading to the activation of apoptosis signal‐regulating kinase 1 (ASK1) and its downstream targets c‐Jun NH2‐terminal kinase (JNK) and p38 MAPK 227, 228. Phosphorylation by JNK has been reported to regulate several BCL‐2 family members, including the activation of proapoptotic BID and BIM, and inhibition of antiapoptotic BCL‐2, BCL‐XL and MCL‐1 229, 230. In addition, p38 MAPK phosphorylates and activates CHOP, which increases expression of BIM and DR5, thereby promoting apoptosis 231, 232. In fact, cell death induction in HeLa cells overexpressing CHOP is dependent on its phosphorylation by p38 MAPK 233. Interestingly, it was proposed that ER stress and MAPK signalling act in a positive feed‐forward relationship, as ER stress induces MAPK signalling which in turn increases ER stress 234. IRE1 signalling may also contribute to apoptosis induction through prolonged RIDD activity which degrades the mRNA of protein folding mediators 142.

Interestingly, recent studies indicate a role for miRNAs in the induction of apoptosis following prolonged ER stress. For example, miRNA29a which is induced during ER stress via ATF4 results in the downregulation of antiapoptotic Bcl‐2 family protein Mcl‐1, and thus promotes apoptosis 235. miRNA7 has also been linked with ER stress‐induced apoptosis, where IRE1 reduces miRNA7 levels which results in the stability of a membrane‐spanning RING finger protein, RNF183. RNF183 has an E3 ligase domain that then causes the ubiquitination and subsequent degradation of the antiapoptotic member of the BCL‐2 family BCL‐XL. Following prolonged ER stress, increased expression of RNF183 via IRE1 leads to increased apoptosis 236.

In the last decade, it also became clear that ER stress can profoundly modify the immunological consequences of apoptotic cell death. Accumulating *in vitro* and *in vivo* evidence have highlighted that the activation of the PERK arm of ER stress evoked in response to selected of anticancer therapies (including anthracyclines, oxaliplatin, radiation and photodynamic therapy (reviewed in 237), drives a danger signalling module resulting in the surface exposure of the ER luminal chaperone calreticulin and the exodus of other danger‐associated molecular patterns, eliciting immunogenic cell death (reviewed in 238).

##### Necroptosis

Necroptosis, a programmed form of cell death, is dependent on the activation of receptor‐interacting protein kinase 1 (RIPK1), RIPK3 and mixed lineage kinase domain‐like (MLKL) protein and has been linked to ER stress. In an *in vivo* mouse model of spinal cord injury, there is induction of necroptosis and ER stress, with localization of MLKL and RIPK3 on the ER in necroptotic microglia/macrophages suggesting a link between necroptosis and ER stress in these cells 239. Necroptosis is frequently activated downstream of TNFR1 when apoptosis is blocked 240. This has been linked to ER stress‐induced necroptosis whereby tunicamycin kills L929 murine fibrosarcoma cells by caspase‐independent, death ligand‐independent, TNFR1‐mediated necroptosis 241.

#### Autophagic cell death

Endoplasmic reticulum stress has also been connected to autophagic cell death. Autophagy not only promotes cell survival, but can also mediate nonapoptotic cell death under experimental conditions when apoptosis is blocked, or in response to treatments that specifically trigger caspase‐independent autophagic cell death 192. IRE1α mediated TRAF2 and ASK1 recruitment, and subsequent JNK activation mediates autophagy. JNK‐mediated phosphorylation of BCL‐2 releases Beclin‐1 (while XBP1s also transcriptionally upregulates its expression), which interacts with the ULK1 complex to promote vesicle nucleation that leads to the formation of the autophagosome 242. Activated PERK can induce autophagy through ATF4 by inducing vesicle elongation while Ca^2+^ release from the ER lumen through the IP3R can relieve mTOR inhibition on the ULK1 complex 187.

#### UPR‐associated morphological changes

Endoplasmic reticulum stress causes morphological changes in cellular models. Experiments to date have largely focused on the morphologies associated with apoptotic and autophagic cell death resulting from UPR activation. UPR‐regulated flattening and rounding of cells, indicative of cell death, has been observed in many model systems, with traditional caspase‐dependent apoptosis being responsible 200, 243, 244, 245, 246, 247, 248. These morphological changes can be reversed by physiological and pharmacological ER stress relief 247, 249. Both IRE1 and PERK arms of the UPR have been implicated in the observed changes 193, 243, 244, 247, 249, 250, 251. As described above, programmed cell death and its associated morphological changes have become a focal and much researched outcome of the use of UPR‐inducing cytotoxic agents.

An intensively studied consequence of ER stress is the epithelial to mesenchymal transition (EMT) and its role in cancer invasion and metastasis. EMT is an essential component of tissue repair following wounding, allowing for the migration of new healthy cells into any lesions that have occurred. Morphological changes indicative of EMT have been observed in multiple cell models under physiologically relevant stress (e.g. hypoxia) and pharmacological induction of ER stress 252, 253, 254, 255. The IRE1–XBP1 pathway has been reported to negatively regulate the traditional epithelial marker E‐cadherin, while positively regulating the mesenchymal marker *N*‐cadherin in models of colorectal, breast and pulmonary fibrosis 254, 256, 257. Breast cancer and pulmonary fibrosis models showed an IRE1–XBP1‐dependent regulation of mesenchymal promoting transcription factor SNAIL that is responsible for EMT 254, 256. Human mammary epithelial cells undergo EMT in response to PERK activation, and PERK‐mediated phosphorylation of eIF2α is required for invasion and metastasis 258. Other ER stress‐regulated pathways have been proposed to act in the EMT in cellular models, including autophagy and activation of c‐SRC kinase in tubular epithelial cells 259 and the compensatory activation of the NRF‐2/HO‐1 antioxidative stress response pathway in HT‐29 and DLD‐1 colon cancer cells 252. Therefore, UPR signalling pathways appear to induce morphological changes indicative of EMT. These data have generated interest in the field of cancer research where the pharmacological inhibition of UPR components might be used to reduce tumour invasiveness and metastasis.

#### Hormone production

The tissues and cells of the endocrine system responsible for hormone production and extracellular signalling often have a high protein load, resulting in ER stress and activation of the UPR. OASIS (CREB3L1) and ATF6α have been shown to regulate arginine vasopressin (AVP), a potent vasoconstrictor, in murine and rat models 260, 261. Upon dehydration or salt loading in rat models, cleaved active OASIS is observed binding the AVP promoter region, directly upregulating protein expression 260. In ATF6^−/−^ murine models subjected to intermittent water deprivation, similar downstream effects were observed, but signalling pathways were not investigated 261. ER stress‐inducing agents palmitate and oxysterol 27‐hydroxycholesterol both result in a reduction in leptin (a long‐term mediator of energy balance) expression and extracellular concentrations. This has been attributed, by using ChIP analysis and siRNA knockdowns, to the fact that the PERK downstream target CHOP negatively regulates C/EBPα, transcriptionally downregulating its translation and release 262, 263. UPR activation has been implicated in the hypothalamic and brown adipose tissue response to thyroid hormone triiodothyronine (T3). Elevated T3 levels induce the UPR downstream of AMPK in the ventromedial nucleus of the hypothalamus, resulting in decreased ceramide levels. JNK1 KO revealed that it acts downstream of this AMPK‐dependent activation, possibly as a target of IRE1 but to our knowledge no studies have yet confirmed this 264. In response to ER stress in hepatocytes, CREBH is exported from the ER and cleaved in the Golgi apparatus. The CREBH cytosolic fragment binds to the promoter region of hepcidin and transcriptionally upregulates its production 265. These examples of UPR‐regulated hormone production and release give scope for further investigation into the longer term, system wide effects of UPR signalling outside of the current focuses on cytotoxicity and acute diseases.

## Physiological ER stress signalling

It has been established that ER stress signalling is important in interorganelle and intercellular interactions. It therefore comes as no surprise that it forms a significant network of interactions upon which normal physiology is based. This is not only the case in humans, but is also conserved throughout species and has been an important fact in the design of experimental model organisms to further study ER stress signalling and it role in physiology and disease.

### Embryology and development

The UPR as the major conduit of ER stress regulation has been extensively studied in developmental biology in the majority of organisms commonly used in translational research. The use of multiple models has been important in discerning the variable ER stress signalling between species, as demonstrated by the discovery that protein quality control in mammals is critically dependent on ATF6 while the major player in *Caenorhabditis elegans and Drosophila melanogaster* is IRE1 182, 266. Mammalian and other embryos implanted *in vitro* or naturally, undergo a multitude of physical, biochemical and cellular stresses involving epigenetic changes as well as a disproportional increase in protein synthesis load that affect cell differentiation, proliferation and growth.^267^. In zebrafish, transgenic models have been generated to monitor XBP1 splicing during development and implantation, showing that maternal XBP1s is active in oocytes, fertilized eggs and early stage embryos, presenting a potential model for study of the impact of water pollutants on embryogenesis 268. It was recently shown that in medaka fish the JNK and RIDD pathways are dispensable for growth, with development solely dependent on the XBP1 arm of IRE1 signalling, thereby supporting the hypothesis that XBP1 and RIDD may be differentially utilized in development and homeostasis 269. In *C. elegans* it has been postulated that the IRE1‐XBP1 axis as well as the PERK pathway are responsible for the maintenance of cellular homeostasis during larval development 270. Pronephros formation was shown to be BiP dependent in *Xenopus* embryos, where BiP morpholino knockdown not only blocked pronephros formation but also attenuated retinoic acid signalling, impacting markers such as the Lim homeobox protein 271. In early mouse development, it was shown that the BiP promoter is activated in both the trophoectoderm and inner cell mass at embryonic day 3.5 and that absence of BiP leads to proliferative defects and inner cell mass apoptosis, suggesting it is necessary for embryonic cell growth and pluripotent cell survival 272. Furthermore, mouse studies revealed that ER stress proteins such as BiP, GRP94, calreticulin and PDIA3 were downregulated in adult neural tissues compared to embryonic ones, suggesting a pivotal role for ER stress signalling in the development of neural tissues such as the brain and retina 273. Beyond the nervous system, ER stress signalling impairment has repeatedly shown mouse embryonic lethality and, in particular in the hepatocellular system, multiple studies have demonstrated that IRE1 and XBP1 signalling defects lead to fetal liver hypoplasia, intrauterine anaemia and early antenatal pancreatic dysfunction 274. The UPR is intrinsically linked to the mouse embryonic morula–blastocyst transition 275 and this, in combination with evidence that there is an immediate postnatal downregulation of BiP, shows that there is an important role for the UPR both in early and late gestation 276. Taking all this evidence into consideration, it is apparent that the correct integration of signals both intracellularly and between the developing oocyte, follicular environment and supporting cumulus cells is absolutely essential for embryonic development, making ER stress signalling a key regulator in the earliest stages of life in all organisms 277.

### Growth and differentiation

Many cell types experience a high protein load during various stages of differentiation and maturation, resulting in ER stress. In several cases, morphological changes required for the final function of the cell would not be possible without transient activation of the UPR's cytoprotective mechanisms. Deletion of PERK in murine models results in loss of pancreatic β cell architecture but not in cell death, and was accompanied by an increase in β cell proliferation. This morphological change results in a diabetes mellitus‐like pathology and is not a result of increased cell death as previously proposed 278. Various haematopoietic lineages require the activation of the UPR in order to survive ER stress resulting from production of immunoglobulins and lysosomal compartments in order to reach maturity 279, 280, 281. One physiological function that is indispensable for survival is the innate immune response, and cell differentiation is at its epicentre. The conversion of B lymphocytes to highly secretory plasma cells is accompanied by a huge expansion of the ER compartment, and genetic alterations to induce immunoglobulin production are good examples of the necessity of ER signalling in normal physiology 123. This is supported by a study that suggests the UPR, and the PERK pathway in particular, govern the integrity of the haematopoietic stem‐cell pool during stress to prevent loss of function 282. The ability of skin fibroblasts to produce collagens and matrix metalloproteinases (proteins increased at wound sites), along with their ability to differentiate into myofibroblasts, provides another example where physiological ER stress may drive morphological cellular transition 283. Although not yet fully characterized, the RIDD pathway has been linked to a multitude of physiological processes including lysosomal degradation and xenobiotic metabolism through cytochrome P450 regulation 284. At the same time, substrates of regulated intramembrane proteolysis such as CREBH are involved in normal physiological processes such as gluconeogenesis 284. Another substrate of regulated intramembrane proteolysis, OASIS, is involved in multiple stages of bone homeostasis and development. Mice lacking OASIS present with severe osteopenia, which is compounded by the fact that the gene for type 1 collagen is an OASIS target 285. Moreover, osteoblast OASIS expression is controlled by factors essential to osteogenesis (BMP2), pointing to a PERK‐eIF2α‐ATF4 pathway upregulation during osteoblast differentiation, where ATF4 restores deficiencies of PERK null osteoblasts all the while impacting apoptosis for bone remodelling 251, 286. Furthermore, a link between osteoblast differentiation and hypoxia has been established, with decreased vascularization shown in OASIS null mice pointing towards a potential role of ER stress in angiogenesis during bone development 287. This signalling cascade does not only restrict itself to the normal physiology of bone but also modulates UPR signalling in astrocytes and is responsible for the terminal, early to mature, goblet cell differentiation in the large intestine 288, 289, 290.

### Metabolism

The ER is a site of significant metabolic regulation. The UPR plays a major role in the regulation of glycolysis and it was recently shown that IRE1 mediates a metabolic decrease upon glucose shortage in neurons, suggesting an important role for the UPR as an adaptive response mechanism in relation to energy metabolism 291. Moreover, mTOR signalling adjusts global protein synthesis, which is a highly energy consuming process, and thereby regulates energy metabolism (reviewed in 292).

#### Lipid homeostasis

The ER is heavily involved in lipid homeostasis. Characteristically, hepatocytes are enriched in SER, because in addition to protein synthesis, these cells also synthesize bile acids, cholesterol and phospholipids. XBP1 ablation in murine liver results in hypolipidaemia due to feedback activation of IRE1 caused by the lack of XBP1. Activated IRE1 induces the degradation of mRNAs of a cohort of lipid metabolism genes via RIDD, demonstrating the critical role of IRE1–XBP1 signalling in lipid metabolism and suggesting that targeting XBP1 may be a viable approach to the treatment of dyslipidaemias 113. It was also reported that in hepatocyte‐specific IRE1‐null mice, XBP1 is involved in very low‐density lipoprotein synthesis and secretion 215. Interestingly, ATF6 has also been shown to have a role in adipogenesis by inducing adipogenic genes and lipid accumulation 293.

#### Glucose metabolism

The UPR is also involved in regulating glucose metabolism. Initial murine studies suggested the PERK–eIF2α arm was responsible for impaired insulin signalling due to knock out effects on beta cells during development. Further studies have since shown that IRE1 RIDD activity is responsible for a reduction in the mRNA of proinsulin processing proteins, including *INS1*,* PC1* and *SYP*. These effects can be observed in cases of XBP1 deficiency and in cases of extensive UPR activation, highlighting the divergent effects of IRE1 RNase activity 119, 221, 294.

#### Amino acid metabolism

The UPR is also described to be involved in amino acid metabolism. It was recently described that ATF4 mediates increased amino acid uptake upon glutamine deprivation 295. Furthermore, a low protein diet leads to the upregulation of cytokines mediated by IRE1 and RIG1 which results in an anticancer immune response in tumours 296. In summary, these findings show the importance of the various UPR arms in cell metabolism and energy homeostasis with effects not only on the cell itself but also on the whole cellular environment.

## Pharmacological targeting of the UPR

Several small molecules have been reported to modulate (activate or inhibit) one or more arms of the UPR. Importantly, these molecules have shown promising beneficial effects in diverse human diseases (Table 1). X‐ray cocrystal structures are now available for IRE1 and PERK with several endogenous or exogenous ligands. The understanding of how small molecules bind to the active sites and modulate the function of IRE1 and PERK will have a profound impact on the structure‐based drug discovery of novel UPR modulators. Available X‐ray structures, in addition to mutagenesis analysis of critical amino acids 297, have revealed a variety of unexpected allosteric binding sites on IRE1 297, 298, 299.

**Table 1:** Different modulators that target the UPR‐transducer protein pathways. Molecule name, respective molecular target and brief description with the associated reference are provided (ND: not determined)

UPR Arm	Name	Target	Brief description	Reference
PERK	GSK2656157	PERK Kinase	In preclinical stage for multiple myeloma and pancreatic cancer	314, 364
	Salubrinal	GADD34/PP1c	Inhibition of eIF2α dephosphorylation	365, 366, 367
			In ALS, it increases lifespan of mutant superoxide dismutase 1 transgenic mice	
			In Parkinson's disease, it increases neuronal survival of α‐synuclein transgenic mice	
	ISRIB	eIF2β	Decreased ATF4 expression	322
	Guanabenz	GADD34/PP1c	Inhibitor of eIF2α phosphatase,	368
	Sephin1	GADD34 (PP1c)	Inhibitor of eIF2α phosphatase	369
IRE1	Salicylaldimines	IRE1 RNase	IRE1αRNase active‐site inhibitor	305
	STF‐083010	IRE1 RNase	IRE1α RNase active‐site inhibitor	308
			In preclinical stage for multiple myeloma treatment	
	MKC‐3946	IRE1 RNase	IRE1α RNase active‐site inhibitor	307, 370
			In preclinical stage for multiple myeloma treatment	
	4μ8c	IRE1 RNase	IRE1α RNase active‐site inhibitor	306
			In preclinical stage for multiple myeloma treatment	
	APY29	IRE1 Kinase	IRE1α kinase active‐site inhibitor	303
	Sunitinib	IRE1 Kinase	IRE1α kinase active‐site inhibitor	85, 304
			FDA approved for renal cell carcinoma	
			It acts on multiple kinases	
	KIRA	IRE1 Kinase	IRE1α kinase active‐site inhibitor	371
	Toyocamycin	IRE1 RNase	IRE1α RNase active‐site inhibitor	309, 372
			In preclinical stage for various cancers treatment	
	3‐ethoxy‐5,6‐dibromosalicylal‐ dehyde	IRE1 RNase	IRE1α RNase active site inhibitor	305
	Apigenin	Proteasome	Increase of IRE1a nuclease activity in model	373
	FIRE peptide	IRE1 Kinase	Modulation IRE1 oligomerization *in vitro*,	85
			Xbp1 mRNA cleavage *in vitro*, in cell culture and *in vivo* (*Caenorhabditis elegans*)	
ATF6	Apigenin	ATF6	Upregulation of ATF6 expression	373
	Baicalein	ATF6	Upregulation of ATF6 expression	374
	Ceapin	ND	Inhibitor of ATF6	323
	Kaempferol	ATF6	Downregulation of ATF6 expression	375
	Melatonin	ATF6	Inhibitor of ATF6	325
	Compound 147	ATF6	Activator of ATF6	376
	Compound 263	ATF6	Activator of ATF6	376
	16F16	PDI	Inhibitor of PDI	377

### Pharmacological modulators of IRE1

IRE1 signalling information along with CHOP/Gal4‐Luc cells and UPRE‐Luc engineered cells were used to screen large chemical libraries in high throughput screening assays for discovery of pathway‐selective modulators of IRE1 300.

#### IRE1 ATP‐binding site

IRE1 modulators have been discovered primarily by traditional drug discovery methods, identifying inhibitors specific to the kinase or RNase domain (Table 1). The IRE1 kinase modulators were used as tools to understand the allosteric relationship between the kinase and RNase domains 301, 302. Kinase inhibitors can be broadly classed as (a) ATP‐competitive inhibitors that inhibit the kinase domain and activate the RNase domain and (b) ATP‐competitive inhibitors that inhibit the kinase domain and inactivate RNase (kinase inhibiting RNase attenuators – KIRAs). Available IRE1 crystal structures reveal a possible mechanism of RNase activation by conformational changes that occur in the kinase domain when transitioning from a monomeric to an active dimeric state. Type I IRE1 kinase inhibitors include APY29 303 and sunitinib 304, which target the ATP‐binding site and inhibit the phosphorylation but stabilize the active form of the kinase domain. An active kinase conformation is seen in human *apo* dP‐IRE1* (PDB 5HGI), as a back‐to‐back dimer. Notably, the DFG motif (Asp711‐Phe712‐Gly713) faces into the active site (DFG‐in), with helix‐αC‐in conformation. In contrast, human IRE1 bound to KIRA compound 33 (PDB: 4U6R) shows an inactive kinase conformation, with DFG‐in and helix‐αC‐out conformation. The inactive conformation is incompatible with back‐to‐back dimer formation due to the displaced helix‐αC 301. Imidazopyrazine‐based inhibitors and other KIRAs allosterically inhibit the RNase activity of phosphorylated IRE1 by possibly displacing helix‐αC from an active conformation to an inactive conformation 301.

#### IRE1 RNase‐binding site

IRE1 RNase inhibitors include salicylaldehydes 305 4μ8C 306, MKC‐946 307, STF‐83010 308, toyocamycin 309 and hydroxyl‐aryl‐aldehydes 86. The reported cocrystal structures of murine IRE1α with salicyaldehyde‐based inhibitor show that Lys 907 is involved in Schiff base arrangement (PDB code: 4PL3 86). Lys 907 is a crucial residue present within the hydrophobic pocket of the IRE1 RNase catalytic site 310. Quercetin is reported to activate IRE1 through a site distinct from the nucleotide‐binding site (crystal structure PDB 3LJ0), increasing the population of IRE1 dimers *in vitro* 299. A recent *in silico* study identified the anthracycline antibiotic doxorubicin as an inhibitor of the IRE1‐XBP1 axis 311. Covalent binders are very efficient in the sense that they completely block the proteins to which they bind, but this can also have several drawbacks 312. Noncovalent kinase and allosteric modulators in general inhibit competitively and are thus less efficient, but can at the same time be extremely useful in obtaining new insights for developing selective and potent modulators of IRE1α‐XBP1 signalling (Table 1).

#### Other IRE1 modulators

Peptides derived from the kinase domain of human IRE1 promote oligomerization *in vitro*, enhancing XBP1 mRNA cleavage activity *in vitro* and *in vivo* 85. However, although peptide‐based modulators have limited clinical application 313 (Table 1) peptide mimetics may prove more useful. These are different aspects that can be exploited to develop selective IRE1 modulators. Despite significant progress in understanding IRE1 signalling and in the development of modulators of IRE1 activity, several questions still remain to be answered to fully control IRE1 activity and signalling outcomes, including how to selectively target the XBP1 and RIDD arms of IRE1 signalling.

### Pharmacological modulators of PERK

Through biochemical screening of exclusive library collections and structure‐based lead optimization, GSK discovered PERK inhibitors GSK2606414 and GSK2656157 314. These potent PERK inhibitors can be orally administered 314, reducing tumour growth in mouse xenograft models 314, 315. GSK2606414 was also the first oral small molecule to prevent neurodegeneration *in vivo* in prion‐diseased mice, with GSK2606414 reducing the levels of p‐PERK and p‐eIF2α and restoring protein synthesis rates 316. Despite the promising selectivity profile, pharmacological inhibition of PERK in mice caused damage to exocrine cells and pancreatic beta cells, a similar phenotype to that observed in PERK^−/−^ mice 317. Furthermore, GSK2606414 and GSK2656157 were found recently to inhibit RIPK1 at nanomolar concentrations 318. To overcome the β‐cell toxicity, small molecules modulating the eIF2α pathway without directly inhibiting PERK were examined. Integrated stress response inhibitor (ISRIB) is the first small molecule described to bind and activate guanine nucleotide exchange factor eIF2B 319, 320. Unlike GSK inhibitors, ISRIB did not show any pancreatic toxicity 321. Interestingly, ISRIB increased learning and memory in WT mice 322 (Table 1).

### ATF6 modulators

The identification of small molecules that modulate ATF6 has been challenging due to lack of potentially druggable binding sites and unavailability of the protein crystal structure. Recently, Walter and colleagues identified selective inhibitors of ATF6 signalling, the small molecules Ceapins, using a high throughput cell‐based screen 323. Ceapins do not affect the IRE1 and PERK arms of the UPR. Ceapins are chemically classed as pyrazole amides and extensive biochemical and cell biology evidence show that they trap ATF6 in the ER and thus prevent its translocation to the Golgi upon stress 324. Ceapins sensitize cells to ER stress without affecting unstressed cells and hence have potential to be developed within the framework of a therapeutic strategy to induce cell death in cancer cells. A recent study identified melatonin as an ATF6 inhibitor, leading to enhanced liver cancer cell apoptosis through decreased COX‐2 expression 325. The activation of ATF6 depends on a redox process involving PDIs suggesting that PDI inhibitors such as PACMA31 326, RB‐11‐ca 327, P1 327 and 16F16 328 may be able to modulate ATF6 activation. Additionally, the serine protease inhibitor 4‐(2‐aminoethyl) benzenesulfonyl fluoride is reported to prevent ER stress‐induced cleavage of ATF6 329 (Table 1). Albeit the above developments hold strong promise for the future, very little is known to date about specific binding sites, which together with the lack of a crystal structure and insufficient templates to enable homology modelling, rational drug design targeting ATF6 remains a challenge. Availability of an ATF6 crystal structure is in this sense the key aspect, as this will provide atomistic level understanding of interactions and mechanism of action, and enable *in silico* based rational design of ATF6 modulators.

## The UPR in the clinic

**Figure 3:**
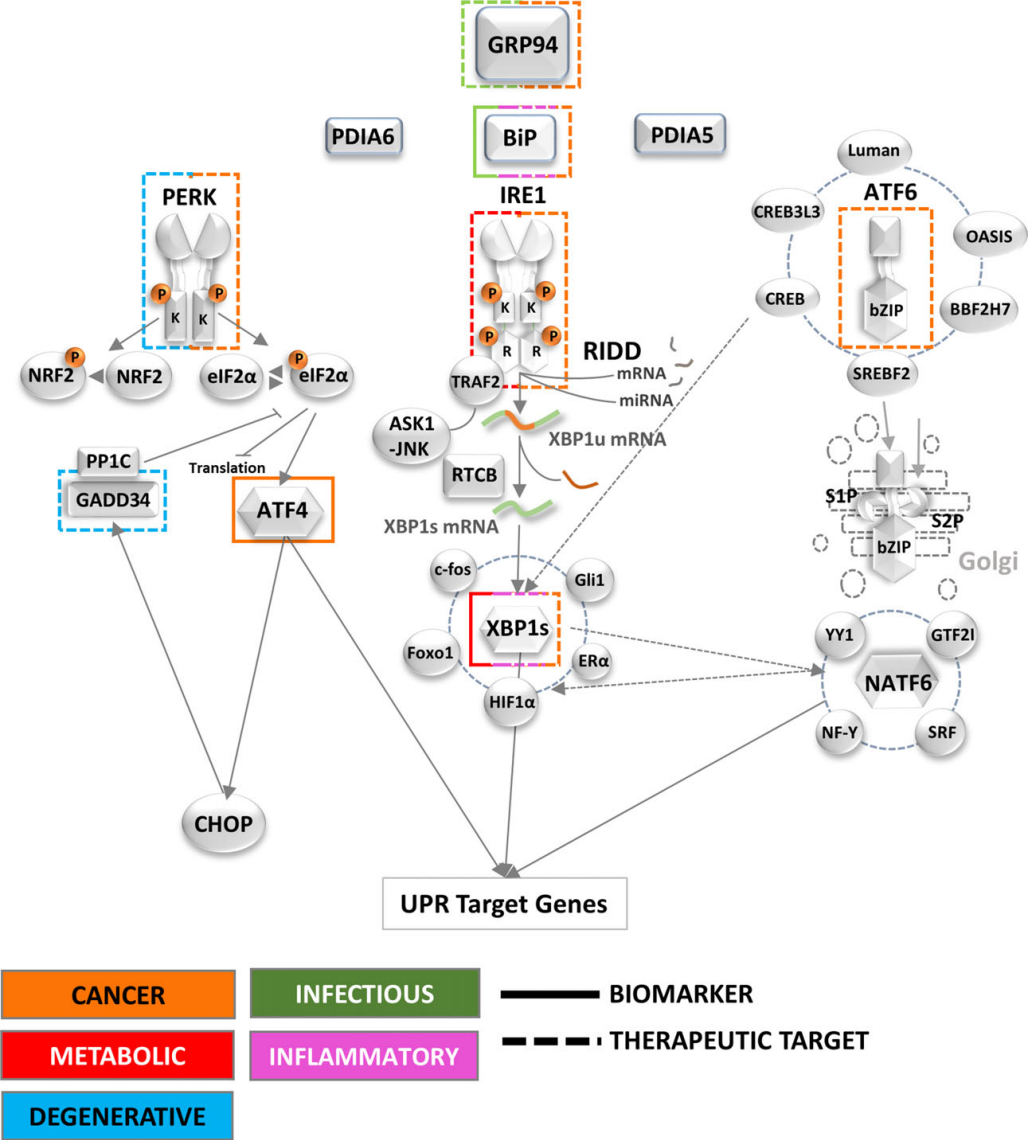
UPR disease biomarkers and therapeutic targets. Schematic representation of the UPR signalling pathway as defined in Fig. 2 and annotated with the relevance to disease of each component. The colour code indicates the type of disease (cancer: orange; metabolic disease: red; degenerative disease: blue; infectious disease: green; inflammatory disease: pink) and the lines indicate the role as biomarker (continuous line) or therapeutic target (dashed line).

In this section, we review recent preclinical and clinical studies in which UPR components were used as disease biomarkers or as therapeutic targets (Fig. 3). As already described in section Perturbing ER functions molecules have been designed to modulate ER stress by inducing the UPR (Brefeldin A, DTT), inhibiting SERCA Ca^2+^ ATPases (thapsigargin) or preventing the generation of glycoproteins, and hence, the induction of ER stress through calcium imbalance or misfolded protein accumulation. They were touted as potential antitumour therapies as they could potentially induce tumour cell death through ER stress overactivation. However, none of these compounds were used in the clinic due to their lack of specificity and high toxicity. It has been reported though that a prodrug analogue of thapsigargin, mipsagargin, did display acceptable tolerability and favourable pharmacokinetic profiles in patients with solid tumours 330. On the other hand, section 6 describes molecules that inhibit the various arms of the UPR.

### UPR biomarkers

Changes in UPR and ER stress markers in blood or tissue biopsy samples can be indicative of disease state and could be/are utilized as valuable biomarkers for different human pathologies. For instance, BiP has strong immunological reactivity when released into the extracellular environment 331, and in 1993, it was the first ER stress protein associated with the pathogenesis of osteogenesis imperfecta 332. Since then, further evidence suggests overexpression of BiP in several human diseases (reviewed in 333). The UPR transcription factors can also be seen as potential biomarkers of various diseases. ATF4 is upregulated and contributes to progression and metastasis in patients with oesophageal squamous cell carcinoma 334. Similarly, XBP1 overexpression is linked to progressive clinical stages and degree of tumour malignancy in osteosarcoma 335. In contrast, IRE1–XBP1 downregulation can differentiate germinal centre B cell‐like lymphoma from other diffuse large B‐cell lymphoma subtypes and contributes to tumour growth 336. Moreover, XBP1 is genetically linked to inflammatory bowel disease (IBD) 337. Using cohorts of IBD patients to test the association of 20 SNPs across the XBP1 gene region, it was found that three SNPs rs5997391, rs5762795 and rs35873774 are associated with disease, thus linking cell‐specific ER stress changes with the induction of organ‐specific inflammation. Quantitative changes in ER stress chaperones in the CSF have been proposed as possible biomarkers to monitor the progression of neurodegenerative diseases such as ALS 338, 339. Finally, the mesencephalic astrocyte‐derived neurotrophic factor (MANF) can be used as a urine biomarker for ER stress‐related kidney diseases 340. MANF localizes in the ER lumen and is secreted in response to ER stress in several cell types. Similarly, angiogenin was identified as an ER stress responsive biomarker found in the urine of patients with kidney damage 341. Thus, noninvasive ER stress‐related biomarkers can be used to stratify disease risk and disease development (Fig. 3).

### ER stress and UPR‐based therapies

Beyond their use as biomarkers, ER stress signalling components also represent relevant therapeutic targets. BiP was recently recognized as a universal therapeutic target for human diseases such as cancer and bacterial/viral infections 333. Antibodies targeting BiP exhibited antitumoural activity and enhanced radiation efficacy in non‐small‐cell lung cancer and glioblastoma multiforme in mouse xenograft models 342. It was also shown that short‐term systemic treatment with a monoclonal antibody against BiP suppressed AKT activation and increased apoptosis in mice with endometrial adenocarcinoma 343. Moreover, the ER‐resident GRP94 is being evaluated as a therapeutic target because of its ability to associate with cellular peptides irrespective of size or sequence 344. Preclinical studies have linked GRP94 expression to cancer progression in multiple myeloma, hepatocellular carcinoma, breast cancer and colon cancer. Finally, this protein has been identified as a strong modulator of the immune system that could be used in anticancer immunotherapy 345.

ER stress‐induced transcription factors can also represent relevant targets. Thus, XBP1s has been one of the main targets for drug discovery and gene therapy 346. Elimination of XBP1 improves hepatosteatosis, liver damage and hypercholesterolaemia in animal models. As such direct targeting of IRE1 or XBP1 can be a possible strategy to treat dyslipidaemias 113. In cancer, toyocamycin was shown to inhibit the constitutive activation of XBP1s expression in multiple myeloma cells as well as in patient primary samples 309. Despite being the least studied UPR arm, there are instances that ATF6 can be a specific clinical target. The activation of ATF6 but not IRE1 or PERK has been linked with airway remodelling in a mouse model of asthma 347. Additionally, these studies showed that expression of orosomucoid‐like 3 (ORMDL3) regulates ATF6 expression and airway remodelling through ATF6 target genes such as SERCA2b, TGFβ1, ADAM8 and MMP9 (Fig. 3, Table 2).

**Table 2:** ER stress‐centred clinical trials. A range of clinical entities in endocrinology, oncology and paediatrics have been targeted through clinical trials. This table presents such trials detailing the trial targeted, interventional agent investigated and national authority carrying out the investigation

Trial	Disease	Intervention	Country
Role of ER stress in the pathophysiology of type 2 diabetes	Diabetes mellitus, type 2	No intervention	France
ER stress and resistance to treatments in Ph‐negative myeloproliferative neoplasms	Polycythemia vera Essential thrombocythemia	Biological: RNA sample of total leucocytes before start of treatment	France
Effect of ER stress on metabolic function	Insulin resistance Diabetes Obesity	Drug: TUDCA	United States
		Other: placebo	
		Drug: sodium phenylbutyrate	
ER stress in chronic respiratory diseases	Chronic airway disorders Lung cancer	Observational	South Korea
TUDCA for protease‐inhibitor associated insulin resistance	HIV‐related insulin resistance Protease inhibitor‐related Insulin resistance	Drug: TUDCA	United States
		Other: placebo tablet	
ER stress in NAFLD	Obesity NAFLD	Drug: methyl‐D9‐choline	United States
TUDCA in new‐onset type 1 diabetes	Type 1 diabetes	Drug: TUDCA	United States
		Drug: Sugar Pill (placebo)	
Effects of Liraglutide on ER stress in obese patients with type 2 diabetes	Type 2 diabetes	Drug: liraglutide	United States
A clinical trial of dantrolene sodium in paediatric and adult patients with wolfram syndrome	Wolfram syndrome Diabetes mellitus optic nerve atrophy Ataxia	Drug: dantrolene sodium	United States

ER stress targets are also strong candidates for immunotherapy and vaccines development, a good example of which is the production of chaperone protein‐based cancer vaccines termed chaperone‐rich cell lysate (CRCL) 348. The CRCL are purified from tumour tissue or recombinantly produced and applied as vaccines against murine and canine cancers or infectious diseases. Advantages of CRCL vaccines include small quantities and easily obtained starting materials 349. Furthermore, DNA vaccination with gp96‐peptide fusion proteins showed increased resistance against the intracellular bacterial pathogen *Listeria monocytogenes* in a mouse model 350. To improve the efficacy of gp96 vaccines, gp96 was pooled with CpG in combination with anti‐B7H1 or anti–interleukin‐10 monoclonal antibodies to treat mice with large tumours 351. The heterogeneous or allogeneic gp96 vaccines protected mice from tumour challenge and re‐challenge. In addition to its role as a molecular chaperone, GRP94 was likewise identified as a peptide carrier for T‐cell immunization 352. However, the immunological application of GRP94 derived from its peptide binding capacity was not further investigated (Fig. 3, Table 2). The activation of ER stress has been reported as well in different critical care diseases models, such as sepsis 331, 332, liver, heart, brain and kidney ischaemia 353, 354, 355, 356, 357, 358, 359 and haemorrhagic shock 334, 335. But, the pathophysiological impact of ER stress activation in these conditions severely lacks characterization. Multiple factors such as inflammation, hypoxia present in sepsis and shock can induce ER stress but its effects are ambivalent. It has been shown that induction of ER stress is cytoprotective 353, 354, and that proteostasis promotors/disruptors such as 4‐PBA 336 or TUDCA 337 can be used to improve disease outcome. The increase of CHOP in renal tissue was reported to inhibit inflammatory response in and provide protection against kidney injury 336. Moreover, the activation of PERK seems to facilitate survival of lipopolysaccharide‐treated cardiomyocytes by promoting autophagy 338. Additionally, the activation of ATF6 before ischaemia reduced myocardial tissue damage during ischaemia/reperfusion (I/R) injury 339. Furthermore, induction of BiP in cardiomyocytes stimulated AKT signalling and protected against oxidative stress, conferring cellular I/R damage protection 340. In contrast, inhibition of ER stress was indicated to limit cellular damage in pathologies such as hepatic I/R 341. This contradiction may be due to interference between UPR and inflammatory pathways. CHOP‐/‐ mice were reported to have more prominent increase in NF‐kB activation and further upregulation of proinflammatory genes (CXCL‐1, MIP‐2, IL‐6) 342. Interestingly, inhibition of IRE1‐NF‐kB by resveratrol protected against sepsis‐induced kidney failure 343. In this light, the modulation of specific UPR branches is promising approach for therapy of critical care diseases.

As discussed above, understanding and characterizing the UPR has provided several potential targets to develop new therapeutics for various diseases, with an encouraging increase in the number of clinical trials based on ER stress pathway targets or associated drugs. Several of these trials [ClinicalTrials.gov, European Clinical Trials Database and the ISRCTN registry] have focused on diabetes mellitus. A trial testing TUDCA and 4‐PBA for the treatment of high lipid levels or insulin resistance was conducted by the Washington University School of Medicine; however, although this study was completed in 2014, the findings are not available yet. The results from the first completed human trial using BiP for rheumatoid arthritis are described in Box 1. We can anticipate that clinical trials to test ER stress targeting drugs in several other diseases will shortly ensue.

Box 1 **First‐in‐human trial**

**Intravenous infusion of GRP78/BiP is safe in patients**

In 2006, Brownlie *et al*. 362 reported that the prophylactic or therapeutic parenteral delivery of BiP ameliorates clinical and histological signs of inflammatory arthritis in mice. Ten years later, the first human clinical trial using intravenous BiP demonstrated that GRP78/BiP is safe in patients with active rheumatoid arthritis and some patients had clinical and biological improvements 363. In phase I/IIA RAGULA trial, 42 patients were screened, and 24 were randomized to receive either BiP or placebo. The study showed that after a single intravenous infusion, BiP may induce remission lasting up to 3 months in rheumatoid arthritis patients.

## Concluding remarks

The ER has evolved in our knowledge from a key player in proteostasis and the secretory pathway to a cornerstone of metabolic functions. Such wealth of information has allowed the identification of numerous mechanisms for fine‐tuning ER signalling, as well as motivated the need for their better characterization towards relevant health‐related applications. This drive to further ER knowledge has also led to the identification of emerging roles for the ER in physiology and disease. In particular, it appears an indispensable tool for cellular communication that reaches beyond the intracellular space. The concept of transmissible ER stress illustrates the far‐reaching control that ER signalling exerts in interorgan communication affecting disease pathogenesis and normal physiology 360, 361.

Our increasing knowledge of ER signalling mechanisms presents opportunities to exploit the resulting applications on multiple fronts, including bioengineering and health, concepts that may routinely overlap. For example, boosting ER protein production capacity may be applied to cell engineering to increase biologic therapy production. This will drive down costs of biologics, helping demand to be met and leading to more widely available medications, thus having a significant effect on public health. Population‐wide consequences of ER modulation may not be restricted to the production of biologic therapies as its applications could also contribute to bioengineering approaches for crop or livestock improvement.

A thorough understanding of the ER stress response and its role in physiology and pathophysiology can be applied to develop new ER stress targeted therapies and stratifying patients into cohorts suitable for ER‐targeted therapies. Considering the enormity of attrition rates of novel therapeutic discovery in an ever‐tightening financial climate, there is an urgent need for new therapeutic targets as well as precision tools that target and guide innovation to specific patient pools. ER stress signalling may provide such tools. Not only is it central to life itself but it is involved in a wide array of clinical presentations. Moreover, its effect on heterogeneous presentations within the same diseases makes it an attractive target for translational precision medicine. Of course, when undertaking medical research or trying to solve a biomolecular functional mystery one cannot look past the logistical aspect of the task ahead. The conserved metazoan nature of ER stress signalling combined with the emergence of high throughput and *in silico* strategies supplies researchers with a wealth of tools to study pathophysiology, from structure to function in multiple *in vivo* and *in vitro* models, producing robust results to be put forward for clinical scrutiny while all the while observing both the safeguards of the declaration of Helsinki and ethics on animal experimentation. Our deeper understanding of the ER and its major homeostatic regulator, the UPR response, is introducing an individualized molecular approach to health management at a preventative, diagnostic and therapeutic level and, uncovering the genetic architecture underlying the ER stress response could significantly influence future therapeutic strategies in patients.

## References

[1] Porter KR , Claude A & Fullam EF (1945) A study of tissue culture cells by electron microscopy: methods and preliminary observations. J Exp Med 81, 233–246. 19871454 10.1084/jem.81.3.233 PMC2135493

[2] Palade GE & Porter KR (1954) Studies on the endoplasmic reticulum. I. Its identification in cells *in situ* . J Exp Med 100, 641–656. 13211920 10.1084/jem.100.6.641 PMC2136401

[3] Alberts B , Johnson A , Lewis J , Raff M , Roberts K & Walter P (2002) Molecular Biology of the Cell, 4th edn. Garland Science, New York, NY.

[4] Borgese N , Francolini M & Snapp E (2006) Endoplasmic reticulum architecture: structures in flux. Curr Opin Cell Biol 18, 358–364. 16806883 10.1016/j.ceb.2006.06.008 PMC4264046

[5] Shibata Y , Voeltz GK & Rapoport TA (2006) Rough sheets and smooth tubules. Cell 126, 435–439. 16901774 10.1016/j.cell.2006.07.019

[6] Hayashi T , Rizzuto R , Hajnoczky G & Su TP (2009) MAM: more than just a housekeeper. Trends Cell Biol 19, 81–88. 19144519 10.1016/j.tcb.2008.12.002 PMC2750097

[7] Toulmay A & Prinz WA (2011) Lipid transfer and signaling at organelle contact sites: the tip of the iceberg. Curr Opin Cell Biol 23, 458–463. 21555211 10.1016/j.ceb.2011.04.006 PMC3148286

[8] Daste F , Galli T & Tareste D (2015) Structure and function of longin SNAREs. J Cell Sci 128, 4263–4272. 26567219 10.1242/jcs.178574

[9] Rowland AA , Chitwood PJ , Phillips MJ & Voeltz GK (2014) ER contact sites define the position and timing of endosome fission. Cell 159, 1027–1041. 25416943 10.1016/j.cell.2014.10.023 PMC4634643

[10] Wilhelm LP , Tomasetto C & Alpy F (2016) Touché! STARD3 and STARD3NL tether the ER to endosomes. Biochem Soc Trans 44, 493–498. 27068960 10.1042/BST20150269

[11] Wilhelm LP , Wendling C , Védie B , Kobayashi T , Chenard M , Tomasetto C , Drin G & Alpy F (2017) STARD3 mediates endoplasmic reticulum‐to‐endosome cholesterol transport at membrane contact sites. EMBO J 36, 1412–1433. 28377464 10.15252/embj.201695917 PMC5430228

[12] Henne WM , Zhu L , Balogi Z , Stefan C , Pleiss JA & Emr SD (2015) Mdm1/Snx13 is a novel ER–endolysosomal interorganelle tethering protein. J Cell Biol 210, 541–551. 26283797 10.1083/jcb.201503088 PMC4539980

[13] Hayashi‐Nishino M , Fujita N , Noda T , Yamaguchi A , Yoshimori T & Yamamoto A (2010) Electron tomography reveals the endoplasmic reticulum as a membrane source for autophagosome formation. Autophagy 6, 301–303. 20104025 10.4161/auto.6.2.11134

[14] Uemura T , Yamamoto M , Kametaka A , Sou Y , Yabashi A , Yamada A , Annoh H , Kametaka S , Komatsu M & Waguri S (2014) A cluster of thin tubular structures mediates transformation of the endoplasmic reticulum to autophagic isolation membrane. Mol Cell Biol 34, 1695–1706. 24591649 10.1128/MCB.01327-13 PMC3993601

[15] Stefan CJ , Manford AG , Baird D , Yamada‐Hanff J , Mao Y & Emr SD (2011) Osh proteins regulate phosphoinositide metabolism at ER‐plasma membrane contact sites. Cell 144, 389–401. 21295699 10.1016/j.cell.2010.12.034

[16] Braakman I & Bulleid NJ (2011) Protein folding and modification in the mammalian endoplasmic reticulum. Annu Rev Biochem 80, 71–99. 21495850 10.1146/annurev-biochem-062209-093836

[17] Hebert DN & Molinari M (2007) In and out of the ER: protein folding, quality control, degradation, and related human diseases. Physiol Rev 87, 1377–1408. 17928587 10.1152/physrev.00050.2006

[18] Wallis AK & Freedman RB (2013) Assisting oxidative protein folding: how do protein disulphide‐isomerases couple conformational and chemical processes in protein folding? Top Curr Chem 328, 1–34. 21630134 10.1007/128_2011_171

[19] Aebi M , Bernasconi R , Clerc S & Molinari M (2010) N‐glycan structures: recognition and processing in the ER. Trends Biochem Sci 35, 74–82. 19853458 10.1016/j.tibs.2009.10.001

[20] Meusser B , Hirsch C , Jarosch E & Sommer T (2005) ERAD: the long road to destruction. Nat Cell Biol 7, 766–772. 16056268 10.1038/ncb0805-766

[21] Hebert DN , Bernasconi R & Molinari M (2010) ERAD substrates: which way out? Semin Cell Dev Biol 21, 526–532. 20026414 10.1016/j.semcdb.2009.12.007

[22] Kobayashi T , Tanaka K , Inoue K & Kakizuka A (2002) Functional ATPase activity of p97/valosin‐containing protein (VCP) is required for the quality control of endoplasmic reticulum in neuronally differentiated mammalian PC12 cells. J Biol Chem 277, 47358–47365. 12351637 10.1074/jbc.M207783200

[23] Enns GM , Shashi V , Bainbridge M , Gambello MJ , Zahir FR , Bast T , Crimian R , Schoch K , Platt J , Cox R *et al* (2014) Mutations in NGLY1 cause an inherited disorder of the endoplasmic reticulum‐associated degradation (ERAD) pathway. Genet Med 16, 751–758. 24651605 10.1038/gim.2014.22 PMC4243708

[24] Kamhi‐Nesher S , Shenkman M , Tolchinsky S , Fromm SV , Ehrlich R & Lederkremer GZ (2001) A novel quality control compartment derived from the endoplasmic reticulum. Mol Biol Cell 12, 1711–1723. 11408579 10.1091/mbc.12.6.1711 PMC37335

[25] Huyer G , Longsworth GL , Mason DL , Mallampalli MP , McCaffery JM , Wright RL & Michaelis S (2004) A striking quality control subcompartment in *Saccharomyces cerevisiae*: the endoplasmic reticulum‐associated compartment. Mol Biol Cell 15, 908–921. 14668485 10.1091/mbc.E03-07-0546 PMC329403

[26] Brown MS & Goldstein JL (1999) A proteolytic pathway that controls the cholesterol content of membranes, cells, and blood. Proc Natl Acad Sci USA 96, 11041–11048. 10500120 10.1073/pnas.96.20.11041 PMC34238

[27] Fagone P & Jackowski S (2009) Membrane phospholipid synthesis and endoplasmic reticulum function. J Lipid Res 50, S311–S316. 18952570 10.1194/jlr.R800049-JLR200 PMC2674712

[28] Yen C‐LE , Stone SJ , Koliwad S , Harris C & Farese RV (2008) Thematic review series: glycerolipids. DGAT enzymes and triacylglycerol biosynthesis. J Lipid Res 49, 2283–2301. 18757836 10.1194/jlr.R800018-JLR200 PMC3837458

[29] Jo Y & DeBose‐Boyd RA (2010) Control of cholesterol synthesis through regulated ER‐associated degradation of HMG CoA reductase. Crit Rev Biochem Mol Biol 45, 185–198. 20482385 10.3109/10409238.2010.485605 PMC2937355

[30] Patwardhan GA , Beverly LJ & Siskind LJ (2016) Sphingolipids and mitochondrial apoptosis. J Bioenerg Biomembr 48, 153–168. 25620271 10.1007/s10863-015-9602-3 PMC5434644

[31] Barlowe C , Orci L , Yeung T , Hosobuchi M , Hamamoto S , Salama N , Rexach MF , Ravazzola M , Amherdt M & Schekman R (1994) COPII: a membrane coat formed by Sec proteins that drive vesicle budding from the endoplasmic reticulum. Cell 77, 895–907. 8004676 10.1016/0092-8674(94)90138-4

[32] Siddiqi S , Saleem U , Abumrad NA , Davidson NO , Storch J , Siddiqi SA & Mansbach CM (2010) A novel multiprotein complex is required to generate the prechylomicron transport vesicle from intestinal ER. J Lipid Res 51, 1918–1928. 20237389 10.1194/jlr.M005611 PMC2882727

[33] Clapham DE (2007) Calcium signaling. Cell 131, 1047–1058. 18083096 10.1016/j.cell.2007.11.028

[34] Meldolesi J & Pozzan T (1998) The endoplasmic reticulum Ca2 + store: a view from the lumen. Trends Biochem Sci 23, 10–14. 9478128 10.1016/s0968-0004(97)01143-2

[35] Hetz C (2012) The unfolded protein response: controlling cell fate decisions under ER stress and beyond. Nat Rev Mol Cell Biol 13, 89–102. 22251901 10.1038/nrm3270

[36] Hanahan D & Weinberg RA (2011) Hallmarks of cancer: the next generation. Cell 144, 646–674. 21376230 10.1016/j.cell.2011.02.013

[37] Holderfield M , Deuker MM , McCormick F & McMahon M (2014) Targeting RAF kinases for cancer therapy: BRAF mutated melanoma and beyond. Nat Rev Cancer 14, 455–467. 24957944 10.1038/nrc3760 PMC4250230

[38] Piwocka K , Vejda S , Cotter TG , O'Sullivan GC & McKenna SL (2006) Bcr‐Abl reduces endoplasmic reticulum releasable calcium levels by a Bcl‐2‐independent mechanism and inhibits calcium‐dependent apoptotic signaling. Blood 107, 4003–4010. 16469868 10.1182/blood-2005-04-1523

[39] Pluquet O , Pourtier A & Abbadie C (2015) The unfolded protein response and cellular senescence. A review in the theme: cellular mechanisms of endoplasmic reticulum stress signaling in health and disease. Am J Physiol Cell Physiol 308, C415–C425. 25540175 10.1152/ajpcell.00334.2014

[40] Chen H‐J , Anagnostou G , Chai A , Withers J , Morris A , Adhikaree J , Pennetta G & de Belleroche JS (2010) Characterization of the properties of a novel mutation in VAPB in familial amyotrophic lateral sclerosis. J Biol Chem 285, 40266–40281. 20940299 10.1074/jbc.M110.161398 PMC3001007

[41] Nishimura AL , Mitne‐Neto M , Silva HCA , Richieri‐Costa A , Middleton S , Cascio D , Kok F , Oliveira JRM , Gillingwater T , Webb J *et al* (2004) A Mutation in the vesicle‐trafficking protein VAPB causes late‐onset spinal muscular atrophy and amyotrophic lateral sclerosis. Am J Hum Genet 75, 822–831. 15372378 10.1086/425287 PMC1182111

[42] Harding HP & Ron D (2002) Endoplasmic reticulum stress and the development of diabetes: a review. Diabetes 51, S455–S461. 12475790 10.2337/diabetes.51.2007.s455

[43] Oyadomari S , Koizumi A , Takeda K , Gotoh T , Akira S , Araki E & Mori M (2002) Targeted disruption of the Chop gene delays endoplasmic reticulum stress‐mediated diabetes. J Clin Invest 109, 525–532. 11854325 10.1172/JCI14550 PMC150879

[44] Colombo C , Porzio O , Liu M , Massa O , Vasta M , Salardi S , Beccaria L , Monciotti C , Toni S , Pedersen O *et al* (2008) Seven mutations in the human insulin gene linked to permanent neonatal/infancy‐onset diabetes mellitus. J Clin Invest 118, 2148–2156. 18451997 10.1172/JCI33777 PMC2350430

[45] Støy J , Edghill EL , Flanagan SE , Ye H , Paz VP , Pluzhnikov A , Below JE , Hayes MG , Cox NJ , Lipkind GM *et al* (2007) Insulin gene mutations as a cause of permanent neonatal diabetes. Proc Natl Acad Sci USA 104, 15040–15044. 17855560 10.1073/pnas.0707291104 PMC1986609

[46] Giampietri C , Petrungaro S , Conti S , Facchiano A , Filippini A & Ziparo E (2015) Cancer microenvironment and endoplasmic reticulum stress response. Mediators Inflamm 2015, 417281. 26491226 10.1155/2015/417281 PMC4600498

[47] Jin ML , Park SY , Kim YH , Oh J‐I , Lee SJ & Park G (2014) The neuroprotective effects of cordycepin inhibit glutamate‐induced oxidative and ER stress‐associated apoptosis in hippocampal HT22 cells. Neurotoxicology 41, 102–111. 24486958 10.1016/j.neuro.2014.01.005

[48] Stechmann B , Bai S‐K , Gobbo E , Lopez R , Merer G , Pinchard S , Panigai L , Tenza D , Raposo G , Beaumelle B *et al* (2010) Inhibition of retrograde transport protects mice from lethal ricin challenge. Cell 141, 231–242. 20403321 10.1016/j.cell.2010.01.043

[49] Olden K , Pratt RM , Jaworski C & Yamada KM (1979) Evidence for role of glycoprotein carbohydrates in membrane transport: specific inhibition by tunicamycin. Proc Natl Acad Sci USA 76, 791–795. 218220 10.1073/pnas.76.2.791 PMC383052

[50] Schultz AM & Oroszlan S (1979) Tunicamycin inhibits glycosylation of precursor polyprotein encoded by env gene of Rauscher murine leukemia virus. Biochem Biophys Res Commun 86, 1206–1213. 435320 10.1016/0006-291x(79)90245-6

[51] Datema R & Schwarz RT (1979) Interference with glycosylation of glycoproteins. Inhibition of formation of lipid‐linked oligosaccharides *in vivo* . Biochem J 184, 113–123. 534512 10.1042/bj1840113 PMC1161681

[52] Cleland WW (1964) Dithiothreitol, a new protective reagent for SH groups. Biochemistry 3, 480–482. 14192894 10.1021/bi00892a002

[53] Liu ES , Ou JH & Lee AS (1992) Brefeldin A as a regulator of grp78 gene expression in mammalian cells. J Biol Chem 267, 7128–7133. 1551919

[54] Thastrup O , Cullen PJ , Drøbak BK , Hanley MR & Dawson AP (1990) Thapsigargin, a tumor promoter, discharges intracellular Ca2 + stores by specific inhibition of the endoplasmic reticulum Ca2(+)‐ATPase. Proc Natl Acad Sci USA 87, 2466–2470. 2138778 10.1073/pnas.87.7.2466 PMC53710

[55] Pirot P , Naamane N , Libert F , Magnusson NE , Ørntoft TF , Cardozo AK & Eizirik DL (2007) Global profiling of genes modified by endoplasmic reticulum stress in pancreatic beta cells reveals the early degradation of insulin mRNAs. Diabetologia 50, 1006–1014. 17333111 10.1007/s00125-007-0609-0

[56] Malo A , Krüger B , Göke B & Kubisch CH (2013) 4‐phenylbutyric acid reduces endoplasmic reticulum stress, trypsin activation, and acinar cell apoptosis while increasing secretion in rat pancreatic acini. Pancreas 42, 92–101. 22889983 10.1097/MPA.0b013e318259f6ca

[57] Lee YY , Hong SH , Lee YJ , Chung SS , Jung HS , Park SG & Park KS (2010) Tauroursodeoxycholate (TUDCA), chemical chaperone, enhances function of islets by reducing ER stress. Biochem Biophys Res Commun 397, 735–739. 20541525 10.1016/j.bbrc.2010.06.022

[58] Ozcan U , Yilmaz E , Ozcan L , Furuhashi M , Vaillancourt E , Smith RO , Görgün CZ & Hotamisligil GS (2006) Chemical chaperones reduce ER stress and restore glucose homeostasis in a mouse model of type 2 diabetes. Science 313, 1137–1140. 16931765 10.1126/science.1128294 PMC4741373

[59] Lepock JR (2005) How do cells respond to their thermal environment? Int J Hyperthermia 21, 681–687. 16338849 10.1080/02656730500307298

[60] Bettaieb A & Averill‐Bates DA (2005) Thermotolerance induced at a mild temperature of 40°C protects cells against heat shock‐induced apoptosis. J Cell Physiol 205, 47–57. 15887240 10.1002/jcp.20386

[61] Liu Y , Sakamoto H , Adachi M , Zhao S , Ukai W , Hashimoto E , Hareyama M , Ishida T , Imai K & Shinomura Y (2012) Heat stress activates ER stress signals which suppress the heat shock response, an effect occurring preferentially in the cortex in rats. Mol Biol Rep 39, 3987–3993. 21779805 10.1007/s11033-011-1179-2

[62] Liu X , Wang M , Chen H , Guo Y , Ma F , Shi F , Bi Y & Li Y (2013) Hypothermia protects the brain from transient global ischemia/reperfusion by attenuating endoplasmic reticulum response‐induced apoptosis through CHOP. PLoS ONE 8, e53431. 23301071 10.1371/journal.pone.0053431 PMC3536702

[63] Mollereau B (2015) Cooling‐induced ER stress is good for your brain. EBioMedicine 2, 482–483. 26288807 10.1016/j.ebiom.2015.05.008 PMC4535306

[64] Zeeshan H , Lee G , Kim H‐R & Chae H‐J (2016) Endoplasmic reticulum stress and associated ROS. Int J Mol Sci 17, 327. 26950115 10.3390/ijms17030327 PMC4813189

[65] Bhandary B , Marahatta A , Kim H‐R & Chae H‐J (2012) An involvement of oxidative stress in endoplasmic reticulum stress and its associated diseases. Int J Mol Sci 14, 434–456. 23263672 10.3390/ijms14010434 PMC3565273

[66] Cadenas E & Davies KJ (2000) Mitochondrial free radical generation, oxidative stress, and aging. Free Radic Biol Med 29, 222–230. 11035250 10.1016/s0891-5849(00)00317-8

[67] Knupp J , Arvan P & Chang A (2018) Increased mitochondrial respiration promotes survival from endoplasmic reticulum stress. Cell Death Differ. 10.1038/s41418-018-0133-4 PMC6370866 29795335

[68] Tu BP & Weissman JS (2004) Oxidative protein folding in eukaryotes: mechanisms and consequences. J Cell Biol 164, 341–346. 14757749 10.1083/jcb.200311055 PMC2172237

[69] Malhotra JD & Kaufman RJ (2007) Endoplasmic reticulum stress and oxidative stress: a vicious cycle or a double‐edged sword? Antioxid Redox Signal 9, 2277–2293. 17979528 10.1089/ars.2007.1782

[70] Cao SS & Kaufman RJ (2014) Endoplasmic reticulum stress and oxidative stress in cell fate decision and human disease. Antioxid Redox Signal 21, 396–413. 24702237 10.1089/ars.2014.5851 PMC4076992

[71] Montibeller L & de Belleroche J (2018) Amyotrophic lateral sclerosis (ALS) and Alzheimer's disease (AD) are characterised by differential activation of ER stress pathways: focus on UPR target genes. Cell Stress Chaperones. 10.1007/s12192-018-0897-y PMC6111088 29725981

[72] Bertolotti A , Zhang Y , Hendershot LM , Harding HP & Ron D (2000) Dynamic interaction of BiP and ER stress transducers in the unfolded‐protein response. Nat Cell Biol 2, 326–332. 10854322 10.1038/35014014

[73] Shen J , Chen X , Hendershot L & Prywes R (2002) ER stress regulation of ATF6 localization by dissociation of BiP/GRP78 binding and unmasking of Golgi localization signals. Dev Cell 3, 99–111. 12110171 10.1016/s1534-5807(02)00203-4

[74] Kopp MC , Nowak PR , Larburu N , Adams CJ & Ali MMU (2018) *In vitro* FRET analysis of IRE1 and BiP association and dissociation upon endoplasmic reticulum stress. Elife 7, e30257. 29303481 10.7554/eLife.30257 PMC5756023

[75] Amin‐Wetzel N , Saunders RA , Kamphuis MJ , Rato C , Preissler S , Harding HP & Ron D (2017) A J‐Protein co‐chaperone recruits BiP to monomerize IRE1 and repress the unfolded protein response. Cell 171, 1625–1637.e13. 29198525 10.1016/j.cell.2017.10.040 PMC5733394

[76] Sepulveda D , Rojas‐Rivera D , Rodríguez DA , Groenendyk J , Köhler A , Lebeaupin C , Ito S , Urra H , Carreras‐Sureda A , Hazari Y *et al* (2018) Interactome screening identifies the ER luminal chaperone Hsp47 as a regulator of the unfolded protein response transducer IRE1α. Mol Cell 69, 238–252.e7. 29351844 10.1016/j.molcel.2017.12.028

[77] Ye J , Rawson RB , Komuro R , Chen X , Davé UP , Prywes R , Brown MS & Goldstein JL (2000) ER stress induces cleavage of membrane‐bound ATF6 by the same proteases that process SREBPs. Mol Cell 6, 1355–1364. 11163209 10.1016/s1097-2765(00)00133-7

[78] Carrara M , Prischi F & Ali MMU (2013) UPR signal activation by luminal sensor domains. Int J Mol Sci 14, 6454–6466. 23519110 10.3390/ijms14036454 PMC3634511

[79] Tirasophon W , Welihinda AA & Kaufman RJ (1998) A stress response pathway from the endoplasmic reticulum to the nucleus requires a novel bifunctional protein kinase/endoribonuclease (Ire1p) in mammalian cells. Genes Dev 12, 1812–1824. 9637683 10.1101/gad.12.12.1812 PMC316900

[80] Wang XZ , Harding HP , Zhang Y , Jolicoeur EM , Kuroda M & Ron D (1998) Cloning of mammalian Ire1 reveals diversity in the ER stress responses. EMBO J 17, 5708–5717. 9755171 10.1093/emboj/17.19.5708 PMC1170899

[81] Iwawaki T , Hosoda A , Okuda T , Kamigori Y , Nomura‐Furuwatari C , Kimata Y , Tsuru A & Kohno K (2001) Translational control by the ER transmembrane kinase/ribonuclease IRE1 under ER stress. Nat Cell Biol 3, 158–165. 11175748 10.1038/35055065

[82] Bertolotti A , Wang X , Novoa I , Jungreis R , Schlessinger K , Cho JH , West AB & Ron D (2001) Increased sensitivity to dextran sodium sulfate colitis in IRE1β‐deficient mice. J Clin Invest 107, 585–593. 11238559 10.1172/JCI11476 PMC199427

[83] Martino MB , Jones L , Brighton B , Ehre C , Abdulah L , Davis CW , Ron D , O'Neal WK & Ribeiro CMP (2013) The ER stress transducer IRE1β is required for airway epithelial mucin production. Mucosal Immunol 6, 639–654. 23168839 10.1038/mi.2012.105 PMC4031691

[84] Iwawaki T , Akai R , Yamanaka S & Kohno K (2009) Function of IRE1 alpha in the placenta is essential for placental development and embryonic viability. Proc Natl Acad Sci USA 106, 16657–16662. 19805353 10.1073/pnas.0903775106 PMC2757843

[85] Bouchecareilh M , Higa A , Fribourg S , Moenner M & Chevet E (2011) Structure of the Ire1 autophosphorylation complex and implications for the unfolded protein response. FASEB J 25, 3115–3129. 21680894 10.1096/fj.11-182931

[86] Sanches M , Duffy NM , Talukdar M , Thevakumaran N , Chiovitti D , Canny MD , Lee K , Kurinov I , Uehling D , Al‐awar R *et al* (2014) Structure and mechanism of action of the hydroxy‐aryl‐aldehyde class of IRE1 endoribonuclease inhibitors. Nat Commun 5, 4202. 25164867 10.1038/ncomms5202 PMC4486471

[87] Prischi F , Nowak PR , Carrara M & Ali MMU (2014) Phosphoregulation of Ire1 RNase splicing activity. Nat Commun 5, 3554. 24704861 10.1038/ncomms4554 PMC3988810

[88] Urano F , Wang X‐Z , Bertolotti A , Zhang Y , Chung P , Harding HP & Ron D (2000) Coupling of stress in the endoplasmic reticulum to activation of JNK protein kinases by transmembrane protein kinase IRE1. Science 287, 664–666. 10650002 10.1126/science.287.5453.664

[89] Bork P & Sander C (1993) A hybrid protein kinase‐RNase in an interferon‐induced pathway? FEBS Lett 334, 149–152. 7693513 10.1016/0014-5793(93)81701-z

[90] Sidrauski C & Walter P (1997) The transmembrane kinase Ire1p is a site‐specific endonuclease that initiates mRNA splicing in the unfolded protein response. Cell 90, 1031–1039. 9323131 10.1016/s0092-8674(00)80369-4

[91] Yoshida H , Matsui T , Yamamoto A , Okada T & Mori K (2001) XBP1 mRNA is induced by ATF6 and spliced by IRE1 in response to ER stress to produce a highly active transcription factor. Cell 107, 881–891. 11779464 10.1016/s0092-8674(01)00611-0

[92] Greer CL , Peebles CL , Gegenheimer P & Abelson J (1983) Mechanism of action of a yeast RNA ligase in tRNA splicing. Cell 32, 537–546. 6297798 10.1016/0092-8674(83)90473-7

[93] Sidrauski C , Cox JS & Walter P (1996) tRNA ligase is required for regulated mRNA splicing in the unfolded protein response. Cell 87, 405–413. 8898194 10.1016/s0092-8674(00)81361-6

[94] Schwer B , Sawaya R , Ho CK & Shuman S (2004) Portability and fidelity of RNA‐repair systems. Proc Natl Acad Sci USA 101, 2788–2793. 14973195 10.1073/pnas.0305859101 PMC365698

[95] Tanaka N , Meineke B & Shuman S (2011) RtcB, a novel RNA ligase, can catalyze tRNA splicing and HAC1 mRNA splicing *in vivo* . J Biol Chem 286, 30253–30257. 21757685 10.1074/jbc.C111.274597 PMC3162383

[96] Baltz AG , Munschauer M , Schwanhäusser B , Vasile A , Murakawa Y , Schueler M , Youngs N , Penfold‐Brown D , Drew K , Milek M *et al* (2012) The mRNA‐bound proteome and its global occupancy profile on protein‐coding transcripts. Mol Cell 46, 674–690. 22681889 10.1016/j.molcel.2012.05.021

[97] Lu Y , Liang FX & Wang X (2014) A synthetic biology approach identifies the mammalian UPR RNA ligase RtcB. Mol Cell 55, 758–770. 25087875 10.1016/j.molcel.2014.06.032 PMC4156904

[98] Liou HC , Boothby MR , Finn PW , Davidon R , Nabavi N , Zeleznik‐Le NJ , Ting JP & Glimcher LH (1990) A new member of the leucine zipper class of proteins that binds to the HLA DR alpha promoter. Science 247, 1581–1584. 2321018 10.1126/science.2321018

[99] Yoshimura T , Fujisawa J & Yoshida M (1990) Multiple cDNA clones encoding nuclear proteins that bind to the tax‐dependent enhancer of HTLV‐1: all contain a leucine zipper structure and basic amino acid domain. EMBO J 9, 2537–2542. 2196176 10.1002/j.1460-2075.1990.tb07434.x PMC552284

[100] Nojima H , Leem Sh , Araki H , Sakai A , Nakashima N , Kanaoka Y & Ono Y (1994) Hac1: a novel yeast bZIP protein binding to the CRE motif is a multicopy suppressor for cdcW mutant of *Schizosaccharomyces pombe* . Nucleic Acids Res 22, 5279–5288. 7816617 10.1093/nar/22.24.5279 PMC332072

[101] Cox JS & Walter P (1996) A novel mechanism for regulating activity of a transcription factor that controls the unfolded protein response. Cell 87, 391–404. 8898193 10.1016/s0092-8674(00)81360-4

[102] Calfon M , Zeng H , Urano F , Till JH , Hubbard SR , Harding HP , Clark SG & Ron D (2002) IRE1 couples endoplasmic reticulum load to secretory capacity by processing the XBP‐1 mRNA. Nature 415, 92–96. 11780124 10.1038/415092a

[103] Chen CY , Malchus NS , Hehn B , Stelzer W , Avci D , Langosch D & Lemberg MK (2014) Signal peptide peptidase functions in ERAD to cleave the unfolded protein response regulator XBP1u. EMBO J 33, 2492–2506. 25239945 10.15252/embj.201488208 PMC4283407

[104] Yanagitani K , Imagawa Y , Iwawaki T , Hosoda A , Saito M , Kimata Y & Kohno K (2009) Cotranslational Targeting of XBP1 protein to the membrane promotes cytoplasmic splicing of its own mRNA. Mol Cell 34, 191–200. 19394296 10.1016/j.molcel.2009.02.033

[105] Yanagitani K , Kimata Y , Kadokura H & Kohno K (2011) Translational pausing ensures membrane targeting and cytoplasmic splicing of XBP1u mRNA. Science 331, 586–589. 21233347 10.1126/science.1197142

[106] Kanda S , Yanagitani K , Yokota Y , Esaki Y & Kohno K (2016) Autonomous translational pausing is required for XBP1u mRNA recruitment to the ER via the SRP pathway. Proc Natl Acad Sci 113, E5886–E5895. 27651490 10.1073/pnas.1604435113 PMC5056097

[107] Chalmers F , Sweeney B , Cain K & Bulleid NJ (2017) Inhibition of IRE1α‐mediated XBP1 mRNA cleavage by XBP1 reveals a novel regulatory process during the unfolded protein response. Wellcome Open Res 2, 36. 29062910 10.12688/wellcomeopenres.11764.2 PMC5645705

[108] He Y , Sun S , Sha H , Liu Z , Yang L , Xue Z , Chen H & Qi L (2010) Emerging roles for XBP1, a sUPeR transcription factor. Gene Expr 15, 13–25. 21061914 10.3727/105221610x12819686555051 PMC3374844

[109] Travers KJ , Patil CK , Wodicka L , Lockhart DJ , Weissman JS & Walter P (2000) Functional and genomic analyses reveal an essential coordination between the unfolded protein response and ER‐associated degradation. Cell 101, 249–258. 10847680 10.1016/s0092-8674(00)80835-1

[110] Iwakoshi NN , Lee A‐H & Glimcher LH (2003) The X‐box binding protein‐1 transcription factor is required for plasma cell differentiation and the unfolded protein response. Immunol Rev 194, 29–38. 12846805 10.1034/j.1600-065x.2003.00057.x

[111] Sriburi R , Jackowski S , Mori K & Brewer JW (2004) XBP1: a link between the unfolded protein response, lipid biosynthesis, and biogenesis of the endoplasmic reticulum. J Cell Biol 167, 35–41. 15466483 10.1083/jcb.200406136 PMC2172532

[112] Korchak HM (2008) Regulation of hepatic lipogenesis. Tufts Folia Med 8, 134–143. 14034742

[113] So JS , Hur KY , Tarrio M , Ruda V , Frank‐Kamenetsky M , Fitzgerald K , Koteliansky V , Lichtman AH , Iwawaki T , Glimcher LH *et al* (2012) Silencing of lipid metabolism genes through IRE1α‐mediated mRNA decay lowers plasma lipids in mice. Cell Metab 16, 487–499. 23040070 10.1016/j.cmet.2012.09.004 PMC3475419

[114] Zhou Y , Lee J , Reno CM , Sun C , Park SW , Chung J , Lee J , Fisher SJ , White MF , Biddinger SB *et al* (2011) Regulation of glucose homeostasis through a XBP‐1–FoxO1 interaction. Nat Med 17, 356–365. 21317886 10.1038/nm.2293 PMC3897616

[115] Park SW , Herrema H , Salazar M , Cakir I , Cabi S , Basibuyuk Sahin F , Chiu YH , Cantley LC & Ozcan U (2014) BRD7 regulates XBP1s’ activity and glucose homeostasis through its interaction with the regulatory subunits of PI3K. Cell Metab 20, 73–84. 24836559 10.1016/j.cmet.2014.04.006 PMC4079724

[116] Liu J , Ibi D , Taniguchi K , Lee J , Herrema H , Akosman B , Mucka P , Salazar Hernandez MA , Uyar MF , Park SW *et al* (2016) Inflammation improves glucose homeostasis through IKKβ‐XBP1s interaction. Cell 167, 1052–1066.e18. 27814504 10.1016/j.cell.2016.10.015 PMC5908236

[117] Lee J , Sun C , Zhou Y , Lee J , Gokalp D , Herrema H , Park SW , Davis RJ & Ozcan U (2011) p38 MAPK–mediated regulation of Xbp1s is crucial for glucose homeostasis. Nat Med 17, 1251–1260. 21892182 10.1038/nm.2449 PMC4397266

[118] Yingfeng D , Zhao WV , Caroline T , Ningguo G , William HL , Anwarul F , Joyce RJ , Guosheng L , Jin Y , Mark LA *et al* (2013) The Xbp1s/GalE axis links ER stress to postprandial hepatic metabolism. J Clin Invest 123, 455–468. 23257357 10.1172/JCI62819 PMC3533268

[119] Özcan U , Cao Q , Yilmaz E , Lee A‐H , Iwakoshi NN , Özdelen E , Tuncman G , Görgün C , Glimcher Laurie H & Hotamisligil GS (2004) Endoplasmic reticulum stress links obesity, insulin action, and type 2 diabetes. Science 306, 457–461. 15486293 10.1126/science.1103160

[120] Akiyama M , Liew CW , Lu S , Hu J , Martinez R , Hambro B , Kennedy RT & Kulkarni RN (2013) X‐box binding protein 1 is essential for insulin regulation of pancreatic a‐cell function. Diabetes 62, 2439–2449. 23493568 10.2337/db12-1747 PMC3712068

[121] Liu Y , Adachi M , Zhao S , Hareyama M , Koong AC , Luo D & Rando TA (2010) Preventing oxidative stress a new role for XBP1. Cell 16, 847–857. 10.1038/cdd.2009.14 PMC2826168 19247368

[122] Tao R , Chen H , Gao C , Xue P , Yang F , Han J‐DJ , Zhou B & Chen Y‐G (2011) Xbp1‐mediated histone H4 deacetylation contributes to DNA double‐strand break repair in yeast. Cell Res 21, 1619–1633. 21467995 10.1038/cr.2011.58 PMC3364731

[123] Wu J & Kaufman RJ (2006) From acute ER stress to physiological roles of the Unfolded Protein Response. Cell Death Differ 13, 374–384. 16397578 10.1038/sj.cdd.4401840

[124] Blais A , Tsikitis M , Acosta‐Alvear D , Sharan R , Kluger Y & Dynlacht BD (2005) An initial blueprint for myogenic differentiation. Genes Dev 19, 553–569. 15706034 10.1101/gad.1281105 PMC551576

[125] Reimold AM , Iwakoshi NN , Manis J , Vallabhajosyula P , Szomolanyi‐Tsuda E , Gravallese EM , Friend D , Grusby MJ , Alt F & Glimcher LH (2001) Plasma cell differentiation requires the transcription factor XBP‐1. Nature 412, 300–307. 11460154 10.1038/35085509

[126] Lee A‐H , Chu GC , Iwakoshi NN & Glimcher LH (2005) XBP‐1 is required for biogenesis of cellular secretory machinery of exocrine glands. EMBO J 24, 4368–4380. 16362047 10.1038/sj.emboj.7600903 PMC1356340

[127] Huh WJ , Esen E , Geahlen JH , Bredemeyer AJ , Lee A , Shi G , Konieczny SF , Glimcher LH & Mills JC (2010) XBP1 controls maturation of gastric zymogenic cells by induction of MIST1 and expansion of the rough endoplasmic reticulum. Gastroenterology 139, 2038–2049. 20816838 10.1053/j.gastro.2010.08.050 PMC2997137

[128] Sha H , He Y , Chen H , Wang C , Zenno A , Shi H , Yang X & Zhang X (2009) The IRE1α‐XBP1 pathway of the unfolded protein response is required for adipogenesis. Cell Metab 9, 556–564. 19490910 10.1016/j.cmet.2009.04.009 PMC2963107

[129] Masaki T , Yoshida M & Noguchi S (1999) Targeted disruption of CRE‐Binding factor TREB5 gene leads to cellular necrosis in cardiac myocytes at the embryonic stage. Biochem Biophys Res Commun 261, 350–356. 10425189 10.1006/bbrc.1999.0972

[130] Reimold AM , Etkin A , Clauss I , Perkins A , Friend DS , Zhang J , Horton HF , Scott A , Orkin SH , Byrne MC *et al* (2000) An essential role in liver development for transcription factor XBP‐1. Genes Dev 14, 152–157. 10652269 PMC316338

[131] Sone M , Zeng X , Larese J & Ryoo HD (2013) A modified UPR stress sensing system reveals a novel tissue distribution of IRE1/XBP1 activity during normal Drosophila development. Cell Stress Chaperones 18, 307–319. 23160805 10.1007/s12192-012-0383-x PMC3631089

[132] Ono SJ , Liout H , Davidont R , Strominger JL & Glimchert LH (1991) Human X‐box‐binding protein 1 is required for the transcription of a subset of human class II major histocompatibility genes and forms a heterodimer with c‐fos. Proc Natl Acad Sci USA 88, 4309–4312. 1903538 10.1073/pnas.88.10.4309 PMC51648

[133] Ding L , Yan J , Zhu J , Zhong H , Lu Q , Wang Z , Huang C & Ye Q (2003) Ligand‐independent activation of estrogen receptor α by XBP‐1. Nucleic Acids Res 31, 5266–5274. 12954762 10.1093/nar/gkg731 PMC203316

[134] Ravasi T , Suzuki H , Cannistraci CV , Katayama S , Bajic VB , Tan K , Akalin A , Schmeier S , Kanamori‐Katayama M , Bertin N *et al* (2010) An atlas of combinatorial transcriptional regulation in mouse and man. Cell 140, 744–752. 20211142 10.1016/j.cell.2010.01.044 PMC2836267

[135] Reinke AW , Baek J , Ashenberg O & Keating AE (2013) Networks of bZIP protein‐protein interactions diversified over a billion years of evolution. Science 340, 730–734. 23661758 10.1126/science.1233465 PMC4115154

[136] Chen X , Iliopoulos D , Zhang Q , Tang Q , Greenblatt MB , Hatziapostolou M , Lim E , Tam WL , Ni M , Chen Y *et al* (2014) XBP1 promotes triple‐negative breast cancer by controlling the HIF1α pathway. Nature 508, 103–107. 24670641 10.1038/nature13119 PMC4105133

[137] Hollien J , Lin JH , Li H , Stevens N , Walter P & Weissman JS (2009) Regulated Ire1‐dependent decay of messenger RNAs in mammalian cells. J Cell Biol 186, 323–331. 19651891 10.1083/jcb.200903014 PMC2728407

[138] Maurel M , Chevet E , Tavernier J & Gerlo S (2014) Getting RIDD of RNA: IRE1 in cell fate regulation. Trends Biochem Sci 39, 245–254. 24657016 10.1016/j.tibs.2014.02.008

[139] Lhomond S , Avril T , Dejeans N , Voutetakis K , Doultsinos D , McMahon M , Pineau R , Obacz J , Papadodima O , Jouan F *et al* (2018) Dual IRE1 RNase functions dictate glioblastoma development. EMBO Mol Med 10, e7929 10.15252/emmm.201707929 29311133 PMC5840541

[140] Tirasophon W , Lee K , Callaghan B , Welihinda A & Kaufman RJ (2000) The endoribonuclease activity of mammalian IRE1 autoregulates its mRNA and is required for the unfolded protein response. Genes Dev 14, 2725–2736. 11069889 10.1101/gad.839400 PMC317029

[141] Hollien J & Weissman JS (2006) Decay of endoplasmic reticulum‐localized mRNAs during the unfolded protein response. Science 313, 104–107. 16825573 10.1126/science.1129631

[142] Han D , Lerner AG , Vande WL , Upton J‐P , Xu W , Hagen A , Backes BJ , Oakes SA & Papa FR (2009) IRE1α kinase activation modes control alternate endoribonuclease outputs to determine divergent cell fates. Cell 138, 562–575. 19665977 10.1016/j.cell.2009.07.017 PMC2762408

[143] Kimmig P , Diaz M , Zheng J , Williams CC , Lang A , Aragón T , Li H & Walter P (2012) The unfolded protein response in fission yeast modulates stability of select mRNAs to maintain protein homeostasis. Elife 2012, 1–20. 10.7554/eLife.00048 PMC3470409 23066505

[144] Mishiba K‐0049 , Nagashima Y , Suzuki E , Hayashi N , Ogata Y , Shimada Y & Koizumi N (2013) Defects in IRE1 enhance cell death and fail to degrade mRNAs encoding secretory pathway proteins in the Arabidopsis unfolded protein response. Proc Natl Acad Sci 110, 5713–5718. 23509268 10.1073/pnas.1219047110 PMC3619347

[145] Hayashi S , Wakasa Y , Ozawa K & Takaiwa F (2016) Characterization of IRE1 ribonuclease‐mediated mRNA decay in plants using transient expression analyses in rice protoplasts. New Phytol 210, 1259–1268. 26831622 10.1111/nph.13845

[146] Oikawa D , Tokuda M , Hosoda A & Iwawaki T (2010) Identification of a consensus element recognized and cleaved by IRE1α. Nucleic Acids Res 38, 6265–6273. 20507909 10.1093/nar/gkq452 PMC2952871

[147] Iqbal J , Dai K , Seimon T , Jungreis R , Oyadomari M , Kuriakose G , Ron D , Tabas I & Hussain MM (2008) IRE1β Inhibits chylomicron production by selectively degrading MTP mRNA. Cell Metab 7, 445–455. 18460335 10.1016/j.cmet.2008.03.005 PMC2435513

[148] Imagawa Y , Hosoda A , Sasaka S , Tsuru A & Kohno K (2008) RNase domains determine the functional difference between IRE1α and IRE1β. FEBS Lett 582, 656–660. 18242182 10.1016/j.febslet.2008.01.038

[149] Shi Y , Vattem KM , Sood R , An J , Liang J , Stramm L & Wek RC (1998) Identification and characterization of pancreatic eukaryotic initiation factor 2 alpha‐subunit kinase, PEK, involved in translational control. Mol Cell Biol 18, 7499–7509. 9819435 10.1128/mcb.18.12.7499 PMC109330

[150] Harding HP , Zhang Y & Ron D (1999) Protein translation and folding are coupled by an endoplasmic‐reticulum‐resident kinase. Nature 397, 271–274. 9930704 10.1038/16729

[151] McQuiston A & Diehl JA (2017) Recent insights into PERK‐dependent signaling from the stressed endoplasmic reticulum. F1000Research 6, 1897. 29152224 10.12688/f1000research.12138.1 PMC5664976

[152] Lloyd MA , Osborne JC , Safer B , Powell GM & Merrick WC (1980) Characteristics of eukaryotic initiation factor 2 and its subunits. J Biol Chem 255, 1189–1193. 6153180

[153] Ernst H , Duncan RF & Hershey JWB (1987) Cloning and sequencing of complementary DNAs encoding the alpha‐subunit of translational initiation factor‐Eif‐2 ‐ characterization of the protein and its messenger RNA. J Biol Chem 262, 1206–1212. 2948954

[154] Adams SL , Safer B & Anderson WFMW (1975) Eukaryotic initiation complex formation. Evidence for two distinct pathways. J Biol Chem 250, 9083–9089. 1194278

[155] Rowlands AG , Panniers R & Henshaw EC (1988) The catalytic mechanism of guanine nucleotide exchange factor action and competitive inhibition by phosphorylated eukaryotic initiation factor 2. J Biol Chem 263, 5526–5533. 3356695

[156] Harding HP , Zhang Y , Bertolotti A , Zeng H & Ron D (2000) Perk is essential for translational regulation and cell survival during the unfolded protein response. Mol Cell 5, 897–904. 10882126 10.1016/s1097-2765(00)80330-5

[157] Hai T , Liu F , Coukos WJ & Green MR (1989) Transcription factor ATF cDNA clones: an extensive family of leucine zipper proteins able to selectively form DNA‐binding heterodimers. Genes Dev 3, 2083–2090. 2516827 10.1101/gad.3.12b.2083

[158] Harding HP , Novoa I , Zhang Y , Zeng H , Wek R , Schapira M & Ron D (2000) Regulated translation initiation controls stress‐induced gene expression in mammalian cells. Mol Cell 6, 1099–1108. 11106749 10.1016/s1097-2765(00)00108-8

[159] Vallejo M , Ron D , Miller CP & Habener JF (1993) C/ATF, a member of the activating transcription factor family of DNA‐binding proteins, dimerizes with CAAT/enhancer‐binding proteins and directs their binding to cAMP response elements. Proc Natl Acad Sci USA 90, 4679–4683. 8506317 10.1073/pnas.90.10.4679 PMC46576

[160] Fawcett TW , Martindale JL , Guyton KZ , Hai T & Holbrook NJ (1999) Complexes containing activating transcription factor (ATF)/cAMP‐responsive‐element‐binding protein (CREB) interact with the CCAAT/enhancer‐binding protein (C/EBP)–ATF composite site to regulate Gadd153 expression during the stress response. Biochem J 339, 135–141. 10085237 PMC1220137

[161] Han J , Back SH , Hur J , Lin Y , Gildersleeve R , Shan J , Yuan CL , Krokowski D , Wang S , Hatzoglou M *et al* (2013) ER‐stress‐induced transcriptional regulation increases protein synthesis leading to cell death. Nat Cell Biol 15, 481–490. 23624402 10.1038/ncb2738 PMC3692270

[162] Hiramatsu N , Messah C , Han J , LaVail MM , Kaufman RJ & Lin JH (2014) Translational and posttranslational regulation of XIAP by eIF2α and ATF4 promotes ER stress‐induced cell death during the unfolded protein response. Mol Biol Cell 25, 1411–1420. 24623724 10.1091/mbc.E13-11-0664 PMC4004591

[163] Chen YJ , Tan BCM , Cheng YY , Chen JS & Lee SC (2009) Differential regulation of CHOP translation by phosphorylated eIF4E under stress conditions. Nucleic Acids Res 38, 764–777. 19934253 10.1093/nar/gkp1034 PMC2817461

[164] Palam LR , Baird TD & Wek RC (2011) Phosphorylation of eIF2 facilitates ribosomal bypass of an inhibitory upstream ORF to enhance CHOP translation. J Biol Chem 286, 10939–10949. 21285359 10.1074/jbc.M110.216093 PMC3064149

[165] Barbosa C , Peixeiro I & Romão L (2013) Gene expression regulation by upstream open reading frames and human disease. PLoS Genet 9, 1–12. 10.1371/journal.pgen.1003529 PMC3738444 23950723

[166] Fornace AJ , Alamo I & Hollander MC (1988) DNA damage‐inducible transcripts in mammalian cells. Proc Natl Acad Sci USA 85, 8800–8804. 3194391 10.1073/pnas.85.23.8800 PMC282594

[167] Fornace AJ , Nebert DW , Hollander MC , Luethy JD , Papathanasiou M , Fargnoli J & Holbrook NJ (1989) Mammalian genes coordinately regulated by growth arrest signals and DNA‐damaging agents. Mol Cell Biol 9, 4196–4203. 2573827 10.1128/mcb.9.10.4196-4203.1989 PMC362498

[168] Lee YY , Cevallos RC & Jan E (2009) An upstream open reading frame regulates translation of GADD34 during cellular stresses that induce eIF2 phosphorylation. J Biol Chem 284, 6661–6673. 19131336 10.1074/jbc.M806735200 PMC2652341

[169] Ma Y & Hendershot LM (2003) Delineation of a negative feedback regulatory loop that controls protein translation during endoplasmic reticulum stress. J Biol Chem 278, 34864–34873. 12840028 10.1074/jbc.M301107200

[170] Marciniak SJ , Yun CY , Oyadomari S , Novoa I , Zhang Y , Jungreis R , Nagata K , Harding HP & Ron D (2004) CHOP induces death by promoting protein synthesis and oxidation in the stressed endoplasmic reticulum. Genes Dev 18, 3066–3077. 15601821 10.1101/gad.1250704 PMC535917

[171] Connor JH , Weiser DC , Li S , John M , Li SHI & Hallenbeck JM (2001) Growth arrest and DNA damage‐inducible protein GADD34 assembles a novel signaling complex containing Protein Phosphatase 1 and inhibitor 1. Mol Cell Biol 21, 6841–6850. 11564868 10.1128/MCB.21.20.6841-6850.2001 PMC99861

[172] Novoa I , Zeng H , Harding HP & Ron D (2001) Feedback inhibition of the unfolded protein response by GADD34‐mediated dephosphorylation of eIF2alpha. J Cell Biol 153, 1011–1022. 11381086 10.1083/jcb.153.5.1011 PMC2174339

[173] Harding HP , Zhang Y , Zeng H , Novoa I , Lu PD , Calfon M , Sadri N , Yun C , Popko B , Paules R *et al* (2003) An integrated stress response regulates amino acid metabolism and resistance to oxidative stress. Mol Cell 11, 619–633. 12667446 10.1016/s1097-2765(03)00105-9

[174] Quirós PM , Prado MA , Zamboni N , D'Amico D , Williams RW , Finley D , Gygi SP & Auwerx J (2017) Multi‐omics analysis identifies ATF4 as a key regulator of the mitochondrial stress response in mammals. J Cell Biol 216, 2027–2045. 28566324 10.1083/jcb.201702058 PMC5496626

[175] Rajesh K , Krishnamoorthy J , Kazimierczak U , Tenkerian C , Papadakis AI , Wang S , Huang S & Koromilas AE (2015) Phosphorylation of the translation initiation factor eIF2α at serine 51 determines the cell fate decisions of Akt in response to oxidative stress. Cell Death Dis 6, e1591. 25590801 10.1038/cddis.2014.554 PMC4669752

[176] Lee IC , Ho XY , George SE , Goh CW , Sundaram JR , Pang KKL , Luo W , Yusoff P , Sze NSK & Shenolikar S (2017) Oxidative stress promotes SIRT1 recruitment to the GADD34/PP1α complex to activate its deacetylase function. Cell Death Differ 25, 255–267. 28984870 10.1038/cdd.2017.152 PMC5762841

[177] Chen D , Fan Z , Rauh M , Buchfelder M , Eyupoglu IY & Savaskan N (2017) ATF4 promotes angiogenesis and neuronal cell death and confers ferroptosis in a xCT‐dependent manner. Oncogene 36, 5593–5608. 28553953 10.1038/onc.2017.146 PMC5633655

[178] Wu Z , Li M , Zheng W , Hu Q , Cheng Z & Guo F (2017) Silencing of both ATF4 and PERK inhibits cell cycle progression and promotes the apoptosis of differentiating chondrocytes. Int J Mol Med 40, 101–111. 28498443 10.3892/ijmm.2017.2985 PMC5466379

[179] Ishizawa J , Kojima K , Chachad D , Ruvolo P , Ruvolo V , Jacamo RO , Borthakur G , Mu H , Zeng Z , Tabe Y *et al* (2016) ATF4 induction through an atypical integrated stress response to ONC201 triggers p53‐independent apoptosis in hematological malignancies. Sci Signal 9, ra17. 26884599 10.1126/scisignal.aac4380 PMC4815038

[180] Iurlaro R , Püschel F , León‐Annicchiarico CL , O'Connor H , Martin SJ , Palou‐Gramón D , Lucendo E & Muñoz‐Pinedo C (2017) Glucose deprivation induces ATF4‐mediated apoptosis through TRAIL death receptors. Mol Cell Biol 37, e00479–16. 10.1128/MCB.00479-16 28242652 PMC5477549

[181] Zhu C , Johansen FE & Prywes R (1997) Interaction of ATF6 and serum response factor. Mol Cell Biol 17, 4957–4966. 9271374 10.1128/mcb.17.9.4957 PMC232347

[182] Yamamoto K , Sato T , Matsui T , Sato M , Okada T , Yoshida H , Harada A & Mori K (2007) Transcriptional induction of mammalian ER quality control proteins is mediated by single or combined action of ATF6alpha and XBP1. Dev Cell 13, 365–376. 17765680 10.1016/j.devcel.2007.07.018

[183] Asada R , Kanemoto S , Kondo S , Saito A & Imaizumi K (2011) The signalling from endoplasmic reticulum‐resident bZIP transcription factors involved in diverse cellular physiology. J Biochem 149, 507–518. 21454302 10.1093/jb/mvr041

[184] McMahon M , Samali A & Chevet E (2017) Regulation of the unfolded protein response by noncoding RNA. Am J Physiol Cell Physiol 313, C243–C254. 28637678 10.1152/ajpcell.00293.2016

[185] Oslowski CM & Urano F (2011) Measuring ER stress and the unfolded protein response using mammalian tissue culture system. Methods Enzymol 490, 71–92. 21266244 10.1016/B978-0-12-385114-7.00004-0 PMC3701721

[186] Yoshida H , Matsui T , Hosokawa N , Kaufman RJ , Nagata K & Mori K (2003) A time‐dependent phase shift in the mammalian unfolded protein response. Dev Cell 4, 265–271. 12586069 10.1016/s1534-5807(03)00022-4

[187] Sano R & Reed JC (2013) ER stress‐induced cell death mechanisms. Biochim Biophys Acta 1833, 3460–3470. 23850759 10.1016/j.bbamcr.2013.06.028 PMC3834229

[188] Bartoszewska S , Kochan K , Madanecki P , Piotrowski A , Ochocka R , Collawn JF & Bartoszewski R (2013) Regulation of the unfolded protein response by microRNAs. Cell Mol Biol Lett 18, 555–578. 24092331 10.2478/s11658-013-0106-z PMC3877167

[189] Wortel IMN , van der Meer LT , Kilberg MS & van Leeuwen FN (2017) Surviving stress: modulation of ATF4‐mediated stress responses in normal and malignant cells. Trends Endocrinol Metab 28, 794–806. 28797581 10.1016/j.tem.2017.07.003 PMC5951684

[190] Gorman AM , Healy SJ , Jager R & Samali A (2012) Stress management at the ER: regulators of ER stress‐induced apoptosis. Pharmacol Ther 134, 306–316. 22387231 10.1016/j.pharmthera.2012.02.003

[191] Ron D & Walter P (2007) Signal integration in the endoplasmic reticulum unfolded protein response. Nat Rev Mol Cell Biol 8, 519–529. 17565364 10.1038/nrm2199

[192] Hoyer‐Hansen M & Jaattela M (2007) Connecting endoplasmic reticulum stress to autophagy by unfolded protein response and calcium. Cell Death Differ 14, 1576–1582. 17612585 10.1038/sj.cdd.4402200

[193] Cheng X , Liu H , Jiang CC , Fang L , Chen C , Zhang XD & Jiang ZW (2014) Connecting endoplasmic reticulum stress to autophagy through IRE1/JNK/beclin‐1 in breast cancer cells. Int J Mol Med 34, 772–781. 24970676 10.3892/ijmm.2014.1822

[194] Giorgi C , Missiroli S , Patergnani S , Duszynski J , Wieckowski MR & Pinton P (2015) Mitochondria‐associated membranes: composition, molecular mechanisms, and physiopathological implications. Antioxid Redox Signal 22, 995–1019. 25557408 10.1089/ars.2014.6223

[195] Santel A & Fuller MT (2001) Control of mitochondrial morphology by a human mitofusin. J Cell Sci 114, 867–874. 11181170 10.1242/jcs.114.5.867

[196] Muñoz JP , Ivanova S , Sánchez‐Wandelmer J , Martínez‐Cristóbal P , Noguera E , Sancho A , Díaz‐Ramos A , Hernández‐Alvarez MI , Sebastián D , Mauvezin C *et al* (2014) Erratum: Mfn2 modulates the UPR and mitochondrial function via repression of PERK (EMBO Journal 32 (2348‐2361) 10.1038/emboj.2013.168). EMBO J 33, 171. PMC3770335 23921556

[197] Verfaillie T , Rubio N , Garg AD , Bultynck G , Rizzuto R , Decuypere JP , Piette J , Linehan C , Gupta S , Samali A *et al* (2012) PERK is required at the ER‐mitochondrial contact sites to convey apoptosis after ROS‐based ER stress. Cell Death Differ 19, 1880–1891. 22705852 10.1038/cdd.2012.74 PMC3469056

[198] Mori T , Hayashi T , Hayashi E & Su TP (2013) Sigma‐1 receptor chaperone at the er‐mitochondrion interface mediates the mitochondrion‐er‐nucleus signaling for cellular survival. PLoS ONE 8, e76941. 24204710 10.1371/journal.pone.0076941 PMC3799859

[199] Lisbona F , Rojas‐Rivera D , Thielen P , Zamorano S , Todd D , Martinon F , Glavic A , Kress C , Lin JH , Walter P *et al* (2009) BAX inhibitor‐1 is a negative regulator of the ER stress sensor IRE1alpha. Mol Cell 33, 679–691. 19328063 10.1016/j.molcel.2009.02.017 PMC2818874

[200] Vannuvel K , Renard P , Raes M & Arnould T (2013) Functional and morphological impact of ER stress on mitochondria. J Cell Physiol 228, 1802–1818. 23629871 10.1002/jcp.24360

[201] Bravo R , Vicencio JM , Parra V , Troncoso R , Munoz JP , Bui M , Quiroga C , Rodriguez AE , Verdejo HE , Ferreira J *et al* (2011) Increased ER‐mitochondrial coupling promotes mitochondrial respiration and bioenergetics during early phases of ER stress. J Cell Sci 124, 2511. 10.1242/jcs.080762 PMC3113668 21628424

[202] Cullinan SB & Diehl JA (2004) PERK‐dependent activation of Nrf2 contributes to redox homeostasis and cell survival following endoplasmic reticulum stress. J Biol Chem 279, 20108–20117. 14978030 10.1074/jbc.M314219200

[203] Wang C , Li H , Meng Q , Du Y , Xiao F , Zhang Q , Yu J , Li K , Chen S , Huang Z *et al* (2014) ATF4 deficiency protects hepatocytes from oxidative stress via inhibiting CYP2E1 expression. J Cell Mol Med 18, 80–90. 24373582 10.1111/jcmm.12166 PMC3916120

[204] Li G , Mongillo M , Chin KT , Harding H , Ron D , Marks AR & Tabas I (2009) Role of ERO1‐α‐mediated stimulation of inositol 1,4,5‐triphosphate receptor activity in endoplasmic reticulum stress‐induced apoptosis. J Cell Biol 186, 783–792. 19752026 10.1083/jcb.200904060 PMC2753154

[205] Eletto D , Chevet E , Argon Y & Appenzeller‐Herzog C (2014) Redox controls UPR to control redox. J Cell Sci 127, 3649–3658. 25107370 10.1242/jcs.153643

[206] Appenzeller‐Herzog C & Hall MN (2012) Bidirectional crosstalk between endoplasmic reticulum stress and mTOR signaling. Trends Cell Biol 22, 274–282. 22444729 10.1016/j.tcb.2012.02.006

[207] Patricia F , Julien B , Justine L , Patrick M , Joëlle F , Cécile V , Thomas W , Serge M , Colette R , Jean‐Yves S *et al* (2017) mTOR inhibitors activate PERK signaling and favor viability of gastrointestinal neuroendocrine cell lines. Oncotarget 8, 20974–20987. 28423496 10.18632/oncotarget.15469 PMC5400559

[208] Tenkerian C , Krishnamoorthy J , Mounir Z , Kazimierczak U , Khoutorsky A , Staschke KA , Kristof AS , Wang S , Hatzoglou M & Koromilas AE (2015) mTORC2 balances AKT activation and eIF2α serine 51 phosphorylation to promote survival under stress. Mol Cancer Res 13, 1377–1388. 26130148 10.1158/1541-7786.MCR-15-0184-T

[209] Feng B , Yao PM , Li Y , Devlin CM , Zhang D , Harding HP , Sweeney M , Rong JX , Kuriakose G , Fisher EA *et al* (2003) The endoplasmic reticulum is the site of cholesterol‐induced cytotoxicity in macrophages. Nat Cell Biol 5, 781–792. 12907943 10.1038/ncb1035

[210] Fu S , Yang L , Li P , Hofmann O , Dicker L , Hide W , Lin X , Watkins SM , Ivanov AR & Hotamisligil GS (2011) Aberrant lipid metabolism disrupts calcium homeostasis causing liver endoplasmic reticulum stress in obesity. Nature 473, 528–531. 21532591 10.1038/nature09968 PMC3102791

[211] Kaplowitz N , Than TA , Ph D , Shinohara M & Ji C (2007) Endoplasmic reticulum stress and liver injury. Semin Liver Dis 27, 367–377. 17979073 10.1055/s-2007-991513

[212] Oyadomari S , Harding HP , Zhang Y , Oyadomari M & Ron D (2008) De‐phosphorylation of translation initiation factor 2α (eIF2α) enhances glucose tolerance and attenuates hepato‐steatosis in mice. Cell Metab 7, 520–532. 18522833 10.1016/j.cmet.2008.04.011 PMC2474721

[213] Li H , Meng Q , Xiao F , Chen S , Du Y , Yu J , Wang C & Guo F (2011) ATF4 deficiency protects mice from high‐carbohydrate‐diet‐induced liver steatosis. Biochem J 438, 283–289. 21644928 10.1042/BJ20110263

[214] Xiao G , Zhang T , Yu S , Lee S , Calabuig‐Navarro V , Yamauchi J , Ringquist S & Dong HH (2013) ATF4 protein deficiency protects against high fructose‐induced hypertriglyceridemia in mice. J Biol Chem 288, 25350–25361. 23888053 10.1074/jbc.M113.470526 PMC3757199

[215] Wang S , Chen Z , Lam V , Han J , Hassler J , Finck BN , Davidson NO & Kaufman RJ (2012) IRE1α‐XBP1s induces PDI expression to increase MTP activity for hepatic VLDL assembly and lipid homeostasis. Cell Metab 16, 473–486. 23040069 10.1016/j.cmet.2012.09.003 PMC3569089

[216] Lee AH , Scapa EF , Cohen DE & Glimcher LH (2008) Regulation of hepatic lipogenesis by the transcription factor XBP1. Science (80‐) 320, 1492–1496. 10.1126/science.1158042 PMC3620093 18556558

[217] Yi H , Gu C , Li M , Zhang Z , Li Q , Feng J , Zhou J & Du J (2017) PERK/eIF2α contributes to changes of insulin signaling in HepG2 cell induced by intermittent hypoxia. Life Sci 181, 17–22. 28465244 10.1016/j.lfs.2017.04.022

[218] Hosogai N , Fukuhara A , Oshima K , Miyata Y , Tanaka S , Segawa K , Furukawa S , Tochino Y , Komuro R , Matsuda M *et al* (2007) Adipose tissue hypoxia in obesity and its impact on adipocytokine dysregulation. Diabetes 56, 901–911. 17395738 10.2337/db06-0911

[219] Kim B , Kim MS & Hyun CK (2017) Syringin attenuates insulin resistance via adiponectin‐mediated suppression of low‐grade chronic inflammation and ER stress in high‐fat diet‐fed mice. Biochem Biophys Res Commun 488, 40–45. 28476623 10.1016/j.bbrc.2017.05.003

[220] Lipson KL , Fonseca SG , Ishigaki S , Nguyen LX , Foss E , Bortell R , Rossini AA & Urano F (2006) Regulation of insulin biosynthesis in pancreatic beta cells by an endoplasmic reticulum‐resident protein kinase IRE1. Cell Metab 4, 245–254. 16950141 10.1016/j.cmet.2006.07.007

[221] Lee A‐H , Heidtman K , Hotamisligil GS & Glimcher LH (2011) Dual and opposing roles of the unfolded protein response regulated by IRE1alpha and XBP1 in proinsulin processing and insulin secretion. Proc Natl Acad Sci 108, 8885–8890. 21555585 10.1073/pnas.1105564108 PMC3102350

[222] Seo H‐Y , Kim YD , Lee K‐M , Min A‐K , Kim M‐K , Kim H‐S , Won K‐C , Park J‐Y , Lee K‐U , Choi H‐S *et al* (2008) Endoplasmic reticulum stress‐induced activation of activating transcription factor 6 decreases insulin gene expression via up‐regulation of orphan nuclear receptor small heterodimer partner. Endocrinology 149, 3832–3841. 18450959 10.1210/en.2008-0015 PMC2488228

[223] Shao M , Shan B , Liu Y , Deng Y , Yan C , Wu Y , Mao T , Qiu Y , Zhou Y , Jiang S *et al* (2014) Hepatic IRE1α regulates fasting‐induced metabolic adaptive programs through the XBP1s–PPARα axis signalling. Nat Commun 5, 3528. 24670948 10.1038/ncomms4528

[224] Kim H , Tu HC , Ren D , Takeuchi O , Jeffers JR , Zambetti GP , Hsieh JJ & Cheng EH (2009) Stepwise activation of BAX and BAK by tBID, BIM, and PUMA initiates mitochondrial apoptosis. Mol Cell 36, 487–499. 19917256 10.1016/j.molcel.2009.09.030 PMC3163439

[225] Gomez‐Bougie P , Halliez M , Moreau P , Pellat‐Deceunynck C & Amiot M (2016) Repression of Mcl‐1 and disruption of the Mcl‐1/Bak interaction in myeloma cells couple ER stress to mitochondrial apoptosis. Cancer Lett 383, 204–211. 27697610 10.1016/j.canlet.2016.09.030

[226] Li J , Lee B & Lee AS (2006) Endoplasmic reticulum stress‐induced apoptosis: multiple pathways and activation of p53‐up‐regulated modulator of apoptosis (PUMA) and NOXA by p53. J Biol Chem 281, 7260–7270. 16407291 10.1074/jbc.M509868200

[227] Ogata M , Hino S , Saito A , Morikawa K , Kondo S , Kanemoto S , Murakami T , Taniguchi M , Tanii I , Yoshinaga K *et al* (2006) Autophagy is activated for cell survival after endoplasmic reticulum stress. Mol Cell Biol 26, 9220–9231. 17030611 10.1128/MCB.01453-06 PMC1698520

[228] Yu L , Alva A , Su H , Dutt P , Freundt E , Welsh S , Baehrecke EH & Lenardo MJ (2004) Regulation of an ATG7‐beclin 1 program of autophagic cell death by caspase‐8. Science (80‐.) 304, 1500–1502. 10.1126/science.1096645 15131264

[229] Kim I , Shu CW , Xu W , Shiau CW , Grant D , Vasile S , Cosford ND & Reed JC (2009) Chemical biology investigation of cell death pathways activated by endoplasmic reticulum stress reveals cytoprotective modulators of ASK1. J Biol Chem 284, 1593–1603. 19004820 10.1074/jbc.M807308200 PMC2615512

[230] Deng X , Xiao L , Lang W , Gao F , Ruvolo P & May WS (2001) Novel Role for JNK as a Stress‐activated Bcl2 Kinase. J Biol Chem 276, 23681–23688. 11323415 10.1074/jbc.M100279200

[231] Puthalakath H , O'Reilly LA , Gunn P , Lee L , Kelly PN , Huntington ND , Hughes PD , Michalak EM , McKimm‐Breschkin J , Motoyama N *et al* (2007) ER stress triggers apoptosis by activating BH3‐only protein Bim. Cell 129, 1337–1349. 17604722 10.1016/j.cell.2007.04.027

[232] Yamaguchi H & Wang H‐G (2004) CHOP is involved in endoplasmic reticulum stress‐induced apoptosis by enhancing DR5 expression in human carcinoma cells. J Biol Chem 279, 45495–45502. 15322075 10.1074/jbc.M406933200

[233] Maytin EV , Ubeda M , Lin JC & Habener JF (2001) Stress‐Inducible Transcription Factor CHOP/gadd153 Induces Apoptosis in Mammalian Cells via p38 Kinase‐Dependent and ‐Independent Mechanisms. Exp Cell Res 267, 193–204. 11426938 10.1006/excr.2001.5248

[234] Wei S‐G , Yu Y , Weiss RM & Felder RB (2016) Endoplasmic reticulum stress increases brain MAPK signaling, inflammation and renin‐angiotensin system activity and sympathetic nerve activity in heart failure. Am J Physiol Heart Circ Physiol 311, H871–H880. 27496879 10.1152/ajpheart.00362.2016 PMC5114465

[235] Nolan K , Walter F , Tuffy LP , Poeschel S , Gallagher R , Haunsberger S , Bray I , Stallings RL , Concannon CG & Prehn JH (2016) Endoplasmic reticulum stress‐mediated upregulation of miR‐29a enhances sensitivity to neuronal apoptosis. Eur J Neurosci 43, 640–652. 26750440 10.1111/ejn.13160

[236] Wu Y , Li X , Jia J , Zhang Y , Li J , Zhu Z , Wang H , Tang J & Hu J (2018) Transmembrane E3 ligase RNF183 mediates ER stress‐induced apoptosis by degrading Bcl‐xL. Proc Natl Acad Sci USA 115, E2762–E2771. 29507230 10.1073/pnas.1716439115 PMC5866564

[237] Galluzzi L , Buqué A , Kepp O , Zitvogel L & Kroemer G (2016) Immunogenic cell death in cancer and infectious disease. Nat Rev Immunol 17, 97. 27748397 10.1038/nri.2016.107

[238] Garg AD & Agostinis P (2017) Cell death and immunity in cancer: from danger signals to mimicry of pathogen defense responses. Immunol Rev 280, 126–148. 29027218 10.1111/imr.12574

[239] Fan H , Tang HB , Kang J , Shan L , Song H , Zhu K , Wang J , Ju G & Wang YZ (2015) Involvement of endoplasmic reticulum stress in the necroptosis of microglia/macrophages after spinal cord injury. Neuroscience 311, 362–373. 26523978 10.1016/j.neuroscience.2015.10.049

[240] Linkermann A & Green DR (2014) Necroptosis. N Engl J Med 370, 455–465. 24476434 10.1056/NEJMra1310050 PMC4035222

[241] Saveljeva S , Mc Laughlin SL , Vandenabeele P , Samali A & Bertrand MJ (2015) Endoplasmic reticulum stress induces ligand‐independent TNFR1‐mediated necroptosis in L929 cells. Cell Death Dis 6, e1587. 25569104 10.1038/cddis.2014.548 PMC4669746

[242] Pattingre S , Tassa A , Qu X , Garuti R , Liang XH , Mizushima N , Packer M , Schneider MD & Levine B (2005) Bcl‐2 antiapoptotic proteins inhibit Beclin 1‐dependent autophagy. Cell 122, 927–939. 16179260 10.1016/j.cell.2005.07.002

[243] Gandin V , Pellei M , Tisato F , Porchia M , Santini C & Marzano C (2012) A novel copper complex induces paraptosis in colon cancer cells via the activation of ER stress signalling. J Cell Mol Med 16, 142–151. 21388518 10.1111/j.1582-4934.2011.01292.x PMC3823100

[244] Fan J , Long H , Li Y , Liu Y , Zhou W , Li Q , Yin G , Zhang N & Cai W (2013) Edaravone protects against glutamate‐induced PERK/EIF2α/ATF4 integrated stress response and activation of caspase‐12. Brain Res 1519, 1–8. 23648361 10.1016/j.brainres.2013.04.037

[245] Akamatsu K , Shibata M‐A , Ito Y , Sohma Y , Azuma H & Otsuki Y (2009) Riluzole induces apoptotic cell death in human prostate cancer cells via endoplasmic reticulum stress. Anticancer Res 29, 2195–2204. 19528481

[246] Cao J , Dai DL , Yao L , Yu HH , Ning B , Zhang Q , Chen J , Cheng WH , Shen W & Yang ZX (2012) Saturated fatty acid induction of endoplasmic reticulum stress and apoptosis in human liver cells via the PERK/ATF4/CHOP signaling pathway. Mol Cell Biochem 364, 115–129. 22246806 10.1007/s11010-011-1211-9

[247] Du S , Zhou J , Jia Y & Huang K (2010) SelK is a novel ER stress‐regulated protein and protects HepG2 cells from ER stress agent‐induced apoptosis. Arch Biochem Biophys 502, 137–143. 20692228 10.1016/j.abb.2010.08.001

[248] Li S , Zhao F , Cheng S , Wang X & Hao Y (2013) Uric acid‐induced endoplasmic reticulum stress triggers phenotypic change in rat glomerular mesangial cells. Nephrology 18, 682–689. 23841795 10.1111/nep.12127

[249] Apostolou A , Shen Y , Liang Y , Luo J & Fang S (2008) Armet, a UPR‐upregulated protein, inhibits cell proliferation and ER stress‐induced cell death. Exp Cell Res 314, 2454–2467. 18561914 10.1016/j.yexcr.2008.05.001 PMC6719340

[250] Morishima N , Nakanishi K , Takenouchi H , Shibata T & Yasuhiko Y (2002) An endoplasmic reticulum stress‐specific caspase cascade in apoptosis. Cytochrome c‐independent activation of caspase‐9 by caspase‐12. J Biol Chem 277, 34287–34294. 12097332 10.1074/jbc.M204973200

[251] Saito A , Ochiai K , Kondo S , Tsumagari K , Murakami T , Cavener DR & Imaizumi K (2011) Endoplasmic reticulum stress response mediated by the PERK‐eIF2(alpha)‐ATF4 pathway is involved in osteoblast differentiation induced by BMP2. J Biol Chem 286, 4809–4818. 21135100 10.1074/jbc.M110.152900 PMC3039352

[252] Chang YJ , Chen WY , Huang CY , Liu HH & Wei PL (2015) Glucose‐regulated protein 78 (GRP78) regulates colon cancer metastasis through EMT biomarkers and the NRF‐2/HO‐1 pathway. Tumor Biol 36, 1859–1869. 10.1007/s13277-014-2788-x 25431258

[253] Tanjore H , Cheng D‐S , Degryse AL , Zoz DF , Abdolrasulnia R , Lawson WE & Blackwell TS (2011) Alveolar epithelial cells undergo epithelial‐to‐mesenchymal transition in response to endoplasmic reticulum stress. J Biol Chem 286, 30972–30980. 21757695 10.1074/jbc.M110.181164 PMC3162456

[254] Mo XT , Zhou WC , Cui WH , Li DL , Li LC , Xu L , Zhao P & Gao J (2015) Inositol‐requiring protein 1 ‐ X‐box‐binding protein 1 pathway promotes epithelial‐mesenchymal transition via mediating snail expression in pulmonary fibrosis. Int J Biochem Cell Biol 65, 230–238. 26065400 10.1016/j.biocel.2015.06.006

[255] Minchenko DO , Kharkova AP , Halkin OV , Karbovskyi LL & Minchenko OH (2016) Effect of hypoxia on the expression of genes encoding insulin‐like growth factors and some related proteins in u87 glioma cells without IRE1 function. Endocr Regul 50, 43–54. 27560636 10.1515/enr-2016-0008

[256] Li H , Chen X , Gao Y , Wu J , Zeng F & Song F (2015) XBP1 induces snail expression to promote epithelial‐ to‐mesenchymal transition and invasion of breast cancer cells. Cell Signal 27, 82–89. 25280941 10.1016/j.cellsig.2014.09.018

[257] Jin C , Jin Z , Chen N‐Z , Lu M , Liu C‐B , Hu W‐L & Zheng C‐G (2016) Activation of IRE1α‐XBP1 pathway induces cell proliferation and invasion in colorectal carcinoma. Biochem Biophys Res Commun 470, 75–81. 26742428 10.1016/j.bbrc.2015.12.119

[258] Feng YX , Sokol ES , Del Vecchio CA , Sanduja S , Claessen JHL , Proia TA , Jin DX , Reinhardt F , Ploegh HL , Wang Q *et al* (2014) Epithelial‐to‐mesenchymal transition activates PERK‐eIF2α and sensitizes cells to endoplasmic reticulum stress. Cancer Discov 4, 702–715. 24705811 10.1158/2159-8290.CD-13-0945

[259] Moon SY , Kim HS , Nho KW , Jang YJ & Lee SK (2014) Endoplasmic reticulum stress induces epithelial‐mesenchymal transition through autophagy via activation of c‐Src kinase. Nephron Exp Nephrol 126, 127–140. 24863135 10.1159/000362457

[260] Greenwood M , Bordieri L , Greenwood MP , Rosso Melo M , Colombari DS , Colombari E , Paton JFR & Murphy D (2014) Transcription factor CREB3L1 regulates vasopressin gene expression in the rat hypothalamus. J Neurosci 34, 3810–3820. 24623760 10.1523/JNEUROSCI.4343-13.2014 PMC3951688

[261] Azuma Y , Hagiwara D , Lu W , Morishita Y , Suga H , Goto M , Banno R , Sugimura Y , Oyadomari S , Mori K *et al* (2014) Activating transcription factor 6α is required for the vasopressin neuron system to maintain water balance under dehydration in male mice. Endocrinology 155, 4905–4914. 25203138 10.1210/en.2014-1522

[262] Marwarha G , Claycombe K , Schommer J , Collins D & Ghribi O (2016) Palmitate‐induced endoplasmic reticulum stress and subsequent C/EBPα homologous protein activation attenuates leptin and Insulin‐like growth factor 1 expression in the brain. Cell Signal 28, 1789–1805. 27555288 10.1016/j.cellsig.2016.08.012 PMC5019029

[263] Marwarha G , Dasari B & Ghribi O (2012) Endoplasmic reticulum stress‐induced CHOP activation mediates the down‐regulation of leptin in human neuroblastoma SH‐SY5Y cells treated with the oysterol 27‐hydroxycholesterol. Cell Signal 24, 484–492. 21983012 10.1016/j.cellsig.2011.09.029 PMC3237961

[264] Martínez‐Sánchez N , Seoane‐Collazo P , Contreras C , Varela L , Villarroya J , Rial‐Pensado E , Buqué X , Aurrekoetxea I , Delgado TC , Vázquez‐Martínez R *et al* (2017) Hypothalamic AMPK‐ER Stress‐JNK1 axis mediates the central actions of thyroid hormones on energy balance. Cell Metab 26, 212–229.e12. 28683288 10.1016/j.cmet.2017.06.014 PMC5501726

[265] Zughaier SM , Stauffer BB & McCarty NA (2014) Inflammation and ER stress downregulate BDH2 expression and dysregulate intracellular iron in macrophages. J Immunol Res 2014, 140728. 25762501 10.1155/2014/140728 PMC4267003

[266] Mori K (2009) Signalling pathways in the unfolded protein response: development from yeast to mammals. J Biochem 146, 743–750. 19861400 10.1093/jb/mvp166

[267] Michalak M & Gye MC (2015) Endoplasmic reticulum stress in periimplantation embryos. Clin Exp Reprod Med 42, 1–7. 25874167 10.5653/cerm.2015.42.1.1 PMC4390675

[268] Li J , Chen Z , Gao LY , Colorni A , Ucko M , Fang S & Du SJ (2015) A transgenic zebrafish model for monitoring xbp1 splicing and endoplasmic reticulum stress *in vivo* . Mech Dev 137, 33–44. 25892297 10.1016/j.mod.2015.04.001

[269] Ishikawa T , Kashima M , Nagano AJ , Ishikawa‐Fujiwara T , Kamei Y , Todo T & Mori K (2017) Unfolded protein response transducer IRE1‐mediated signaling independent of XBP1 mRNA splicing is not required for growth and development of medaka fish. Elife 6, 1–29. 10.7554/eLife.26845 PMC5636610 28952924

[270] Richardson CE , Kinkel S & Kim DH (2011) Physiological IRE‐1‐XBP‐1 and PEK‐1 signaling in *Caenorhabditis elegans* larval development and immunity. PLoS Genet 7, 1–10. 10.1371/journal.pgen.1002391 PMC3219621 22125500

[271] Shi W , Xu G , Wang C , Sperber SM , Chen Y , Zhou Q , Deng Y & Zhao H (2015) Heat shock 70‐kDa protein 5 (Hspa5) is essential for pronephros formation by mediating retinoic acid signaling. J Biol Chem 290, 577–589. 25398881 10.1074/jbc.M114.591628 PMC4281759

[272] Luo S , Mao C , Lee B & Lee AS (2006) GRP78/BiP Is required for cell proliferation and protecting the inner cell mass from apoptosis during early mouse embryonic development. Mol Cell Biol 26, 5688–5697. 16847323 10.1128/MCB.00779-06 PMC1592753

[273] Zhang X , Szabo E , Michalak M & Opas M (2007) Endoplasmic reticulum stress during the embryonic development of the central nervous system in the mouse. Int J Dev Neurosci 25, 455–463. 17913437 10.1016/j.ijdevneu.2007.08.007

[274] Kratochvílová K , Moráň L , Paďourová S , Stejskal S , Tesařová L , Šimara P , Hampl A , Koutná I & Vaňhara P (2016) The role of the endoplasmic reticulum stress in stemness, pluripotency and development. Eur J Cell Biol 95, 115–123. 26905505 10.1016/j.ejcb.2016.02.002

[275] Hao L , Vassena R , Wu G , Han Z , Cheng Y , Latham KE & Sapienza C (2009) The unfolded protein response contributes to preimplantation mouse embryo death in the DDK syndrome. Biol Reprod 80, 944–953. 19129515 10.1095/biolreprod.108.072546 PMC2723760

[276] Yang Y , Cheung HH , Tu J , Miu KK & Chan WY (2016) New insights into the unfolded protein response in stem cells. Oncotarget 7, 54010–54027. 27304053 10.18632/oncotarget.9833 PMC5288239

[277] Latham KE (2015) Endoplasmic reticulum stress signaling in mammalian oocytes and embryos: life in balance. Int Rev Cell Mol Biol 316, 227–265. 25805126 10.1016/bs.ircmb.2015.01.005 PMC4617547

[278] Gao Y , Sartori DJ , Li C , Yu Q‐C , Kushner JA , Simon MC & Diehl JA (2012) PERK is required in the adult pancreas and is essential for maintenance of glucose homeostasis. Mol Cell Biol 32, 5129–5139. 23071091 10.1128/MCB.01009-12 PMC3510549

[279] Bettigole SE , Lis R , Adoro S , Lee AH , Spencer LA & Weller PFGL (2015) The transcription factor XBP1 is selectively required for eosinophil differentiation. Nat Immunol 16, 829–837. 26147683 10.1038/ni.3225 PMC4577297

[280] Leung A , Gregory NS , Allen L‐AH & Sluka KA (2017) Regular physical activity prevents chronic pain by altering muscle macrophage phenotype and increasing IL‐10 in mice. Pain 157, 70–79. 10.1097/j.pain.0000000000000312 PMC4685958 26230740

[281] Todd DJ , McHeyzer‐Williams LJ , Kowal C , Lee AH , Volpe BT , Diamond B , McHeyzer‐Williams MG & Glimcher LH (2009) XBP1 governs late events in plasma cell differentiation and is not required for antigen‐specific memory B cell development. J Exp Med 206, 2151–2159. 19752183 10.1084/jem.20090738 PMC2757870

[282] van Galen P , Kreso A , Mbong N , Kent DG , Fitzmaurice T , Chambers JE , Xie S , Laurenti E , Hermans K , Eppert K *et al* (2014) The unfolded protein response governs integrity of the haematopoietic stem‐cell pool during stress. Nature 510, 268–272. 24776803 10.1038/nature13228

[283] Matsuzaki S , Hiratsuka T , Taniguchi M , Shingaki K , Kubo T , Kiya K , Fujiwara T , Kanazawa S , Kanematsu R , Maeda T *et al* (2015) Physiological ER stress mediates the differentiation of fibroblasts. PLoS ONE 10, 1–11. 10.1371/journal.pone.0123578 PMC4416017 25928708

[284] Arensdorf AM , Diedrichs D & Rutkowski DT (2013) Regulation of the transcriptome by ER stress: non‐canonical mechanisms and physiological consequences. Front Genet 4, 1–16. 10.3389/fgene.2013.00256 PMC3844873 24348511

[285] Murakami T , Saito A , Hino S , Kondo S , Kanemoto S , Chihara K , Sekiya H , Tsumagari K , Ochiai K , Yoshinaga K *et al* (2009) Signalling mediated by the endoplasmic reticulum stress transducer OASIS is involved in bone formation. Nat Cell Biol 11, 1205–1211. 19767743 10.1038/ncb1963

[286] Hamamura K & Yokota H (2007) Stress to endoplasmic reticulum of mouse osteoblasts induces apoptosis and transcriptional activation for bone remodeling. FEBS Lett 581, 1769–1774. 17418825 10.1016/j.febslet.2007.03.063 PMC1920705

[287] Cui M , Kanemoto S , Cui X , Kaneko M , Asada R , Matsuhisa K , Tanimoto K , Yoshimoto Y , Shukunami C & Imaizumi K (2015) OASIS modulates hypoxia pathway activity to regulate bone angiogenesis. Sci Rep 5, 16455. 26558437 10.1038/srep16455 PMC4642342

[288] Kondo S , Murakami T , Tatsumi K , Ogata M , Kanemoto S , Otori K , Iseki K , Wanaka A & Imaizumi K (2005) OASIS, a CREB/ATF‐family member, modulates UPR signalling in astrocytes. Nat Cell Biol 7, 186–194. 15665855 10.1038/ncb1213

[289] Asada R , Saito A , Kawasaki N , Kanemoto S , Iwamoto H , Oki M , Miyagi H , Izumi S & Imaizumi K (2012) The endoplasmic reticulum stress transducer OASIS is involved in the terminal differentiation of goblet cells in the large intestine. J Biol Chem 287, 8144–8153. 22262831 10.1074/jbc.M111.332593 PMC3318704

[290] Saito A (2014) Physiological functions of endoplasmic reticulum stress transducer OASIS in central nervous system. Anat Sci Int 89, 11–20. 24242870 10.1007/s12565-013-0214-x PMC3889286

[291] van der Harg JM , van Heest JC , Bangel FN , Patiwael S , van Weering JRT & Scheper W (2017) The UPR reduces glucose metabolism via IRE1 signaling. Biochim Biophys Acta 1864, 655–665. 10.1016/j.bbamcr.2017.01.009 28093214

[292] Topisirovic I & Sonenberg N (2011) mRNA translation and energy metabolism in cancer: the role of the MAPK and mTORC1 Pathways. Cold Spring Harb Symp Quant Biol 76, 355–367. 22123850 10.1101/sqb.2011.76.010785

[293] Lowe CE , Dennis RJ , Obi U , O'Rahilly S & Rochford JJ (2012) Investigating the involvement of the ATF6α pathway of the unfolded protein response in adipogenesis. Int J Obes (Lond) 36, 1248–1251. 22124452 10.1038/ijo.2011.233 PMC3438469

[294] Lipson KL , Ghosh R & Urano F (2008) The role of IRE1α in the degradation of insulin mRNA in pancreatic β‐cells. PLoS ONE 3, 1–7. 10.1371/journal.pone.0001648 PMC2241665 18286202

[295] Zhang N , Yang X , Yuan F , Zhang L , Wang Y , Wang L , Mao Z , Luo J , Zhang H , Zhu WG *et al* (2018) Increased amino acid uptake supports autophagy‐deficient cell survival upon glutamine deprivation. Cell Rep 10, 3006–3020. 10.1016/j.celrep.2018.05.006 29874586

[296] Rubio‐Patiño C , Bossowski JP , De Donatis GM , Mondragón L , Villa E , Aira LE , Chiche J , Mhaidly R , Lebeaupin C , Marchetti S *et al* (2018) Low‐protein diet induces IRE1α‐dependent anticancer immunosurveillance. Cell Metab 27, 828–842. 29551590 10.1016/j.cmet.2018.02.009

[297] Xue Z , He Y , Ye K , Gu Z , Mao Y & Qi L (2011) A conserved structural determinant located at the interdomain region of mammalian inositol‐requiring enzyme 1α. J Biol Chem 286, 30859–30866. 21757700 10.1074/jbc.M111.273714 PMC3162446

[298] Waller DD , Jansen G , Golizeh M , Martel‐Lorion C , Dejgaard K , Shiao TC , Mancuso J , Tsantrizos YS , Roy R , Sebag M *et al* (2016) A covalent cysteine‐targeting kinase inhibitor of Ire1 permits allosteric control of endoribonuclease activity. ChemBioChem 17, 843–851. 26792008 10.1002/cbic.201500485

[299] Wiseman RL , Zhang Y , Lee KPK , Harding HP , Haynes CM , Price J , Sicheri F & Ron D (2010) Flavonol activation defines an unanticipated ligand‐binding site in the kinase‐RNase domain of IRE1. Mol Cell 38, 291–304. 20417606 10.1016/j.molcel.2010.04.001 PMC2864793

[300] Rong J , Pass I , Diaz PW , Ngo TA , Sauer M , Magnuson G , Zeng F‐Y , Hassig CA , Jackson MR , Cosford NDP *et al* (2015) Cell‐based high‐throughput luciferase reporter gene assays for identifying and profiling chemical modulators of endoplasmic reticulum signaling protein, IRE1. J Biomol Screen 20, 1232–1245. 26265713 10.1177/1087057115600414

[301] Feldman HC , Tong M , Wang L , Meza‐Acevedo R , Gobillot TA , Lebedev I , Gliedt MJ , Hari SB , Mitra AK , Backes BJ *et al* (2016) Structural and functional analysis of the allosteric inhibition of IRE1α with ATP‐competitive ligands. ACS Chem Biol 11, 2195–2205.27227314

[302] Harrington PE , Biswas K , Malwitz D , Tasker AS , Mohr C , Andrews KL , Dellamaggiore K , Kendall R , Beckmann H , Jaeckel P *et al* (2015) Unfolded protein response in cancer: IRE1α inhibition by selective kinase ligands does not impair tumor cell viability. ACS Med Chem Lett 6, 68–72. 25589933 10.1021/ml500315b PMC4291719

[303] Wang L , Perera BGK , Hari SB , Bhhatarai B , Backes BJ , Seeliger MA , Schürer SC , Oakes SA , Papa FR & Maly DJ (2012) Divergent allosteric control of the IRE1α endoribonuclease using kinase inhibitors. Nat Chem Biol 8, 982–989. 23086298 10.1038/nchembio.1094 PMC3508346

[304] Jha BK , Polyakova I , Kessler P , Dong B , Dickerman B , Sen GC & Silverman RH (2011) Inhibition of RNase L and RNA‐dependent protein kinase (PKR) by sunitinib impairs antiviral innate immunity. J Biol Chem 286, 26319–26326. 21636578 10.1074/jbc.M111.253443 PMC3143594

[305] Volkmann K , Lucas JL , Vuga D , Wang X , Brumm D , Stiles C , Kriebel D , Der‐Sarkissian A , Krishnan K , Schweitzer C *et al* (2011) Potent and selective inhibitors of the inositol‐requiring enzyme 1 endoribonuclease. J Biol Chem 286, 12743–12755. 21303903 10.1074/jbc.M110.199737 PMC3069474

[306] Cross BC , Bond PJ , Sadowski PG , Jha BK , Zak J , Goodman JM , Silverman RH , Neubert TA , Baxendale IR , Ron D *et al* (2012) The molecular basis for selective inhibition of unconventional mRNA splicing by an IRE1‐binding small molecule. Proc Natl Acad Sci USA 109, E869–E878. 22315414 10.1073/pnas.1115623109 PMC3326519

[307] Mimura N , Fulciniti M , Gorgun G , Tai Y‐T , Cirstea D , Santo L , Hu Y , Fabre C , Minami J , Ohguchi H *et al* (2012) Blockade of XBP1 splicing by inhibition of IRE1 is a promising therapeutic option in multiple myeloma. Blood 119, 5772–5781. 22538852 10.1182/blood-2011-07-366633 PMC3382937

[308] Papandreou I , Denko NC , Olson M , Van Melckebeke H , Lust S , Tam A , Solow‐Cordero DE , Bouley DM , Offner F , Niwa M *et al* (2011) Identification of an Ire1alpha endonuclease specific inhibitor with cytotoxic activity against human multiple myeloma. Blood 117, 1311–1314. 21081713 10.1182/blood-2010-08-303099 PMC3056474

[309] Ri M , Tashiro E , Oikawa D , Shinjo S , Tokuda M , Yokouchi Y , Narita T , Masaki A , Ito A , Ding J *et al* (2012) Identification of Toyocamycin, an agent cytotoxic for multiple myeloma cells, as a potent inhibitor of ER stress‐induced XBP1 mRNA splicing. Blood Cancer J 2, e79. 22852048 10.1038/bcj.2012.26 PMC3408640

[310] Tomasio SM , Harding HP , Ron D , Cross BCS & Bond PJ (2013) Selective inhibition of the unfolded protein response: targeting catalytic sites for Schiff base modification. Mol BioSyst 9, 2408–2416. 23884086 10.1039/c3mb70234k

[311] Jiang D , Lynch C , Medeiros BC , Liedtke M , Bam R , Tam AB , Yang Z , Alagappan M , Abidi P , Le Q‐T *et al* (2016) Identification of Doxorubicin as an Inhibitor of the IRE1α‐XBP1 Axis of the Unfolded Protein Response. Sci Rep 6, 33353. 27634301 10.1038/srep33353 PMC5025885

[312] Park BK , Boobis A , Clarke S , Goldring CEP , Jones D , Kenna JG , Lambert C , Laverty HG , Naisbitt DJ , Nelson S *et al* (2011) Managing the challenge of chemically reactive metabolites in drug development. Nat Rev Drug Discov 10, 292–306. 21455238 10.1038/nrd3408

[313] Wilson AJ (2009) Inhibition of protein–protein interactions using designed molecules. Chem Soc Rev 38, 3289. 20449049 10.1039/b807197g

[314] Atkins C , Liu Q , Minthorn E , Zhang S‐Y , Figueroa DJ , Moss K , Stanley TB , Sanders B , Goetz A , Gaul N *et al* (2013) Characterization of a novel PERK kinase inhibitor with antitumor and antiangiogenic activity. Cancer Res 73, 1993–2002. 23333938 10.1158/0008-5472.CAN-12-3109

[315] Axten JM , Medina JR , Feng Y , Shu A , Romeril SP , Grant SW , Li WH , Heerding DA , Minthorn E , Mencken T *et al* (2012) Discovery of 7‐methyl‐5‐(1‐{[3‐(trifluoromethyl)phenyl]acetyl}‐2,3‐dihydro‐1H‐indol‐5‐yl)‐7H‐pyrrolo[2,3‐d]pyrimidin‐4‐amine (GSK2606414), a potent and selective first‐in‐class inhibitor of protein kinase R (PKR)‐like endoplasmic reticulum kinase (PERK). J Med Chem 55, 7193–7207. 22827572 10.1021/jm300713s

[316] Moreno JA , Halliday M , Molloy C , Radford H , Verity N , Axten JM , Ortori CA , Willis AE , Fischer PM , Barrett DA *et al* (2013) Oral treatment targeting the unfolded protein response prevents neurodegeneration and clinical disease in prion‐infected mice. Sci Transl Med 138, 206ra138. 10.1126/scitranslmed.3006767 24107777

[317] Harding HP , Zeng H , Zhang Y , Jungries R , Chung P , Plesken H , Sabatini DD & Ron D (2001) Diabetes mellitus and exocrine pancreatic dysfunction in perk‐/‐ mice reveals a role for translational control in secretory cell survival. Mol Cell 7, 1153–1163. 11430819 10.1016/s1097-2765(01)00264-7

[318] Rojas‐Rivera D , Delvaeye T , Roelandt R , Nerinckx W , Augustyns K , Vandenabeele P & Bertrand MJ (2017) When PERK inhibitors turn out to be new potent RIPK1 inhibitors: critical issues on the specificity and use of GSK2606414 and GSK2656157. Cell Death Differ 24, 1100–1110. 28452996 10.1038/cdd.2017.58 PMC5442476

[319] Sidrauski C , Tsai JC , Kampmann M , Hearn BR , Vedantham P , Jaishankar P , Sokabe M , Mendez AS , Newton BW , Tang EL *et al* (2015) Pharmacological dimerization and activation of the exchange factor eIF2B antagonizes the integrated stress response. Elife 2015, e07314. 10.7554/eLife.07314 PMC4426669 25875391

[320] Sekine Y , Zyryanova A , Crespillo‐Casado A , Fischer PM , Harding HP & Ron D (2015) Stress responses. Mutations in a translation initiation factor identify the target of a memory‐enhancing compound. Science 348, 1027–1030. 25858979 10.1126/science.aaa6986 PMC4538794

[321] Halliday M , Radford H , Sekine Y , Moreno J , Verity N , Le Quesne J , Ortori CA , Barrett DA , Fromont C , Fischer PM *et al* (2015) Partial restoration of protein synthesis rates by the small molecule ISRIB prevents neurodegeneration without pancreatic toxicity. Cell Death Dis 6, e1672. 25741597 10.1038/cddis.2015.49 PMC4385927

[322] Sidrauski C , McGeachy AM , Ingolia NT & Walter P (2015) The small molecule ISRIB reverses the effects of eIF2α phosphorylation on translation and stress granule assembly. Elife 2015, 1–16. 10.7554/eLife.05033 PMC4341466 25719440

[323] Gallagher CM , Garri C , Cain EL , Ang KKH , Wilson CG , Chen S , Hearn BR , Jaishankar P , Aranda‐Diaz A , Arkin MR *et al* (2016) Ceapins are a new class of unfolded protein response inhibitors, selectively targeting the ATF6α branch. Elife 5, 1–33. 10.7554/eLife.11878 PMC4954757 27435960

[324] Gallagher CM & Walter P (2016) Ceapins inhibit ATF6α signaling by selectively preventing transport of ATF6α to the Golgi apparatus during ER stress. Elife 5, e11880. 27435962 10.7554/eLife.11880 PMC4954756

[325] Bu L , Yu H , Fan L , Li X , Wang F , Liu J , Zhong F , Zhang C , Wei W , Wang H *et al* (2017) Melatonin, a novel selective ATF‐6 inhibitor, induces human hepatoma cell apoptosis through COX‐2 downregulation. World J Gastroenterol 23, 986–998. 28246472 10.3748/wjg.v23.i6.986 PMC5311108

[326] Xu S , Butkevich AN , Yamada R , Zhou Y , Debnath B , Duncan R , Zandi E , Petasis NA & Neamati N (2012) Discovery of an orally active small‐molecule irreversible inhibitor of protein disulfide isomerase for ovarian cancer treatment. Proc Natl Acad Sci 109, 16348–16353. 22988091 10.1073/pnas.1205226109 PMC3479552

[327] Banerjee R , Pace NJ , Brown DR & Weerapana E (2013) 1,3,5‐Triazine as a modular scaffold for covalent inhibitors with streamlined target identification. J Am Chem Soc 135, 2497–2500. 23379904 10.1021/ja400427e

[328] Chevet E , Hetz C & Samali A (2015) Endoplasmic reticulum stress‐activated cell reprogramming in oncogenesis. Cancer Discov 5, 586–597. 25977222 10.1158/2159-8290.CD-14-1490

[329] Okada T , Haze K , Nadanaka S , Yoshida H , Seidah NG , Hirano Y , Sato R , Negishi M & Mori K (2003) A serine protease inhibitor prevents endoplasmic reticulum stress‐induced cleavage but not transport of the membrane‐bound transcription factor ATF6. J Biol Chem 278, 31024–31032. 12782636 10.1074/jbc.M300923200

[330] Doultsinos D , Avril T , Lhomond S , Dejeans N , Guédat P & Chevet E (2017) Control of the unfolded protein response in health and disease. SLAS Discov 22, 787–800. 28453376 10.1177/2472555217701685

[331] Delpino A & Castelli M (2002) The 78 kDa glucose‐regulated protein (GRP78/BIP) is expressed on the cell membrane, is released into cell culture medium and is also present in human peripheral circulation. Biosci Rep 22, 407–420. 12516782 10.1023/a:1020966008615

[332] Chessler SD & Byers PH (1993) BiP binds type I procollagen pro alpha chains with mutations in the carboxyl‐terminal propeptide synthesized by cells from patients with osteogenesis imperfecta. J Biol Chem 268, 18226–18233. 8349698

[333] Booth L , Roberts JL , Cash DR , Tavallai S , Jean S , Fidanza A , Cruz‐Luna T , Siembiba P , Cycon KA , Cornelissen CN *et al* (2015) GRP78/BiP/HSPA5/DNA K is a universal therapeutic target for human disease. J Cell Physiol 230, 1661–1676. 25546329 10.1002/jcp.24919 PMC4402027

[334] Zhu H , Chen X , Chen B , Chen B , Song W , Sun D & Zhao Y (2014) Activating transcription factor 4 promotes esophageal squamous cell carcinoma invasion and metastasis in mice and is associated with poor prognosis in human patients. PLoS ONE 9, 1–11. 10.1371/journal.pone.0103882 PMC4117569 25078779

[335] Yang J , Cheng D , Zhou S , Zhu B , Hu T & Yang Q (2015) Overexpression of X‐Box binding protein 1 (XBP1) correlates to poor prognosis and up‐regulation of PI3K/mTOR in human osteosarcoma. Int J Mol Sci 16, 28635–28646. 26633383 10.3390/ijms161226123 PMC4691070

[336] Bujisic B , De Gassart A , Tallant R , Demaria O , Zaffalon L , Chelbi S , Gilliet M , Bertoni F & Martinon F (2017) Impairment of both IRE1 expression and XBP1 activation is a hallmark of GCB DLBCL and contributes to tumor growth. Blood 129, 2420–2428. 28167662 10.1182/blood-2016-09-741348

[337] Kaser A , Lee A‐H , Franke A , Glickman JN , Zeissig S , Tilg H , Nieuwenhuis EES , Higgins DE , Schreiber S , Glimcher LH *et al* (2008) XBP1 links ER stress to intestinal inflammation and confers genetic risk for human inflammatory bowel disease. Cell 134, 743–756. 18775308 10.1016/j.cell.2008.07.021 PMC2586148

[338] Atkin JD , Farg MA , Walker AK , McLean C , Tomas D & Horne MK (2008) Endoplasmic reticulum stress and induction of the unfolded protein response in human sporadic amyotrophic lateral sclerosis. Neurobiol Dis 30, 400–407. 18440237 10.1016/j.nbd.2008.02.009

[339] Nardo G , Pozzi S , Pignataro M , Lauranzano E , Spano G , Garbelli S , Mantovani S , Marinou K , Papetti L , Monteforte M *et al* (2011) Amyotrophic lateral sclerosis multiprotein biomarkers in peripheral blood mononuclear cells. PLoS ONE 6, e25545. 21998667 10.1371/journal.pone.0025545 PMC3187793

[340] Kim Y , Lee H , Manson SR , Lindahl M , Evans B , Miner JH , Urano F & Chen YM (2016) Mesencephalic astrocyte‐derived neurotrophic factor as a urine biomarker for endoplasmic reticulum stress‐related kidney diseases. J Am Soc Nephrol 27, 2974–2982. 26940092 10.1681/ASN.2014100986 PMC5042655

[341] Mami I , Bouvier N , El Karoui K , Gallazzini M , Rabant M , Laurent‐Puig P , Li S , Tharaux PL , Beaune P , Thervet E *et al* (2016) Angiogenin mediates cell‐autonomous translational control under endoplasmic reticulum stress and attenuates kidney injury. J Am Soc Nephrol 27, 863–876. 26195817 10.1681/ASN.2015020196 PMC4769205

[342] Dadey DYA , Kapoor V , Hoye K , Khudanyan A , Collins A , Thotala D & Hallahan DE (2016) Antibody targeting GRP78 enhances the efficacy of radiation therapy in human glioblastoma and non‐small‐cell lung cancer cell lines and tumor models. Clin Cancer Res 23, 2556–2564. 27815359 10.1158/1078-0432.CCR-16-1935

[343] Lin YG , Shen J , Yoo E , Liu R , Yen HY , Mehta A , Rajaei A , Yang W , Mhawech‐Fauceglia P , DeMayo FJ *et al* (2015) Targeting the glucose‐regulated protein‐78 abrogates Pten‐null driven AKT activation and endometrioid tumorigenesis. Oncogene 34, 5418–5426. 25684138 10.1038/onc.2015.4 PMC4537850

[344] Chen YG , Ashok BT , Liu X , Garikapaty VPS , Mittelman A & Tiwari RK (2003) Induction of heat shock protein gp96 by immune cytokines. Cell Stress Chaperones 8, 242–248. 14984057 10.1379/1466-1268(2003)008<0242:iohspg>2.0.co;2 PMC514877

[345] Zhang K , Peng Z , Huang X , Qiao Z , Wang X , Wang N , Xi H , Cui J , Gao Y , Huang X *et al* (2017) Phase II trial of adjuvant immunotherapy with autologous tumor‐derived Gp96 vaccination in patients with gastric cancer. J Cancer 8, 1826–1832. 28819380 10.7150/jca.18946 PMC5556646

[346] Tian S , Liu Z , Donahue C , Falo LD Jr & You Z (2012) Genetic targeting of the active transcription factor XBP1s to dendritic cells potentiates vaccine‐induced prophylactic and therapeutic antitumor immunity. Mol Ther 20, 432–442. 21934655 10.1038/mt.2011.183 PMC3277233

[347] Miller M , Rosenthal P , Beppu A , James L , Hoffman HM , Tam AB , Taylor A , Mcgeough MD , Pena CA , Niwa M *et al* (2014) ORMDL3 transgenic mice have increased airway remodeling and airway responsiveness characteristic of asthma. J Immunol 192, 3475–3487. 24623133 10.4049/jimmunol.1303047 PMC3981544

[348] Graner MW , Lillehei KO & Katsanis E (2015) Endoplasmic reticulum chaperones and their roles in the immunogenicity of cancer vaccines. Front Oncol 4, 1–12. 10.3389/fonc.2014.00379 PMC4285071 25610811

[349] Graner MW , Zeng Y , Feng H & Katsanis E (2003) Tumor‐derived chaperone‐rich cell lysates are effective therapeutic vaccines against a variety of cancers. Cancer Immunol Immunother 52, 226–234. 12669247 10.1007/s00262-002-0359-2 PMC11033032

[350] Rapp UK & Kaufmann SH (2004) DNA vaccination with gp96‐peptide fusion proteins induces protection against an intracellular bacterial pathogen. Int Immunol 16, 597–605. 15039390 10.1093/intimm/dxh064

[351] Qian J , Hong S , Wang S , Zhang L , Sun L , Wang M , Yang J & Kwak LW (2009) Myeloma cell line – derived, pooled heat shock proteins as a universal vaccine for immunotherapy of multiple myeloma. Blood 114, 3880–3890. 19654406 10.1182/blood-2009-06-227355 PMC2773487

[352] Argon Y & Simen BB (1999) GRP94, an ER chaperone with protein and peptide binding properties. Semin Cell Dev Biol 10, 495–505. 10597632 10.1006/scdb.1999.0320

[353] Liu D , Liu X , Zhou T , Yao W , Zhao J , Zheng Z , Jiang W , Wang F , Aikhionbare FO , Hill DL *et al* (2015) IRE1‐RACK1 axis orchestrates ER stress preconditioning‐elicited cytoprotection from ischemia/reperfusion injury in liver. J Mol Cell Biol 8, 144–156. 26711306 10.1093/jmcb/mjv066 PMC4816147

[354] Bailly‐Maitre B , Fondevila C , Kaldas F , Droin N , Luciano F , Ricci J‐E , Croxton R , Krajewska M , Zapata JM , Kupiec‐Weglinski JW *et al* (2006) Cytoprotective gene bi‐1 is required for intrinsic protection from endoplasmic reticulum stress and ischemia‐reperfusion injury. Proc Natl Acad Sci USA 103, 2809–2814. 16478805 10.1073/pnas.0506854103 PMC1413773

[355] Bi X , Zhang G , Wang X , Nguyen C , May HI , Li X , Al‐Hashimi AA , Austin RC , Gillette TG , Fu G *et al* (2018) Endoplasmic reticulum chaperone GRP78 protects heart from ischemia/reperfusion injury through Akt activation. Circ Res 122, 1545–1554. 29669712 10.1161/CIRCRESAHA.117.312641 PMC5970094

[356] Martindale JJ , Fernandez R , Thuerauf D , Whittaker R , Gude N , Sussman MA & Glembotski CC (2006) Endoplasmic reticulum stress gene induction and protection from ischemia/reperfusion injury in the hearts of transgenic mice with a tamoxifen‐regulated form of ATF6. Circ Res 98, 1186–1193. 16601230 10.1161/01.RES.0000220643.65941.8d

[357] Peralta C & Brenner C (2011) Endoplasmic reticulum stress inhibition enhances liver tolerance to ischemia/reperfusion. Curr Med Chem 18, 2016–2024. 21517773 10.2174/092986711795590039

[358] Folch‐Puy E , Panisello A , Oliva J , Lopez A , Benítez CC , Adam R & Roselló‐Catafau J (2016) Relevance of endoplasmic reticulum stress cell signaling in liver cold ischemia reperfusion injury. Int J Mol Sci 17, 1–12. 10.3390/ijms17060807 PMC4926341 27231901

[359] Kwon SK , Ahn M , Song HJ , Kang SK , Jung SB , Harsha N , Jee S , Moon JY , Suh KS , Do LS *et al* (2015) Nafamostat mesilate attenuates transient focal ischemia/reperfusion‐induced brain injury via the inhibition of endoplasmic reticulum stress. Brain Res 1627, 12–20. 26390938 10.1016/j.brainres.2015.09.013

[360] Zhang H , Yue Y , Sun T , Wu X & Xiong S (2017) Transmissible endoplasmic reticulum stress from myocardiocytes to macrophages is pivotal for the pathogenesis of CVB3‐induced viral myocarditis. Sci Rep 7, 42162. 28176833 10.1038/srep42162 PMC5296968

[361] Rodvold JJ , Chiu KT , Hiramatsu N , Nussbacher JK , Galimberti V , Mahadevan NR , Willert K , Lin JH & Zanetti M (2017) Intercellular transmission of the unfolded protein response promotes survival and drug resistance in cancer cells. Sci Signal 10, eaah7177 10.1126/scisignal.aah7177 28588081 PMC5962022

[362] Brownlie RJ , Myers LK , Wooley PH , Corrigall VM , Bodman‐Smith MD , Panayi GS & Thompson SJ (2006) Treatment of murine collagen‐induced arthritis by the stress protein BiP Via interleukin‐4 – producing regulatory t cells a novel function for an ancient protein. Arthritis Rheumatol 54, 854–863. 10.1002/art.21654 16508967

[363] Kirkham B , Chaabo K , Hall C , Garrood T , Mant T , Allen E , Vincent A , Vasconcelos JC , Prevost AT , Panayi GS *et al* (2016) Safety and patient response as indicated by biomarker changes to binding immunoglobulin protein in the phase I/IIA RAGULA clinical trial in rheumatoid arthritis. Rheumatology (United Kingdom) 55, 1993–2000. 10.1093/rheumatology/kew287 PMC5854092 27498355

[364] Axten JM , Romeril SP , Shu A , Ralph J , Medina JR , Feng Y , Li WHH , Grant SW , Heerding DA , Minthorn E *et al* (2013) Discovery of GSK2656157: an optimized perk inhibitor selected for preclinical development. ACS Med Chem Lett 4, 964–968. 24900593 10.1021/ml400228e PMC4027568

[365] Boyce M , Bryant KF , Jousse C , Long K , Harding HP , Scheuner D , Kaufman RJ , Ma D , Coen DM , Ron D *et al* (2005) A selective inhibitor of eIF2alpha dephosphorylation protects cells from ER stress. Science 307, 935–939. 15705855 10.1126/science.1101902

[366] Saxena S , Cabuy E & Caroni P (2009) A role for motoneuron subtype‐selective ER stress in disease manifestations of FALS mice. Nat Neurosci 12, 627–636. 19330001 10.1038/nn.2297

[367] Colla E , Coune P , Liu Y , Pletnikova O , Troncoso JC , Iwatsubo T , Schneider BL & Lee MK (2012) Endoplasmic reticulum stress is important for the manifestations of α‐synucleinopathy *in vivo* . J Neurosci 32, 3306–3320. 22399753 10.1523/JNEUROSCI.5367-11.2012 PMC3461828

[368] Wang L , Popko B , Tixier E & Roos RP (2014) Guanabenz, which enhances the unfolded protein response, ameliorates mutant SOD1‐induced amyotrophic lateral sclerosis. Neurobiol Dis 71, 317–324. 25134731 10.1016/j.nbd.2014.08.010 PMC4179984

[369] Das I , Krzyzosiak A , Schneider K , Wrabetz L , D'Antonio M , Barry N & Sigurdardottir ABA (2015) Preventing proteostasis diseases by selective inhibition of a phosphatase regulatory subunit. Science 348, 239–242. 25859045 10.1126/science.aaa4484 PMC4490275

[370] Lee A‐H , Iwakoshi NN & Glimcher LH (2003) XBP‐1 regulates a subset of endoplasmic reticulum resident chaperone genes in the unfolded protein response. Mol Cell Biol 23, 7448–7459. 14559994 10.1128/MCB.23.21.7448-7459.2003 PMC207643

[371] Ghosh R , Wang L , Wang ES , Perera BGK , Igbaria A , Morita S , Prado K , Thamsen M , Caswell D , Macias H *et al* (2014) Allosteric inhibition of the IRE1α RNase preserves cell viability and function during endoplasmic reticulum stress. Cell 158, 534–548. 25018104 10.1016/j.cell.2014.07.002 PMC4244221

[372] Kawamura T , Tashiro E , Shindo K & Imoto M (2008) SAR study of a novel triene‐ansamycin group compound, quinotrierixin, and related compounds, as inhibitors of ER stress‐induced XBP1 activation II. Structure elucidation. J Antibiot (Tokyo) 61, 312–317. 18653997 10.1038/ja.2008.44

[373] Chen D , Landis‐Piwowar KR , Chen MS & Dou QP (2007) Inhibition of proteasome activity by the dietary flavonoid apigenin is associated with growth inhibition in cultured breast cancer cells and xenografts. Breast Cancer Res 9, R80. 18300387 10.1186/bcr1797 PMC2246179

[374] Zhu M , Rajamani S , Kaylor J , Han S , Zhou F & Fink AL (2004) The flavonoid baicalein inhibits fibrillation of alpha‐synuclein and disaggregates existing fibrils. J Biol Chem 279, 26846–26857. 15096521 10.1074/jbc.M403129200

[375] Kim D‐S , Ha K‐C , Kwon D‐Y , Kim M‐S , Kim H‐R , Chae S‐W & Chae H‐J (2008) Kaempferol protects ischemia/reperfusion‐induced cardiac damage through the regulation of endoplasmic reticulum stress. Immunopharmacol Immunotoxicol 30, 257–270. 18569083 10.1080/08923970701812530

[376] Plate L , Cooley CB , Chen JJ , Paxman RJ , Gallagher CM , Madoux F , Genereux JC , Dobbs W , Garza D , Spicer TP *et al* (2016) Small molecule proteostasis regulators that reprogram the ER to reduce extracellular protein aggregation. Elife 5, e15550. 27435961 10.7554/eLife.15550 PMC4954754

[377] Higa A , Taouji S , Lhomond S , Jensen D , Fernandez‐Zapico ME , Simpson JC , Pasquet JM , Schekman R & Chevet E (2014) Endoplasmic reticulum stress‐activated transcription factor atf6α requires the disulfide isomerase PDIA5 to modulate chemoresistance. Mol Cell Biol 34, 1839–1849. 24636989 10.1128/MCB.01484-13 PMC4019026

